# Genome-Wide SNP Identification and Association Mapping for Seed Mineral Concentration in Mung Bean (*Vigna radiata* L.)

**DOI:** 10.3389/fgene.2020.00656

**Published:** 2020-06-24

**Authors:** Xingbo Wu, A. S. M. Faridul Islam, Naransa Limpot, Lucas Mackasmiel, Jerzy Mierzwa, Andrés J. Cortés, Matthew W. Blair

**Affiliations:** ^1^Department of Agricultural and Environmental Sciences, Tennessee State University, Nashville, TN, United States; ^2^Agro-Biotechnology Institute, Selangor, Malaysia; ^3^Corporación Colombiana de Investigación Agropecuaria AGROSAVIA, C.I. La Selva, Rionegro, Colombia; ^4^Departamento de Ciencias Forestales, Facultad de Ciencias Agrarias, Universidad Nacional de Colombia – Sede Medellín, Medellín, Colombia

**Keywords:** association mapping, nutritional improvement, mineral nutrients, biofortification, nutritional breeding, nutrigenomics, micronutrients

## Abstract

Mung bean (*Vigna radiata* L.) quality is dependent on seed chemical composition, which in turn determines the benefits of its consumption for human health and nutrition. While mung bean is rich in a range of nutritional components, such as protein, carbohydrates and vitamins, it remains less well studied than other legume crops in terms of micronutrients. In addition, mung bean genomics and genetic resources are relatively sparse. The objectives of this research were three-fold, namely: to develop a genome-wide marker system for mung bean based on genotyping by sequencing (GBS), to evaluate diversity of mung beans available to breeders in the United States and finally, to perform a genome-wide association study (GWAS) for nutrient concentrations based on a seven mineral analysis using inductively coupled plasma (ICP) spectroscopy. All parts of our research were performed with 95 cultivated mung bean genotypes chosen from the USDA core collection representing accessions from 13 countries. Overall, we identified a total of 6,486 high quality single nucleotide polymorphisms (SNPs) from the GBS dataset and found 43 marker × trait associations (MTAs) with calcium, iron, potassium, manganese, phosphorous, sulfur or zinc concentrations in mung bean grain produced in either of two consecutive years’ field experiments. The MTAs were scattered across 35 genomic regions explaining on average 22% of the variation for each seed nutrient in each year. Most of the gene regions provided valuable candidate loci to use in future breeding of new varieties of mung bean and further the understanding of genetic control of nutritional properties in the crop. Other SNPs identified in this study will serve as important resources to enable marker-assisted selection (MAS) for nutritional improvement in mung bean and to analyze cultivars of mung bean.

## Introduction

Mung bean (*Vigna radiata*) is an important pulse crop for most of the highly populated countries of Asia, such as Bangladesh, China, India, Indonesia, Laos, Malaysia, Myanmar, Pakistan, Thailand and Vietnam with growing presence in other parts of the world too (Nair et al., 2013). The cultivation area for the crop is over 6 million hectares worldwide (8.5% of the global pulse area), and over 3 million tons of grain are produced (5% of global pulse production) (Schafleitner et al., 2016). High nitrogen fixation gives mung bean a vital role in intercropping with corn or millet in East Asia or for crop rotation with cereals, such as rice or wheat in South and Southeast Asia (Blair et al., 2016c). Production of mung bean usually increases soil fertility for subsequent cereal crop yields through symbiotic Nitrogen fixation. High market demands make mung bean a profitable crop for export from Australia, Europe, and United States to East Asia (Pandey et al., 2018). South Asia is also a developing market for imports from Eastern Africa. Mung bean is the third most important legume in China after soybean and common bean (Li et al., 2017).

Nutritionally, mung bean along with other Asian *Vigna* species, are considered highly nutritious, easy to cook and palatable legumes (Day, 2013; Rehman et al., 2019). Mung bean contains easily digestible proteins and various micronutrients (Akaerue and Onwuka, 2010; Kollárová et al., 2010). The seeds can be consumed as a dry bean or as a vegetable bean sprouts (Kim et al., 2015). They can be shelled from green pods, decorticated when dry and processed into a flour that is used commercially for products such as noodles, flatbreads, drinks and sweets (Dahiya et al., 2014, 2015). Mung bean sprouts are an important vegetable in several Asian cuisines (Lambrides and Godwin, 2007; Somta and Srinives, 2007). The mung bean is less well studied for biofortification traits than some other legumes even though it has a role to play in preventing micronutrient deficiencies (Dwivedi et al., 2012).

Human nutritional deficiencies of some minerals can lead to stunted growth and development in children, lower resistance to disease, and increased mortality rates (Caulfield et al., 2006). Over two-thirds of humans are experiencing inadequate intake of one or more mineral nutrients, with more than half having iron deficiency and over one third zinc deficiency (White and Broadley, 2009). Mineral deficiencies are, therefore, a major concern in global health (Soetan et al., 2010). Nutritional deficiencies are especially prevalent in developing countries where people do not have diverse diet of vegetables, fruits and meat or fish so grain crops are by necessity the major source of essential nutrients for humans (Dwivedi et al., 2012).

Food legumes usually contain higher concentrations of mineral nutrients than do cereals or root crops, but still need improvement for total concentration and bioavailability (Blair, 2013). Biofortification is the process of genetic improvement for increasing mineral acquisition, reducing anti-nutritional factors and balancing the appropriate mineral concentrations in the edible plant parts and seeds (Pfeiffer and McClafferty, 2007; Dwivedi et al., 2012). Quantitative trait loci (QTL) analyses through mapping populations or genome-wide association studies (GWAS) using molecular markers and high throughput sequencing techniques provide valuable ways of identifying the genes underlying nutritional traits (Blair, 2013). Previous QTL studies for mineral nutrients in legumes have been with mapping populations as reported on common bean, *Phaseolus vulgaris* (Blair et al., 2010a, 2011, 2013, 2016c), *Lotus japonicus* (Klein and Grusak, 2009), lentil, *Lens culinaris* (Khazaei et al., 2017; Ates et al., 2018), *Medicago truncatula* (Sankaran et al., 2009), and pea, *Pisum sativum* (Cheng et al., 2015; Diapari et al., 2015; Ma et al., 2017). GWAS for micronutrient concentration has been used for peas (Kwon et al., 2012) but has not been used in most other legume crops.

Despite all the economic and nutritional value of mung beans, the expansion of the crop is limited by a lack of genetic resources that are important for crop adaptation (Das et al., 2018). Breeding of mung beans for wider adaptation is a priority and modern marker tools are needed for selection of new varieties (Burlyaeva et al., 2019). Genetic markers are an important part of modern plant breeding for the legumes. In this regard, mung bean was one of the first legumes to have a genetic linkage map (Fatokun et al., 1992). However, genetic studies of mung bean have been sparse although the release of a whole genome sequence is now helping researchers to study genes from this important legume (Kang et al., 2014).

Few germplasm studies have been published for mung bean because the genetic diversity based on microsatellite (SSR) markers is limited and few applied SSR markers have been developed (Humphry et al., 2002; Chen et al., 2015; Liu et al., 2017) because of low polymorphism (Gwag et al., 2006; Somta et al., 2008; Tangphatsornruang et al., 2009). Single Nucleotide Polymorphism (SNP) markers have emerged as an interesting option for mung bean with over 300,000 discovered by Van et al. (2013). However, only 43 and 20 SNPs have been validated as competitive allele specific polymorphism (KASP) markers in the two studies conducted to date (Van et al., 2013; Islam and Blair, 2018), respectively.

An alternative to single marker assays for SNPs, is Genotyping by Sequencing (GBS) based on the fast development of next generation sequencing (NGS) technology (Poland and Rife, 2012). Compared to other SNP marker assays, GBS can allow genotyping to be completed rapidly and is a simultaneous method for polymorphism discovery (Elshire et al., 2011). The technique has been used extensively for a wide range of applications and crops (He et al., 2014). To date, GBS studies have been undertaken in mung bean for genetic mapping and diversity study (Schafleitner et al., 2016; Noble et al., 2018), but not for association mapping.

In summary, mung bean can effectively contribute to the alleviation of iron, zinc and protein deficiency in human populations of Asia (Singh R. et al., 2017), but so far there is no genetic study of mineral nutrients in the crop. GBS, being a whole-genome method of surveying polymorphism across a collection of genotypes is ideal for gene discovery in mung bean and for finding SNP polymorphisms that can be used to select for biofortified, nutrient dense genotypes. With this in mind, the objectives of this study were to (1) develop genome-wide SNPs for mung bean using a GBS approach, (2) investigate the genetic diversity and population structure of mung bean as it can account for spurious genomic association, and (3) perform genome-wide association studies with seed mineral concentrations in mung bean.

## Materials and Methods

### Plant Material

This study began in 2015 with the evaluation of a core collection of 408 genotypes of mung bean provided by the United States Department of Agriculture (USDA), Plant Genetic Resources Conservation Unit, in Griffin, GA, United States. As not all genotypes were adapted, a smaller set of 140 genotypes were grown in 2016. From these a subset of 95 higher yielding genotypes were selected for molecular and GWAS analyses. The subset had the following geographical representation: 3 genotypes were from China, 6 from the Philippines, 7 from South Korea, 2 from Thailand, 37 from India, 32 from Iran, 2 from Pakistan, and one each from Afghanistan, Argentina, Mexico, Turkey, United States, and Uzbekistan. Therefore, the total from each continent skewed toward Asia, the center of origin for mung bean, over those from the Americas. The genotypes tended to have sub-tropical rather than temperate adaptation because they were selected for growth ability in the hot summer season in the Tennessee State University (TSU) field station as described below.

### Field Site and Experiment Design

The experiments were carried across two consecutive summer seasons of 2015 and 2016 at the Agricultural Research and Education Center (AREC) field station of TSU in Nashville TN, United States of America which is situated at 36° 10′ 1″ N/86° 49′ 45″ W with an elevation of 54.3 m above sea level. The climate conditions were hot and humid (Koppen zone: Cfa, humid subtropical). The experiments were conducted in an open field and only watered once to induce germination. Thereafter, the crop relied upon rainfall for water supply during both years. An augmented design was used in 2015 and a randomized complete block design with two repetitions was used in 2016. A total of 30 seeds per genotype were sown into each plot, and the plots were 2.4 m long with l.0 m row centers and blocks were separated by 0.8 m alleys.

### Soil Analysis of the Site

The soil was a Byler type Silt Loam with 2–5 percent slope toward the West in the direction of the Cumberland River which surrounds the farm of which the field is part of the valley. Water carrying capacity was estimated to be 50 mm of available water per cubic foot. The natural fertility and micronutrient content of the site was tested with a soils analysis at the University of Tennessee – Extension Soil, Plant and Pest Center (Nashville, TN) based on soil collected in the late winter prior to planting of the mung bean experiments. In addition to macronutrients nitrogen (N), phosphorus (P) and Potassium (K), the soils test evaluated Boron (B), Calcium (Ca), Iron (Fe), Magnesium (Mg), Manganese (Mn), Sodium (Na), Sulfur (S), and Zinc (Zn) along with organic matter and pH readings. No fertilizer was used as that could bias micro-nutrient accumulation.

### Seed Mineral Analysis

The seeds from each genotype were harvested from pods, cleaned and dried at room temperature. To analyze mineral concentrations, 20 g of seeds were ground using zirconium oxide grinding balls and Teflon coated contained using a Retsch mix mill 400 (Verder Scientific Inc., Newtown, PA, United States) to grind each sample. A 0.5 g sub-sample for each genotype was measured out for each genotype were digested in CEM tubes containing 10 mL of ultra-pure nitric acid using a MARS 5 Microwaving Digestion Oven (CEM Corporation, Matthews, NC, United States) set at 200°C for 15 min. Reagent blanks were included in each series of digestions. Subsequently, the digested samples were diluted in 50 mL volumetric flasks by using ultrapure water and filtered before analysis. The sample solutions were stored in polyethylene vials at 4°C until analysis. Each sample was then analyzed for seven different elements, Ca, Fe, K, Mn, P, S, and Zn, using inductively coupled plasma optical emission spectroscopy (ICP-OES) with a CIROS Model FCE12 (Spectro Inc., Kleve, Germany) housed in the Soils and Tissue Analysis laboratory of TSU. Mineral concentration was calculated by multiplying the average sample elemental concentration and reported in μg g^–1^ (ppm).

### DNA Extraction

High molecular weight DNA that was also of high quality and purity was needed for the GBS method. In this study, we used the shoot apex and young leaves of mung bean sprouts to obtain soft etiolated leaf tissue that were easily ground to a powder in the Retsch mill using stainless steel balls and grinding chambers cooled to below freezing with liquid Nitrogen. In this method, seeds were sterilized in bleach for 5 min followed by 2 5-min rinses in autoclaved ddH_2_O. Sterilized seeds were then transferred into individual magenta box (Sigma-Aldrich Co. St. Louis, MO, United States) with a sterilized paper tower and placed in a growth chamber (Thermo Fisher Scientific, Inc., Waltham, MA, United States) for 7 days until seeds had sprouted. The ground tissue (approximately 50 mg) was enough for DNA extraction with a DNeasy 96 Plant Kit (QIAGEN, Valencia, CA, United States). Quantity and quality verification for DNA was made with a FLUOstar Omega spectrometer (BMG LABTECH Inc., Cary, NC, United States). A threshold value of 1.8 for 260/280 nm absorbance ratio was considered a threshold to select for high DNA purity and was confirmed by electrophoresis on a 1% agarose gel run at 110V in TAE buffer. A lyophilized aliquot consisting of 1.5 μg of DNA was prepared for each accession and was placed in a 96 well plate for GBS.

### Genotyping by Sequencing, SNP Identification and Physical Mapping

GBS library construction was done at the Cornell University Biotechnology Institute according to Elshire et al. (2011). Briefly, the genomic DNA was digested with *Ape*KI (New England BioLabs Inc., Ipswich, MA, United States), followed by ligation with a 95-plex barcode adaptor and a common Illumina adaptor. DNA fragments selection was done after digestion/ligation using magnetic beads for a size range of 300–400 nt. (Rohland and Reich, 2012). Single-end sequencing was done on a HiSeq 2000 sequencer (Illumina Inc. San Diego CA, United States). Raw sequence data were analyzed with the GBS discovery pipeline in TASSEL (Trait Analysis by aSSociation, Evolution and Linkage) software (Glaubitz et al., 2014). The FASTQ raw files with information of plate layout and bar codes for each genotype, were used to construct a GBS database for SNP identification. The reads containing bar code sequences followed by *Ape*KI sticky end restriction cut sites (CWGC) were trimmed to 64 bases and stored in the database. Reads that had no matching barcode or cut site overhangs were excluded, as well as reads containing unidentified bases (N) and reads with adapter dimers. Subsequently, the bar-coded and tagged sequence reads were sorted and collapsed into unique sequence tags that were aligned with the *Vigna radiata* genome v1.0 from Legume Information System (LIS) at https://legumeinfo.org/genomes, using the Burrows-Wheeler Aligner (BWA) algorithm (Li and Durbin, 2009) for confirmation of SNP loci. The aligned positions of the SNP markers were used in the construction of a physical map for all 11 chromosomes of the mung bean genome based on R v.3.3.1 software showing distance (in Million base pair, Mb) to the left of the map and SNP loci to the right. All newly discovered SNPs were scored for coverage, depth and genotypic information using vcftools. A quality threshold score of 10 was applied for the validation of any given SNP locus. Each new locus was given a name corresponding to SNP for *Vigna radiata* (SVR) identifying its chromosome and position.

### Statistical Analysis, GWAS Evaluation, and Candidate Gene Identification

Histograms and correlation coefficients for mineral concentrations measured in 2 years were calculated and plotted using customized codes in the software R v.3.3.1 (R Core Team). During the marker-based diversity analysis, we examined subpopulation structure with all the SNP markers found across all the mung bean accessions using principal coordinate analysis (PCoA) carried out and diagramed with the same program. Analyses of variance (ANOVAs) were conducted for each experiment independently, distribution normality was checked and correlations between means across years and amongst minerals were calculated also in R v.3. TASSEL was also used to perform association analyses between the SNP markers and the seed minerals Ca, Fe, K, Mn, P, S, and Zn across the mung bean panel in 2 years. Generalized linear models (GLMs) and mixed linear models (MLMs) were compared for each of the nutrients for both years. Combinations of co-variables were used within each model as follows: (1) model with the country of origin and the first two PCoA axes as covariates; (2) model with the first two PCoA axes scores as covariates; (3) model with the country of origin as covariate; and (4) model without covariates. All four MLMs used a centered IBS kinship matrix as a random effect to control for genomic background implementing the EMMA (Efficient Mixed-Model Association) and P3D (Particle-Particle, Particle-Density) algorithms to reduce computing time (Zhang et al., 2010). QQ-plots (quantile-quantile plots) of the P-values were inspected to assess whether excessive numbers of false positives were generated and choose the optimum models. Significant associations were determined using a strict Bonferroni correction of *P*-values at alpha = 0.05, leading to a significance threshold of 7.7 × 10^–6^ (0.05 divided by the number of markers, which was 6,486 SNP loci) or −log_10_ (7.7 × 10^–6^) = 5.11.

GWAS evaluations were done on a per year basis with construction of customized Manhattan diagrams carried out with the R v.3.3.1 software. For genome diversity inspection in the light of associated regions, we used a sliding window approach to determine patterns of overall variation across the genome, using a window size of 1 Mb and a step size of 200 kilo base pairs (Kb). We computed per-window averages of SNP density, Tajima’s D (Tajima, 1989) and nucleotide diversity as measured by π (Nei, 1987). Results were plotted against window midpoints in mega base pairs (Mb). For any SNPs significantly associated with seed mineral concentration we performed a candidate gene search within 10 Kb around the locus using annotated genes for the mung bean genome from LIS. Genes regions with significant SNPs were listed per mineral.

## Results

### Mineral Concentration Variability

The entire USDA mung bean core collection consisting of 408 genotypes was the starting point for our work and was planted in an augmented design in 2015 after a winter rye grass/summer corn rotation. Early planting was done in April of that year to accommodate the variable maturity dates, photoperiod responses and differential growth habits of the diverse set of genotypes. Most genotypes were harvested by September, but some never produced seed. The best adapted genotypes from the initial trial were re-planted with multiplied seed from that year and put into another section of the same field to avoid diseases or pest carry over. The 2016 trial was implemented from June to October with 140 genotypes. Soils analysis based on a sampling and mixing topsoil from across the fields, showed that pH varied only slightly from 6.0 to 5.9, Na salt was low and average organic matter content was 2%. The soil concentration of minerals showed that no liming was necessary and as a result no extra Zn or Fe micro-fertilization was required.

**FIGURE 1:**
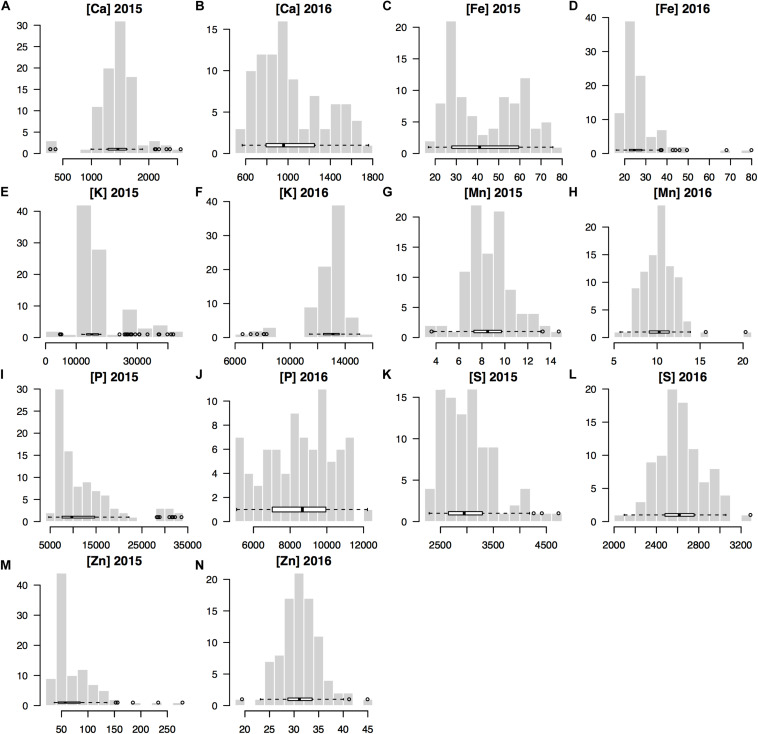
Histograms for the concentrations of seven minerals measured for the seed produced by 95 mung bean (*Vigna radiata*) genotypes harvested across 2 years (2015 and 2016) used in genome wide association studies (GWAS) for Ca, calcium **(A,B)**; Fe, iron **(C,D)**; K, potassium **(E,F)**; Mn, manganes **(G,H)**; P, phosphorus **(I,J)**; S, sulfur **(K,L)**; and Zn, zinc **(M,N)**.

The seed mineral concentrations were measured across both years and resulted in normally distributed data for each year and for most of the seven minerals analyzed for the genotypes used in this GWAS study (Figure 1). Notably, the mean mineral concentrations of mung beans for Fe and Zn were higher in 2015 compared to 2016 (Table 1) and variability was correspondingly higher in the first year for these two micronutrients (Fe = 44.69 ± 17.66 and Zn = 71.75 ± 41.69) than in the second year (Fe = 27.11 ± 9.47 and Zn = 31.27 ± 4.11). This was to be expected, considering that a larger number of genotypes were evaluated in the first year (*n* = 408) compared to the second year (*n* = 95) and the many higher yielding genotypes of the second year compared to the low yielding genotypes which were screened during the first year.

**TABLE 1:** Descriptive statistics and *t*-test comparison for seed mineral concentrations in parts per million (ppm) analyzed in the entire USDA core collection and high yielding selection of mung bean genotypes planted in 2015 (*n* = 408) and 2016 (*n* = 95), respectively.

**Mineral trait**	**Year**	**Population Mean (ppm)**	**Population SD (ppm)**	**Population range (ppm)**	***p* (*t*-test)**
K	2015	17783.48	7774.60	4699.20–41737.90	0.47
	2016	19358.62	19623.72	6542.70–77746.81	
P	2015	12499.70	6852.32	4678.90–33625.30	<0.001
	2016	49124.34	119193.25	5085.90–403256.86	
S	2015	3033.60	510.02	2286.90–4730.10	<0.003
	2016	2613.83	231.78	1978.42–3288.20	
Ca	2015	1463.47	349.40	281.20–2554.90	<0.001
	2016	1038.87	311.80	567.51–1761.96	
Fe	2015	44.69	17.66	16.80–90.10	<0.001
	2016	27.11	9.47	16.04–79.87	
Mn	2015	8.93	2.63	3.60–24.30	0.003
	2016	11.10	6.70	5.69–72.50	
Zn	2015	71.75	41.69	36.30–278.60	<0.001
	2016	31.27	4.11	19.40–44.95	

By comparison, the means for the seed macronutrient concentrations for K and P were higher in 2016 than in 2015 primarily due to the within field locations used since the experiments were planted in the same field across years; however, the smaller RCBD experiment in 2016 was planted in the lower half and higher nutrient section of that field while the augmented design experiment occupied the entire field in 2015. The mean concentrations of the seed minerals Ca Mn and S were similar in both years given that the sections of the field used for the two trials were of the same soil series and had the same overall agronomic management regime. The variability in concentration for these minerals was still high with ten-fold differences for Ca and Mn found among genotypes’ seed mineral concentration, although S concentrations were less variable.

**FIGURE 2:**
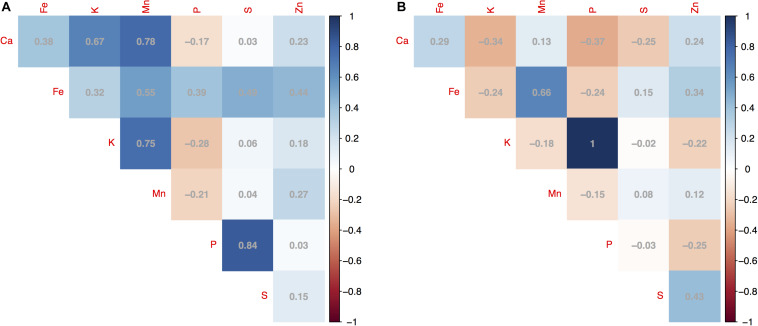
Pairwise correlation coefficients (*R*^2^) for concentrations of seven seed minerals measured for 95 mung bean (*Vigna radiata*) genotypes harvested across 2 years showing **(A)** correlations between minerals in 2015; and **(B)** between the same minerals in 2016. The magnitude of significance shown with a gradient color scale.

Pairwise correlation coefficients (*R*^2^) calculated for the seven minerals within year showed different patterns depending on the mineral. Some correlations were high as with K and P measured in 2016 (*R*^2^ = 1) or P and S (*R*^2^ = 0.84), Mn and Ca (*R*^2^ = 0.78), and Mn and K (*R*^2^ = 0.75) measured in 2015 (Figure 2A); showing relationships between uptake of the macronutrients into seeds harvested from mung beans on the soil type for this location. Meanwhile, Fe, Zn, and Mn were positively and highly correlated with each other in the same year. The correlation coefficients of Fe-Mn, Fe-Zn, and Mn-Zn measured in 2015 were 0.55, 0.44, and 0.27, compared to 0.66, 0.34, and 0.12 measured in 2016 (Figure 2B). Correlation was not observed within the seven minerals or between the same mineral measured in two different years. The lack of correlation between years may again have been due to climate variability and were not explored further.

### SNP Discovery, Principal Component Analysis, and Physical Mapping

**FIGURE 3:**
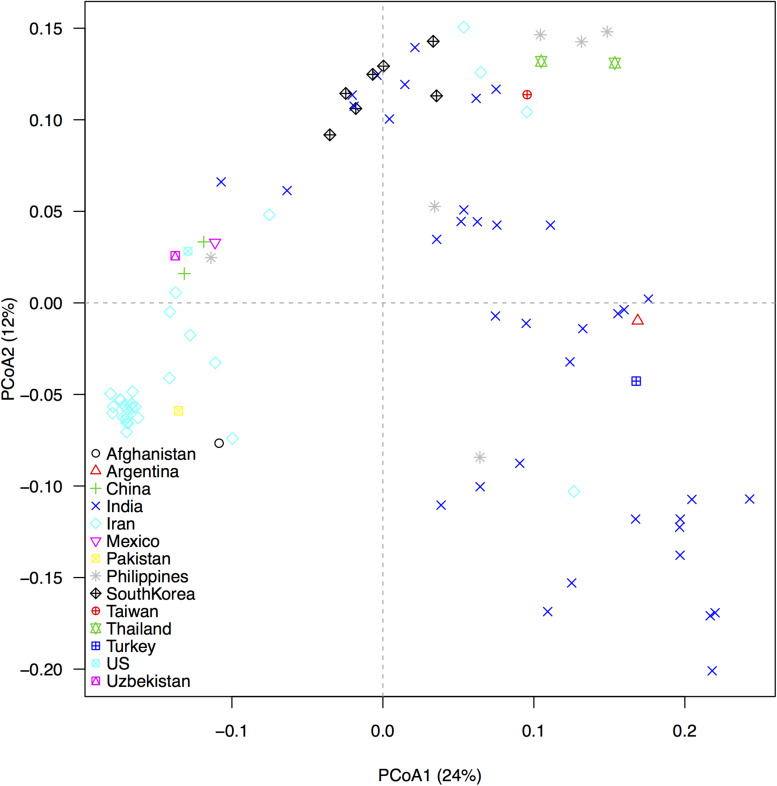
Genetic associations between 95 mung bean (*Vigna radiata*) accessions from the USDA core collection based on 6,486 SNP markers developed from Genotyping by Sequencing (GBS), as revealed by a principal coordinate analysis (PCoA). Different colored symbols identify the country of origin of each accession. The percentage of variation explained by each axis is shown within parenthesis in the label of the corresponding axes.

**FIGURE 4:**
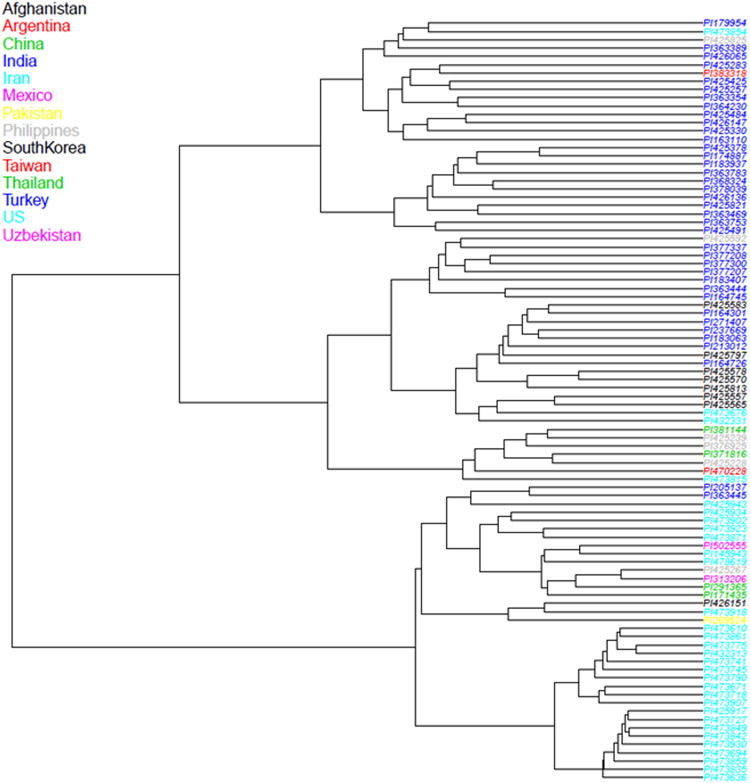
Neighbor-Joining (NJ) dendrogram showing the relationships between 95 mung bean (*Vigna radiata*) accessions from the USDA core collection based on 6,486 SNP markers developed from Genotyping by Sequencing (GBS) with color coding of branches by country and shaded by region in Asia or the Americas. The end of each branch provides the Plant Introduction (PI) number for the genotype.

In our GBS technique, tissues from small cells harvested from etiolated, mung bean sprouts were better for DNA extraction compared to expanded cells from fully grown seedlings (data not shown). We used the shoot tips of sprouts that not only contained more cells than expanded leaves, but also reduced the time and space for genetic experiment preparation. ApeK1 was found to be an ideal enzyme for GBS library preparation and after filtering, a total of 6,486 SNP markers were validated for the analyses of the mung bean accessions (Supplementary Table S1) and for the diversity assessment as represented in the PCoA diagram (Figure 3). That analysis grouped the mung bean genotypes from the GBS panel into three main clusters which were consistent with their origins; (1) the first group from India had the most diverse genetic spectrum; (2) a second group from Southeast Asia overlapped slightly with the first group; and (3) a third group consisted of less diverse mung beans were from Central (Uzbekistan), East (China and Korea), and West (Afghanistan, Iran, and Pakistan) Asia or other parts of the world. Many grouping were by country or by region as shown in the dendogram (Figure 4). For example, mung beans from Iran were grouped with accessions from Afghanistan, Pakistan and Uzbekistan. The genotypes from the Americas (Argentina, Mexico, and United States) grouped together and were all probably derived from breeding lines.

**FIGURE 5:**
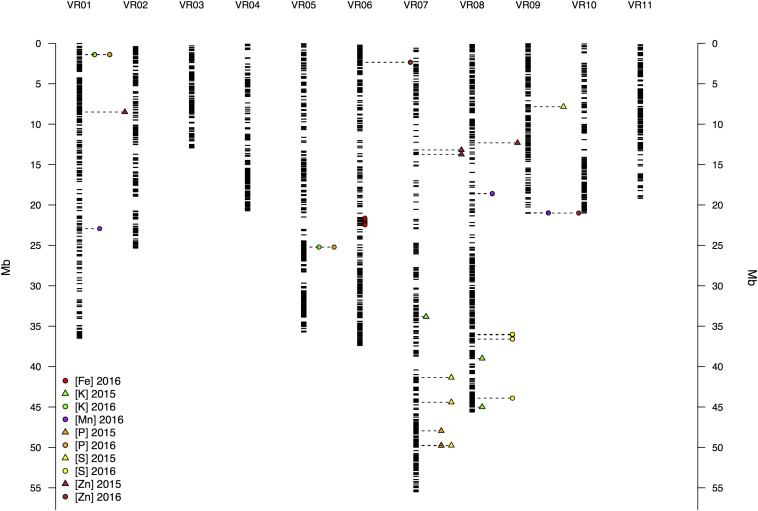
Physical map of the 6,486 SNP markers identified by Genotyping by Sequencing (GBS) of 95 mung bean (*Vigna radiata*) accessions showing all 11 mung bean (VR) chromosomes. Physical position is shown in millions of base pairs (Mb) based on genome sequence in Phytozome. Each black hyphen corresponds to a SNP marker. The other symbols indicate SNP loci significantly associated with various seed mineral concentrations in different years according to the legend and coloring indicating each element analyzed by inductively coupled plasma (ICP) spectroscopy.

**FIGURE 6:**
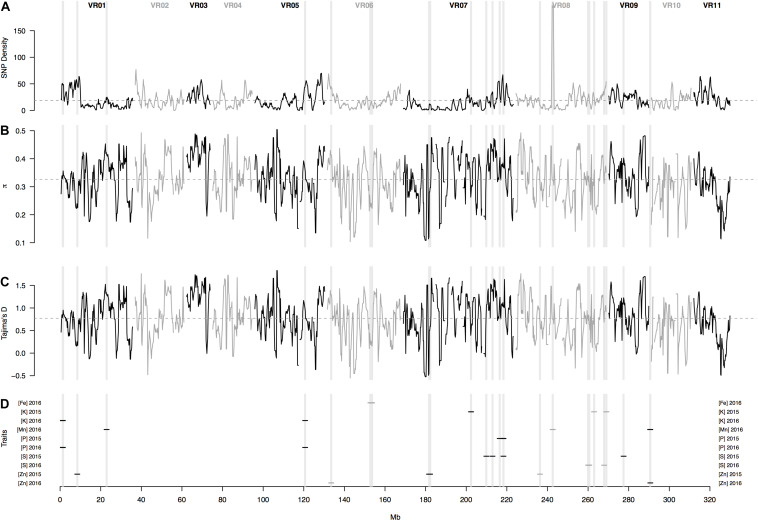
Patterns of genome-wide diversity based on 6,486 SNP markers developed from Genotyping by Sequencing (GBS) of 95 mung bean (*Vigna radiata*) accessions. A sliding window analysis (window size = 1 Mb, step size = 200 Kb) was used to compute **(A)** SNP density, **(B)** nucleotide diversity as measured by π, and **(C)** Tajima’s D. **(D)** shows the SNP loci significantly associate with various seed mineral concentrations in two different years. Results of all windowed analyses were plotted against window midpoints in millions of base pairs (Mb). Black and gray colors highlight different mung bean (VR) chromosomes. Gray dashed horizontal lines indicate genome-wide averages. Gray vertical boxes indicate the 1 Mb flanking region of each marker that was associated with a seed mineral locus.

In physical mapping of the SNP loci, the markers were well distributed across the chromosomes of mung bean and marker density did not decay toward presumed centromeres (Figure 5). Overall, average marker density was found to be 19 SNPs per Mb of sequence ranging from 1 to 200 loci. Confidence intervals at 95% probability around the mean (95% CI) for number of SNPs per Mb was at least one up to 55 (Figure 6A). Knowing that markers were found rather evenly across the entire genome, a sliding window analysis (window size = 1 × 10^6^ bp, step size = 200 kb) was conducted to understand the patterns of genome wide diversity. Average nucleotide diversity as measured by π was found to be 0.33 per Mb (95% CI, 0.17–0.46, Figure 6B). Average Tajima’s D was 0.77 per Mb (95% CI, -0.17–1.59, Figure 6C).

### Marker x Trait Associations (MTAs) per Mineral

**FIGURE 7:**
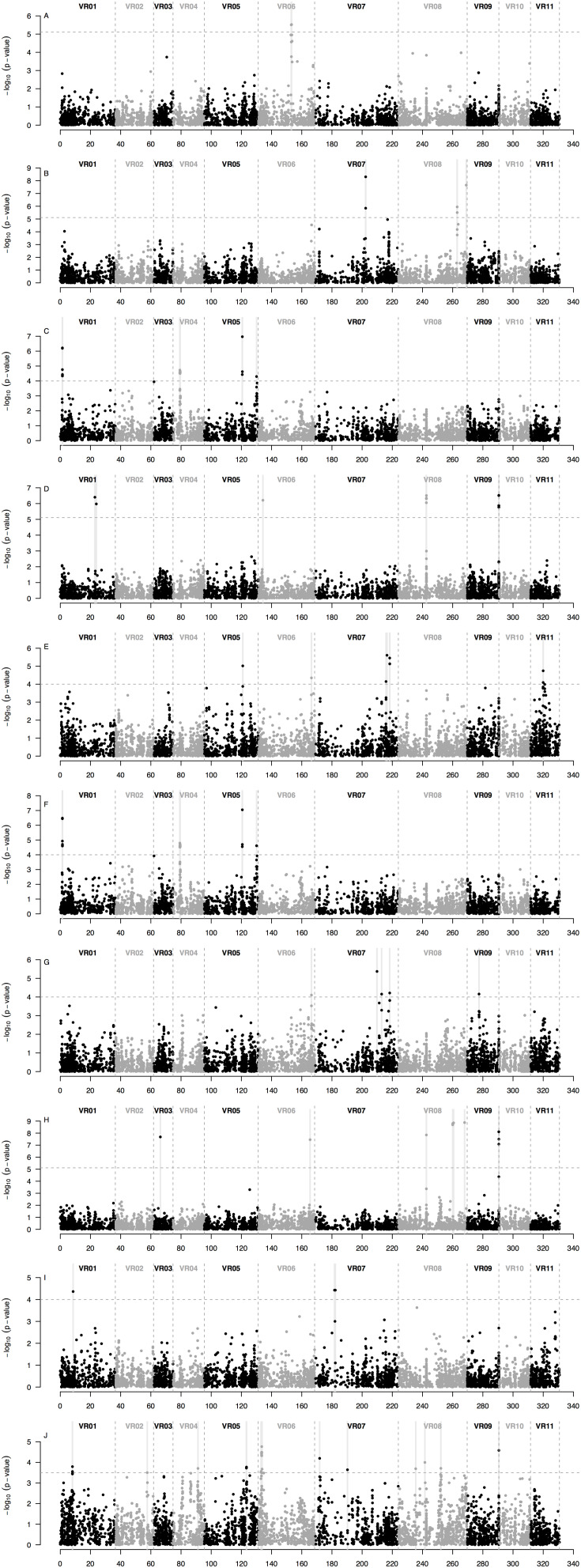
Manhattan plots of the SNP loci that were significantly associated with seed mineral concentrations in 95 accessions of mung bean (*Vigna radiata*) measured in two different years across a total of 6,486 loci from genotyping by sequencing (GBS) ordered according to physical map position along the 11 mung bean (VR) chromosomes of the Phytozome v.1.0 genome sequence. The plots display probability (*P*)-values per-marker on a -log_10_ scale for the associations between each SNP locus in order down each chromosome and each seed mineral concentration in each year. Significant marker x trait associations (MTAs) shown for: **(A)** Fe measured in 2016; **(B)** K measured in 2015; **(C)** K measured in 2016; **(D)** Mn measured in 2016; **(E)** P measured in 2015; **(F)** P measured in 2016; **(G)** S measured in 2015; **(H)** S measured in 2016; **(I)** Zn measured in 2015; and **(J)** Zn measured in 2016. The gray dashed horizontal line marks the P-value threshold after Bonferroni-correction for multiple tests. Black and gray colors highlight different mung bean (VR) chromosomes. Gray vertical boxes indicate the 1 Mb flanking region of each marker that was associated. Optimum models include: **(A,G)** a general linear model (GLM) with the country of origin and the first two PCoA axes scores (from Figure 3) as covariates; **(B,E,I)** a GLM with the first two PCoA axes scores (from Figure 3) as covariates; **(C,F,J)** a GLM with the country of origin as covariate; **(H)** a mixed linear model (MLM) with a centered IBS kinship matrix as a random effect, and the country of origin and the first two PCoA axes scores (from Figure 3) as covariates; and **(D)** a MLM with a centered IBS kinship matrix as a random effect and the first two PCoA axes scores (from Figure 3) as covariates. Cases for which there were no significant associations between SNP loci and seed mineral concentrations in either year (as for Ca measured in 2015 and 2016, Fe measured in 2015 and Mn measured in 2015) are not depicted but are shown in Supplementary Figures 1–7.

Based on the GWAS analysis for each of the seven minerals, a total of 43 SNP markers were found to be highly associated in MTA analysis with seed mineral concentrations measured in either of two different years (Figure 6 and Table 2). These MTA loci were found in 20 different 1 Mb regions (Figure 6D) and explained on average 22% of the overall variation (Supplementary Table S2). It is worth noting the comparative value of two association models used, one being a general linear model (GLM) and the other a mixed linear model (MLM). In each case, the GWAS results were optimized by considering population structure and germplasm origin by country as fixed effect covariates with kinship additionally used also as a random effect in the MLM analysis. In the GLM analysis, significant associations were observed for K (Figure 7B), P (Figure 7E), S (Figure 7G), and Zn (Figure 7I) measured in 2015, and K (Figure 7C), P (Figure 7F), Fe (Figure 7A), and Zn (Figure 7J) measured in 2016. In MLM, associations were only detected for S (Figure 7H) and Mn (Figure 7D) measured in 2016.

**TABLE 2:** Summary statistics for the 43 SNP markers associated with seven seed mineral nutrients measured in 92 mung bean accessions harvested across 2 years.

**Nutrient^a^**	**Year**	**Number of associated SNPs**	**Average –log_10_ (*P*-value)**	**Number of 1 Mb regions with associated markers**	**Average *R*^2^ (%)**	**Number of 1 Mb regions with more than one associated SNP**	**Number of 1 Mb regions that explained on average more than 10% of the variation**
Fe	2016	5	5.11	1	13.4	1	1
K	2016	5	7.08	3	30.4	2	3
K	2016	4	6.04	2	27.2	1	2
Mn	2016	5	6.46	3	38.7	2	3
P	2015	4	5.33	2	22.9	1	2
P	2016	4	6.23	2	27.9	1	2
S	2015	4	4.48	4	19.0	0	4
S	2016	5	8.81	2	1.0	1	0
Zn	2015	4	4.21	3	19.2	1	3
Zn	2016	3	4.61	2	21.8	1	2
Total	NA	43	5.84	24	22.0	11	22

*Results are from the optimum genome-wide association analyses and regions are defined by 1 Mb sequences that flanked associated markers. ^*a*^Cases for which there were no associated markers (Ca measured in 2016 and 2017, Fe measured in 2016 and Mn measured in 2016) are not in this table but are found described in Supplementary Figures S1, S2, S4.*

The MTAs could be divided into those pertinent to seed macronutrients and those for seed micronutrients. In terms of the first category, 5 MTA loci in three regions were associated with K concentration in 2015, one on chromosome Vr07 (with 2 positive SNPs) and two on chromosome Vr08 (with 1 and 2 SNPs, respectively), explaining on average 30% of the variation. However, four different SNP markers over two other regions, one on chromosome Vr01 (with 3 SNPs) and the other on chromosome Vr05 (with 1 SNP), were associated with K concentration measured in 2016, explaining on average 27% of the variation. The same four SNP markers and chromosomal regions were also associated with seed P measured in 2016, agreeing with the high correlation between K and P measured that year.

On the other hand, 4 different MTA loci were located in two other genomic regions, both on chromosome Vr07 (with 1 and 3 SNPs, respectively), were associated with seed P concentration measured in the previous year, 2015, explaining on average 23% of the variation. In comparison, 3 MTA loci in only two regions, one on chromosome Vr06 (with 2 SNPs) and the other in chromosome Vr09 (with 1 SNP), were associated with seed P measured in 2016, explaining on average 22% of the variation.

In terms of one of the most important micronutrients for biofortification, 5 SNP markers were associated with seed Fe measured in 2016. These were in a single region in chromosome Vr06 and explained on average 13% of the variation. Interestingly, this MTA region coincided with values of lower nucleotide diversity (π) and Tajima’s D profiles (Figures 6B,C), suggesting a selective sweep in this region given that the genomic average already accounted for demographic peculiarities such as bottlenecks according to previously used criteria (Cortés et al., 2012a, b; Blair et al., 2016a).

For seed Mn concentration in 2016, 5 SNP markers were associated with three genomic regions, one MTA on chromosome I (with 1 SNP), another MTA on chromosome Vr08 and a further MTA on chromosome Vr09 (both with 2 SNPs each) explaining on average 39% of the variation. Interestingly, the associated region for seed Mn concentration on chromosome Vr08 coincided with the peak in the SNP density profile (Figure 6D) of 200 SNPs/Mb with these extreme cases of high diversity usually matching genomic features such as inversions. Most other regions contained less than 60 SNPs per Mb and averaged near 20 SNPs per Mb, in other words tenfold less than for this region.

For seed S concentration as measured in 2015, there were associations with 4 additional SNP markers found in four different regions, three on chromosome Vr07 and one on chromosome Vr09, explaining on average 19% of the variation. In addition, five different SNP markers were associated with S concentration in 2016. These were found in two other regions, both on chromosome Vr08 (with 4 and 1 SNPs, respectively), but only explained very low percent the variation despite having LOD values above 8.5. Four of the five associated markers fell within the same 1 Mb region on Vr08, suggesting that the explanatory power in this region emerges from various SNPs together rather than single marker associations.

Finally, seed Zn concentration in 2015 was associated with 4 markers over three regions, one on chromosome Vr01 (with 1 SNP), one on chromosome Vr07 (with 2 SNPs) and the other one on chromosome Vr08 (with 1 SNP), and explained on average 19% of the variation. One further associated region at the end of chromosome Vr09, though not involving the same associated markers, was associated with both Mn and Zn measured in 2016 (explaining on average 39 and 19% of the variation, respectively).

Overlap of significant SNP for different mineral concentrations was observed for all 4 markers that were associated with both K and P measured in 2016 (explaining on average 27% of the variation), 1 marker at the end of chromosome Vr07 was associated with both P and S measured in 2015 (explaining 23 and 18% of the variation, respectively) and associated with high correlation for these two elements in that year (*R*^2^ = 0.84, Figure 2A). On the other hand, the optimum models for Ca measured in both years (Supplementary Figure S1), Fe measured in 2015 (Supplementary Figure S2), and Mn measured in 2015 (Supplementary Figure S4) did not have any significant SNP associations and therefore were not reported on in the sections above. This might be because of the alluvial nature and slightly acid to neutral pH of the river valley soil used and might not extrapolate to other locations or soils but is fairly typical of production zones for legumes in Tennessee.

### Candidate Gene Identification

A total of 35 regions were identified within the windows of 43 significant SNP loci associated with the different mineral concentrations. Of these, 11 regions were associated with seed macronutrients, 12 with micronutrients and 12 with other elements (Supplementary Table S2). Totals of 10, 9, and 7 genes were found for each category. For macronutrients, these included three genes on chromosome Vr01 (*Vradi01g00820*, *Vradi01g00830*, and *Vradi01g00840*) and one on Vr05 (*Vradi05g16350*) for K and P concentrations measured in 2016, respectively. Two genes (*Vradi07g26320* and *Vradi07g26340*), both on chromosome Vr07, were near SNPs associated with P concentration in 2015. Three genes (*Vradi07g14180* on chromosome Vr07, and Vradi*08g22740* and *Vradi08g17100* on chromosome Vr08), were identified as associated with K in 2015. These genes that identified with macronutrients were involved in cell proliferation regulation, oxidation-reduction process, ATP biosynthesis pathways, vacuolar transporting pathway, and transcription and translation regulation during plant development. Two genes, *Vradi05g16350* and *Vradi08g15760*, were uncharacterized genes with unknown protein function.

For the micronutrients, a total of 12 regions were found to be associated with Fe and Zn, five with the former and seven with the latter. Genes related to Fe accumulation were all located on chromosome Vr06 and participate in various biological process such as metal iron binding (*Vradi06g09900*), metal translocation (*Vradi06g10020*), uptake of minerals in plants (*Vradi06g10060*), membrane transport (*Vradi06g10120*) and ATP binding activities (*Vradi06g10210*). Meanwhile, of the four genes identified for Zn measured in 2015, some were found on chromosome Vr01 (*Vradi01g05570*) and others on chromosome Vr07 (*Vradi07g05950* and *Vradi07g06200*).

These four genes were involved in gene regulation (*Vradi01g05570*), metal translocation (*Vradi07g05950*), metal iron binding (*Vradi07g06200*), and membrane signal transduction pathways (*Vradi06g02380*). One additional gene (*Vradi06g02380*) was identified on chromosome Vr06 that was associated with Zn in 2016; while more were identified for S over two years. S concentration might reflect the accumulation of sulfur containing amino acids such as cysteine and methionine, another nutritionally important trait for legumes. Therefore, we were interested in the loci associated with this mineral even if they explained moderate and low average variability (*R*^2^) in 2015 and 2016, respectively. A total of six genes (*Vradi07g21720*, *Vradi09g05410*, *Vradi07g19370*, *Vradi08g16110*, *Vradi08g15850*, and *Vradi08g15760*) with three each per year were identified. As is evident from the gene numbering that reflects the chromosome base position, the last of these three genes, all on Vr08 were very close together in position and this might explain the lower *R*^2^ values as the genes might have been conferring a string association for the region as a whole more so than for each marker individually. One of the genes had unknown function while the other two had cell functions. Finally, a total of four SNPs was associated with Mn measured in either of the two years but only one gene (*Vradi01g11650*) was found in their vicinity. This gene was involved in a carbohydrate metabolic process unrelated to Mn accumulation.

## Discussion

The variability found for seed mineral concentration in mung bean agreed with that found in most legumes, especially for the better studied micronutrients, Fe and Zn which have been the subject of biofortification breeding (Dwivedi et al., 2012). Our results showed averages between 30 and 40 ppm of seed Fe and wide variation for individual genotypes in mung beans, which agrees with results for other small seeded legumes such as Mesoamerican common beans (Blair et al., 2013). A narrower range for values and averages between 25 and 30 ppm seed Zn is also typical of small seeded legumes (Gelin et al., 2007; Blair et al., 2009) but we found mung bean to have higher average Zn values, perhaps due to selection rice-based, vegetarian diets.

Large seeded legumes such as Andean common beans or Chickpeas tend to have more Fe but about the same Zn perhaps because of the influence of embryo size differences and the proportion of seed coat to cotyledonary tissues (Ariza-Nieto et al., 2007; Cvitanich et al., 2010). It seems that mung bean is intermediate between these extremes represented by large and small seeded legumes. The distinction is important as small seeded legumes such as mung bean, cowpea and rice bean, all *Vigna* species, are preferred in the diets of Africa and Asia due to their quick cooking time and ease of de-hulling or other processing, compared to large seeded legumes found in European diets and in areas with more fuel sources used for longer cooking times.

Differences in seed macro-nutrient concentrations for Ca, K, and P were significant but variable from year to year and site to site as was Mn. P uptake efficiency is known to vary between legume accessions based on P use efficiency and microbial interactions (Ramaekers et al., 2010; Lynch, 2011). The study of Ca, K, and Mn in legumes is less exhaustive but does show that soil pH is important for uptake. Since we used a neutral soil but relied on previous fertilization, we may have had an effect of field variability. However the field was all part of a rotation of corn/beans.

A second point of our study was that SNP discovery was highly effective by GBS and was useful in distinguishing the mung bean genotypes from the USDA core collection. In general, population diversity analyses found country-based grouping in accordance with isolation by distance model, well-described for self-pollinating legumes such as common bean (Cortés et al., 2012a). In physical mapping of the SNPs, markers were well distributed across the chromosomes of mung bean and marker density did not decay toward presumed centromeres in contrast to what has been observed for other species (Ellegren and Galtier, 2016).

Indeed, GBS has been shown to be an effective method that allows simultaneous discovery and genotyping of a large number of novel SNPs in a lot of species (Davey et al., 2011) as first suggested when the method was conceived and tested (Elshire et al., 2011). The first GBS study in mung bean was conducted by Schafleitner et al. (2016) with a total of 9,289 and 6,463 SNPs mapped in a mung bean genetic map in two RIL populations for bruchid resistance. A year later, an additional 1,321 SNPs were incorporated into 11 linkage groups for mapping of flowering genes using GBS in mung bean (Hwang et al., 2017). Now that the whole genome sequence is available for mung bean (Kang et al., 2014), an added benefit of GBS evaluation is the physical mapping of all the SNPs found.

For GBS to be commonplace in the legumes proper DNA extraction methods are needed. Regular CTAB methods with tissue from fully grown plants (Doyle and Doyle, 1987) usually take 4–6 weeks in legumes which during early growth stages contain many bioactive phytochemicals and an abundance of starch and protein, that remain a major obstacle for high quality genomic DNA isolation, especially in *Vigna* species. In this study, we developed a sprout-based DNA extraction technique that provided enough high-quality DNA for GBS.

A third benefit of the large number of SNPs found in our study was the highly informative PCoA results which classified genotypes accurately based on countries and reflected diversity within the regions. The first axis explained 24% of the overall variation and mostly resolved differences between the temperate and tropical accessions. The second axis explained 12% of the overall variation and uncovered further differences between countries-of-origin as well as within-country variation. Based on both axes, the Indian accessions were the most diverse. The high diversity of mung beans from India is consistent with the hypothesis of domestication there or in an adjacent region but with additional diversity in Eastern, Southwestern and Southeast Asia.

Moderate diversity of mung beans in other countries can be explained by it being mostly selected for vegetable or grain and not being adjacent to wild relatives for inter-crossing. Proximity between regions could have explained the overlap between the cultivated mung bean accessions we analyzed from India and Southeast Asia. Since mung bean is one of the most important food legume crops in South, East and Southeast Asia, where 90% of global production currently occurs, historical germplasm exchange has been extensive for the crop. The unique genetic diversity of Iran mung bean accessions has been reported in other diversity studies (Islam and Blair, 2018; Noble et al., 2018), which could be due to the geographic isolation and breeding history of mung beans in West Asia, where a genetic bottleneck for dryland adaptation could have occurred.

In the fourth and most important of our results, we found that mung beans have a large amount of diversity for nutritional quality and are a tractable genetic system for the study of nutritional genomics. The social impact of this finding for biofortification of crops cannot be overemphasized as mung bean is important in the highly populous countries in Asia where a rice-based diet tends to be low in the micronutrients such as Fe and Zn provided by legumes.

Nutrient-enriched crops offer an important approach to fight nutrient deficiency known as “hidden hunger.” Legumes are important as nutrient dense crops and are often considered as poor man’s meat in many developing countries. Mung bean seeds exhibit wide variability of macro- and micro-nutrient traits in germplasm collections and inheritance was quantitative in nature governed by many genes and influenced by environment in this study. The concentration of macronutrients in mung bean seeds varied by site and year, with differences probably due to excess precipitation in year 2, suggesting the sensitivity of the genotypes to environmental variations from trial to trial. The water stress could trigger the plant response system for abiotic stress, causing the unstable accumulation of macronutrients in plant tissues.

The micronutrients, Fe, Mn, and Zn, are three of the essential minerals for the growth and development of all higher organisms. In the present study, all three nutrients were positively correlated in each year. The correlation of seed Fe and Zn levels in seeds was reported in many crops and has been extensively studied in other crops such as common bean (Blair et al., 2009), rice (Sperotto et al., 2010), and wheat (Morgounov et al., 2007). The level of Fe and Zn in this study was concurrent with that of previous researchers (Nair et al., 2013, 2015) but was more variable due to the genotype × year interaction seen and perhaps due to within field soil variability. The Mn concentration appeared stable with less variation compared to other micronutrients.

Although Fe and Zn deficiencies are among the most prevalent micronutrient deficiencies in humans, Mn deficiency is less common compare to Fe and Zn, lack of Mn can also cause a serious problem and can lead to asthma and severe birth defects. The transport of Mn is believed to share an entry route with Fe, thus the transportation of Fe, Zn, and Mn overlaps in plant biological systems, leading to joint accumulation in some circumstances (Bashir et al., 2013).

Forty-three SNPs showed significant MTAs with minerals in the seeds of current genotypic panel. Of them, 26 SNPs were associated with macronutrients (K, P, and S). Four markers were found to be associated with both P and K, one marker was found to be associated with both P and S. In total, 17 SNPs were found to be associated with micronutrients Fe, Zn, and Mn. In term of SNP locations for the MTAs within the mung bean genome, macronutrient-associated SNPs were located on chromosomes Vr01, Vr05, Vr07, Vr08, and Vr09, while micronutrient-associated SNPs spanned chromosomes Vr01, Vr06, V07, V08, and Vr09.

The multigenic control of nutrient accumulation in mung bean seeds agrees with previous of Singh V. et al. (2017) showing quantitative inheritance. These authors identified 17 QTLs for Fe and Zn in a mung bean RIL population, with seven QTL on linkage groups LG 6 and LG 7 for Zn with one QTL shared with Fe. In our study, nine out of twelve SNPs associated with Fe and Zn were located on chromosome Vr06 and Vr07. Even though different methods were used to locate genomic regions/genes for Fe and Zn concentration in mung bean, the overlapping regions indicated common genomic regions that are responsible for Fe and Zn accumulation in mung bean. Correlation between minerals was observed for mung bean by Singh R. et al. (2017) and similar results observed in common bean, where the majority of QTLs for Fe and Zn accumulation were located together (Izquierdo et al., 2018). An association study of Fe, Se, and Zn in the garden pea have also indicated that loci related to Fe and Zn are clustered (Diapari et al., 2015).

Among the candidate genes found in this study of mung beans, those MTAs associated with macronutrients were involved with biological process and plant development. This would be expected since Ca, P, K, and S are essential elements for cell proliferation and differentiation. However, candidate genes for micronutrients tended to be more specific.

For seed Fe breeding in mung bean, an interesting genomic region with MTA loci on chromosome Vr06 had the most promising candidate genes for immediate selection. Of the five genes identified within the 4 Mb significant region, three were related to iron metabolism and movement and have been extensively studied. The first gene included the SNP locus SVR06_21630747 and corresponded to a zinc finger CCCH protein which is related to Fe accumulation in Arabidopsis (Le et al., 2016). The second gene associated with Fe accumulation included the SNP locus SVR06_22114556 and corresponded to a homolog of a bZIP transcription factor, which have been shown to play a role in the uptake of minerals in plants (Assuncao et al., 2010). Finally, the nearby SNP locus SVR06_21903341 corresponded to a homolog of the Yellow Stripe1-Like (*YSL*) gene, which has often been implicated in Fe uptake and transport as well as tolerance to Fe-deficient soils (Curie et al., 2001; Kobayashi and Nishizawa, 2012).

The YSL1 proteins are members of the oligopeptide transporter family and acting as a transporter of iron- and metal-nicotianamine chelates responsible of iron loading of the seeds (Jean et al., 2005). The protein was reported in several crops including rice, maize and soybean, and expressed in several plant tissues such as root and shoot tissues and are important along with Ferric Reductase genes (Waters et al., 2002). We also found a gene similar to *YLS7* on chromosome Vr07 but this one was associated with Zn accumulation. Joint association of Fe and Zn accumulation has been identified in other legumes and a functional SNP marker called “MBkSNP_38” from Islam and Blair (2018) was also discovered to be within a *YSL* gene. As this marker was validated as a KASP assay for mung bean it could be useful for mung bean nutritional breeding even if it was evaluated on a different genotype panel. Although there is no meta-QTL analysts in mung bean yet, a multi population comparison in common bean found YSL to be one of the gene families affecting iron and zinc uptake (Izquierdo et al., 2018).

Similar analyses have been done in other legumes. In *Medicago truncatula*, genes from the Zinc/Iron-regulated transporter-related protein family (*YSL3*) were identified to be associated with seed Zn accumulation (Sankaran et al., 2009). In soybean, a homolog of the *YSL7* gene was found to be related with seed Fe accumulation (Ramamurthy et al., 2014). In chickpea, a gene similar to *YSL1* was also identified to be associated with seed Fe accumulation, along with genes from ZIP and HMA families (Upadhyaya et al., 2016). The implications for breeding across the legumes are obvious. Although mung bean biofortification is still beginning, breeding programs have been shown to produce new micronutrient rich varieties (Blair et al., 2010b).

Large variations of Fe, Zn and the correlation between Fe, Zn, and Mn in mung bean accessions reported here could be useful for breeding biofortified, mung bean varieties to reduce micronutrient deficiency in developing countries. The current study provides the first association study of mineral concentrations in seeds of mung bean. Given the multiple methods of mung bean consumption and their wide popularity especially in India (Dahiya et al., 2014), seed mineral concentration should be studied in relationship to micronutrients levels in food products that can be derived from this legume. These include whole, split or dehulled mung bean dahls; sweets such as burfi and halwa; and snacks such as papad and pakore. Our research demonstrated that the genotypic variability of mung beans and its diversity for seed mineral concentration is sufficient for a biofortification program using available genetic resources in the United States. Testing of bred mung bean varieties for Fe and Zn in India found similar levels of micronutrients with older varieties but suggested that phytic acid must be considered when breeding (Dahiya et al., 2013). Wider diversity for Fe and Zn is found when considering other release varieties from South Asia (Nair et al., 2015). In conclusion, larger numbers of genotypes, multi-site testing, utilization of landraces and consideration of phytate levels must be part of future studies.

## Conclusion

The major achievements of our study were finding variability for micronutrient and macronutrient traits and a total of 6,484 high-quality SNPs by GBS of 95 mung bean accessions. The SNP loci could be placed on all eleven chromosomes throughout the genome and the overall cost was economical especially as the modified extraction protocol provided enough quality DNA for sequencing. Given the benefits of using GBS in our study, we expect this technology to become widely adopted in mung bean research for marker discovery and application in marker-assisted breeding. Association of genotypic and phenotypic data in our GWAS models and based on our GBS dataset, identified a total of 43 SNP markers that were found to be highly associated with mineral concentration. These SNP loci were found in 20 different 1 Mb regions and explained on average 22% of the overall variation. Further variation could be explained in the future, by additional genotype x environment tests needed both locally and in other climates to evaluate the stability of the GWAS associations found. For biofortification one of the most important associations was for one SNP related to the *YSL1* gene that has been responsible for iron transportation to seeds in other plants. This SNP would show potential to be used in marker-assisted selection (MAS) for higher Fe concentration in mung bean breeding programs. This would be important for South and Southeast Asian diets which are mostly cereal and legume based, and where mung bean can be a very important food for the balanced nutrition of people in these regions. The genotyping by sequencing (GBS) technology is highly efficient for producing large numbers of SNP markers across the genome. Together with the available whole genome information of mung bean, the new loci discovered could greatly facilitate genome-wide association studies in this crop.

## Data Availability Statement

The datasets generated for this study can be found in Dryad repository https://doi.org/10.5061/dryad.k0p2ngf5s.

## Author Contributions

MB, XW, and AI planned the study. XW, AI, and LM did field harvesting and laboratory work. AI, NL, JM, and XW analyzed the seed mineral concentrations. XW, AC, and AI organized the data and prepared graphs.

## Conflict of Interest

The authors declare that the research was conducted in the absence of any commercial or financial relationships that could be construed as a potential conflict of interest.
