# Supplementary figures and images for: Genome-Wide SNP Identification and Association Mapping for Seed Mineral Concentration in Mung Bean (Vigna radiata L.)
Source: Front Genet. 2020 Jun 24;11:656. doi: 10.3389/fgene.2020.00656 (PMC7327122; doi:10.3389/fgene.2020.00656)

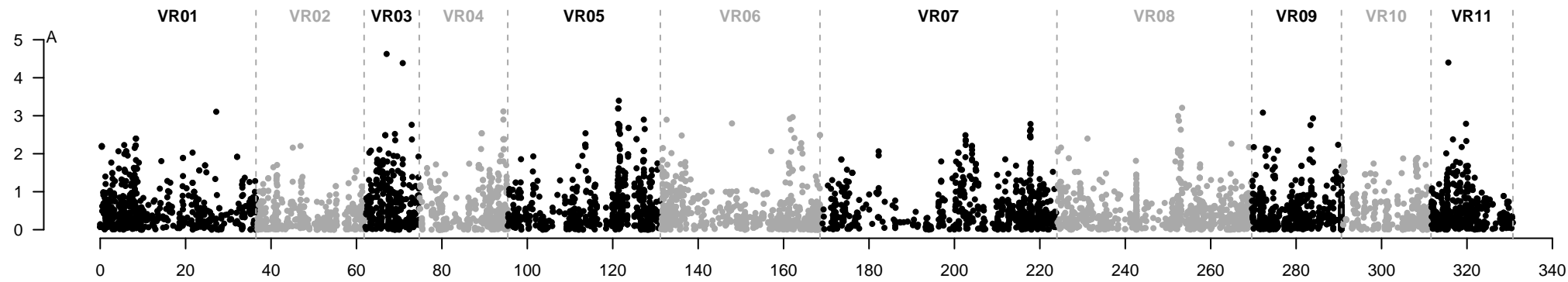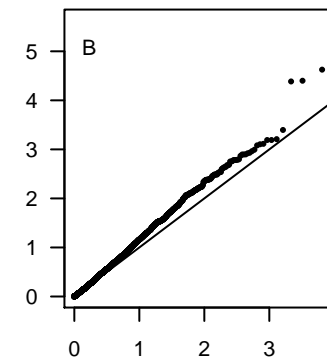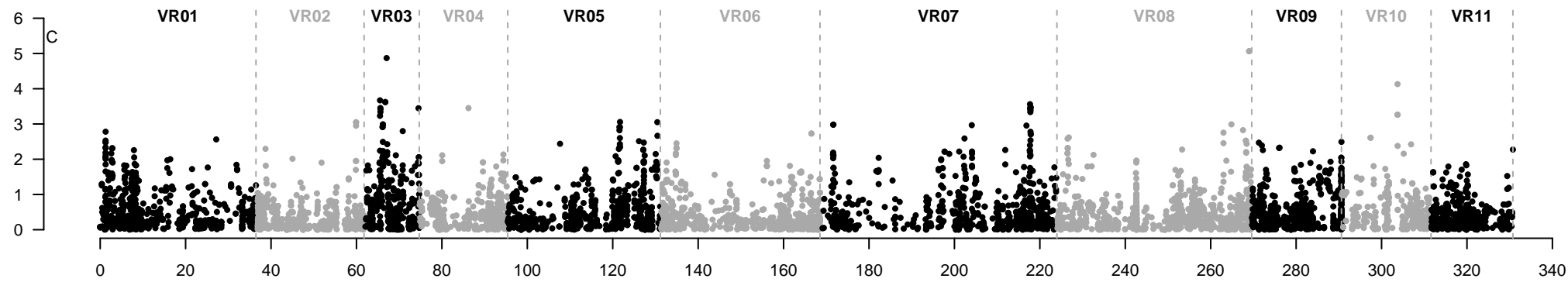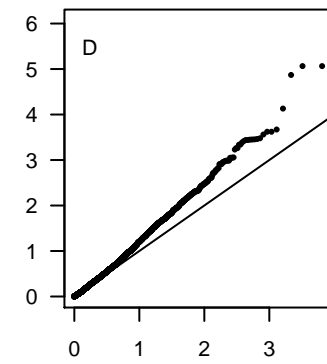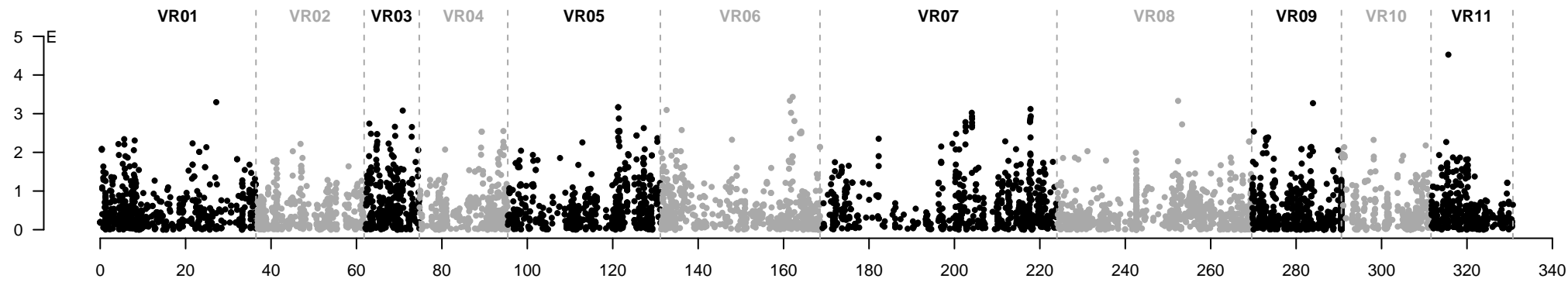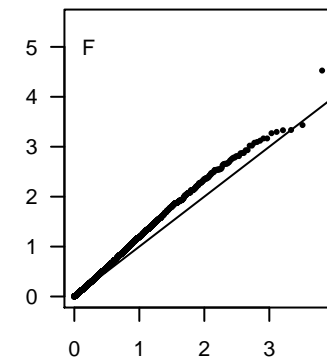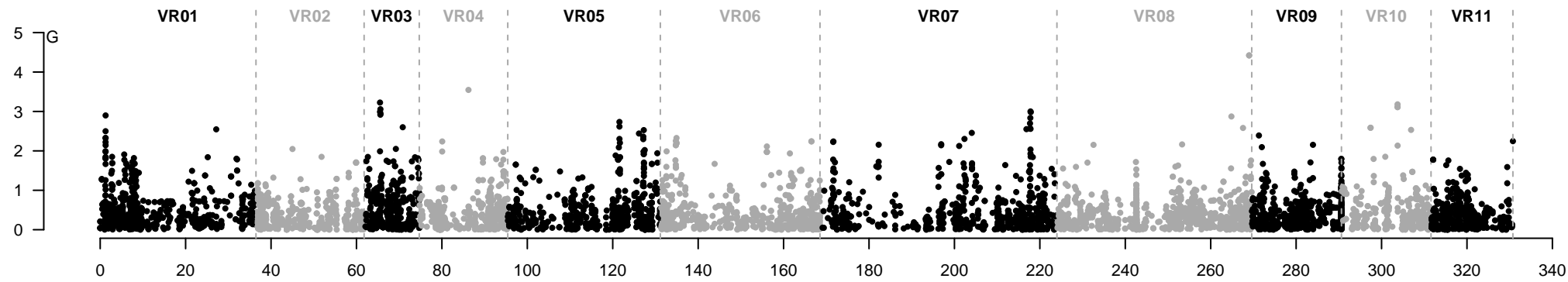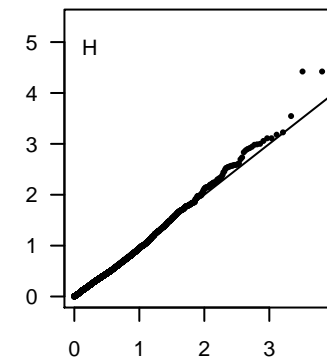

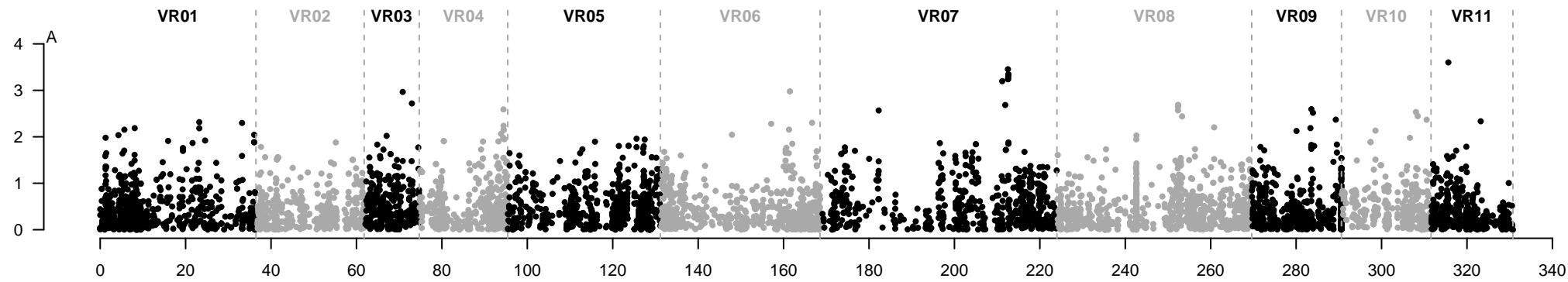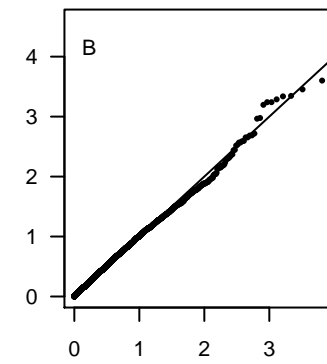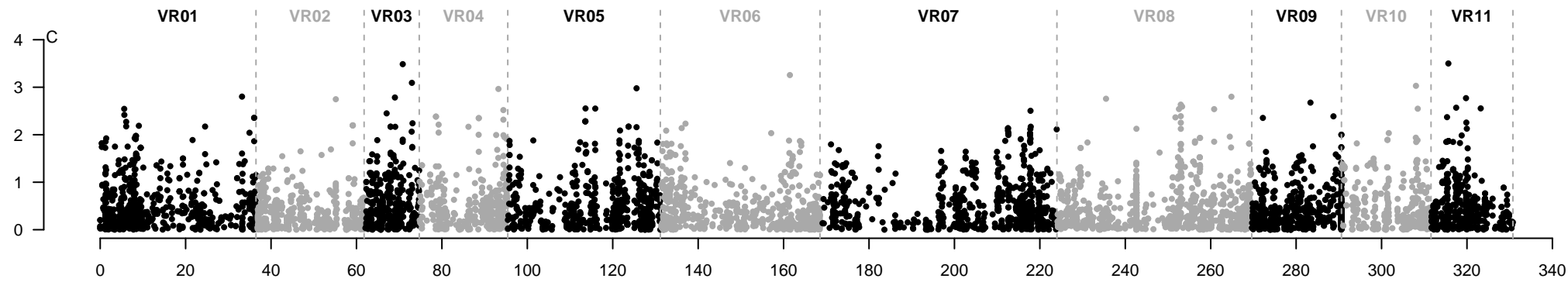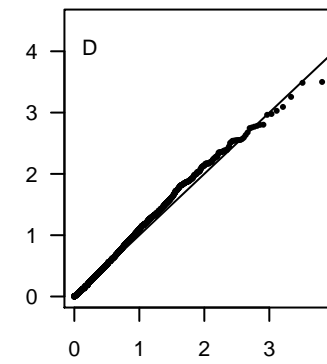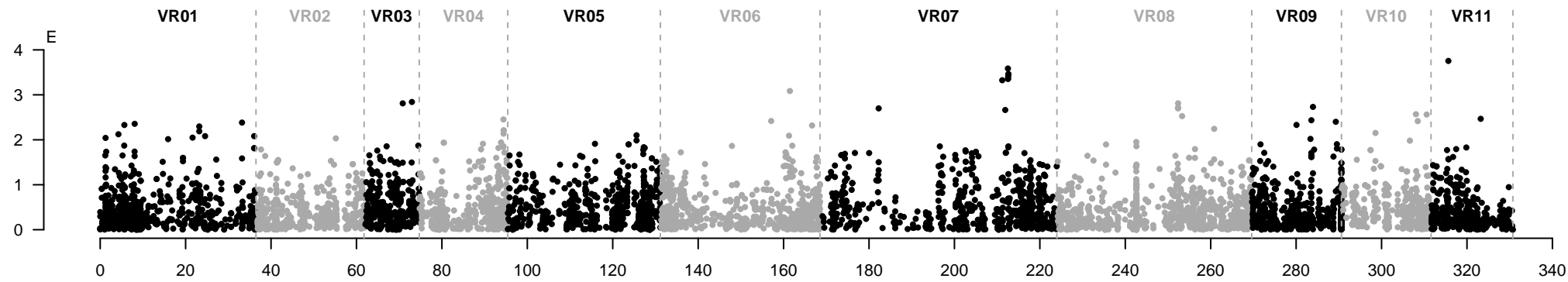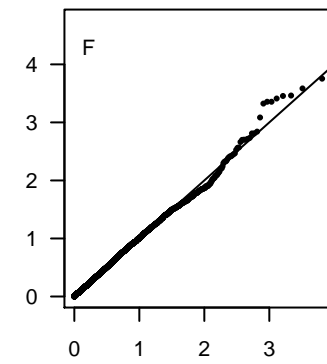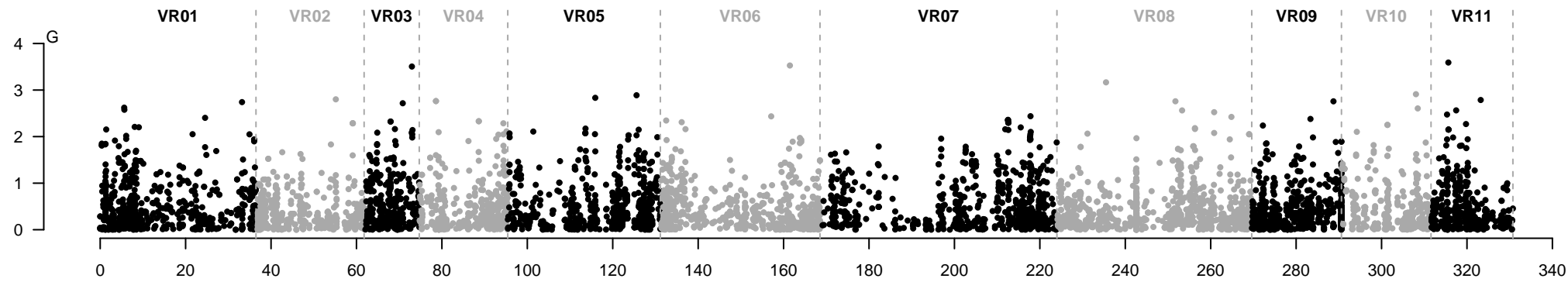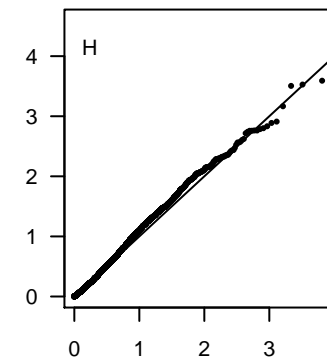

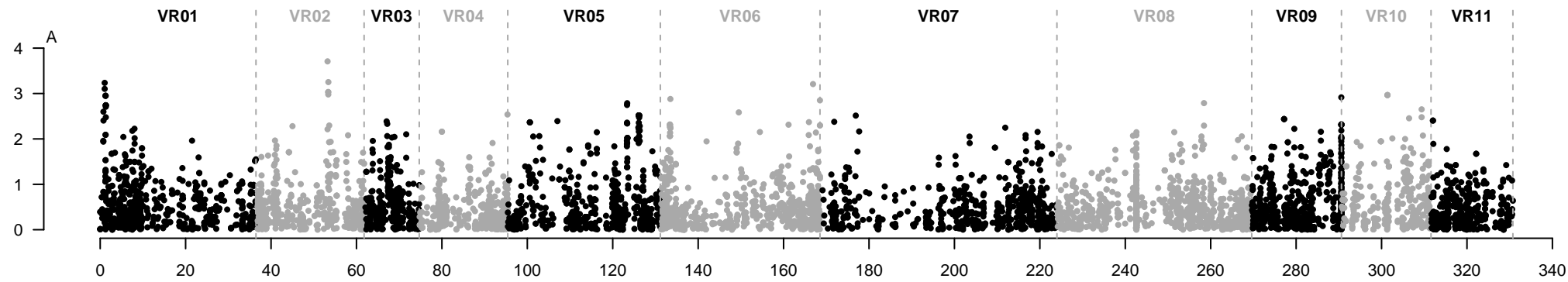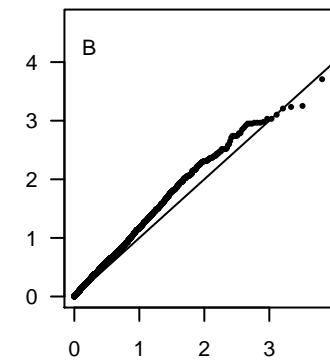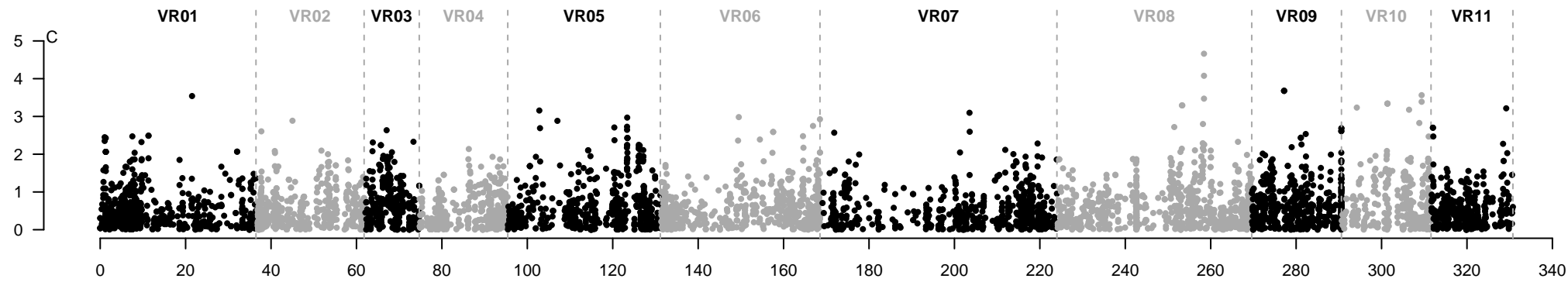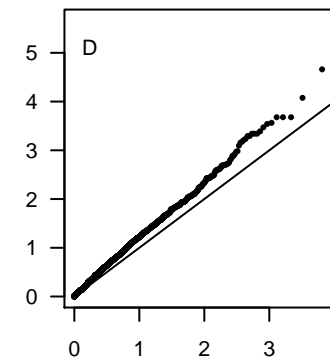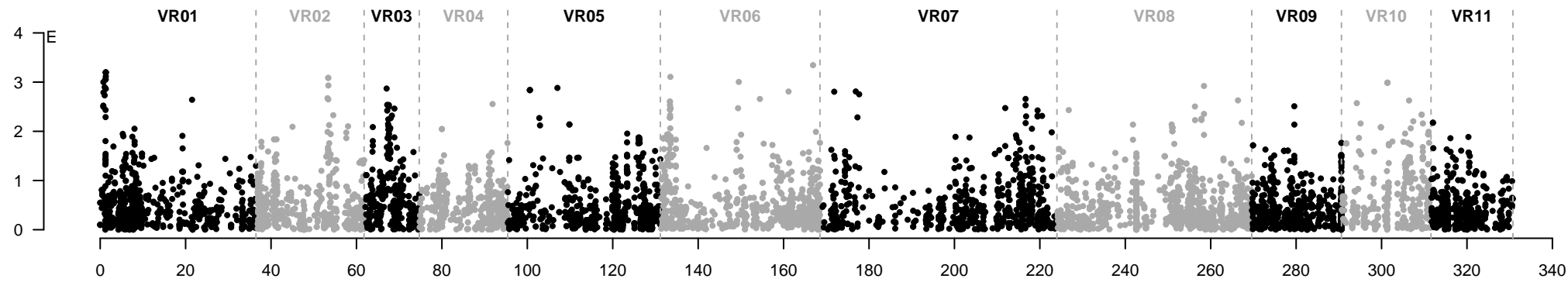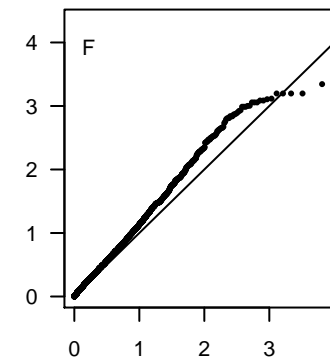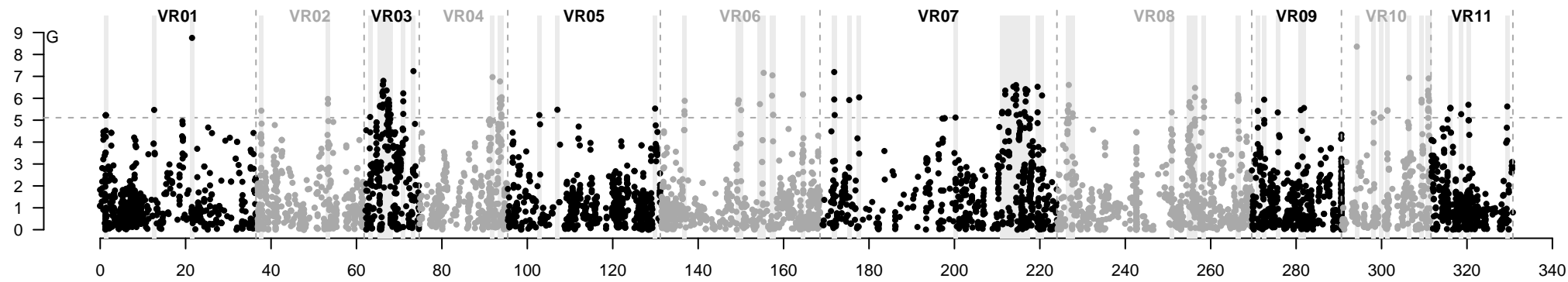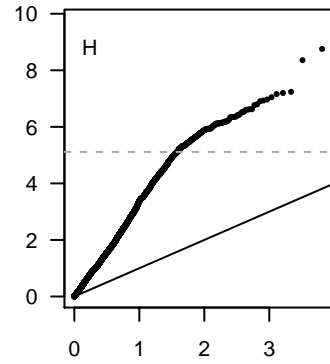

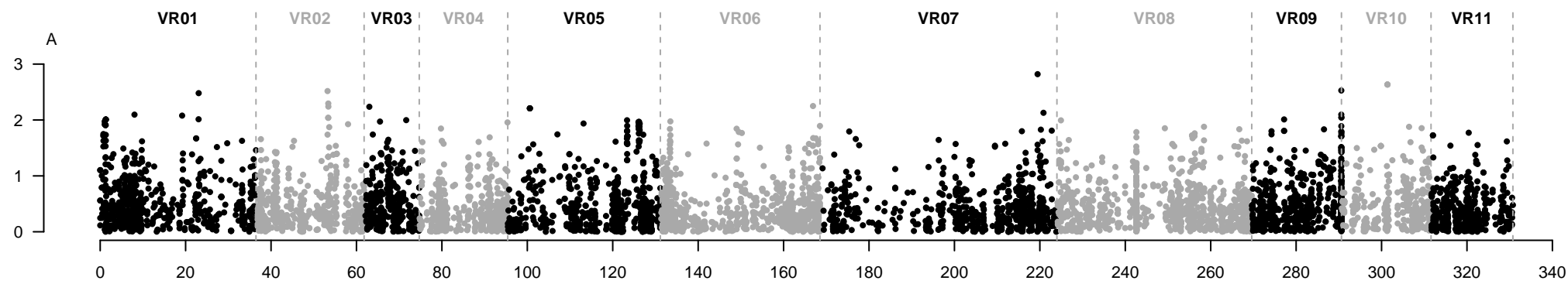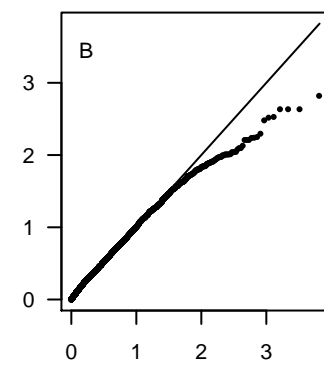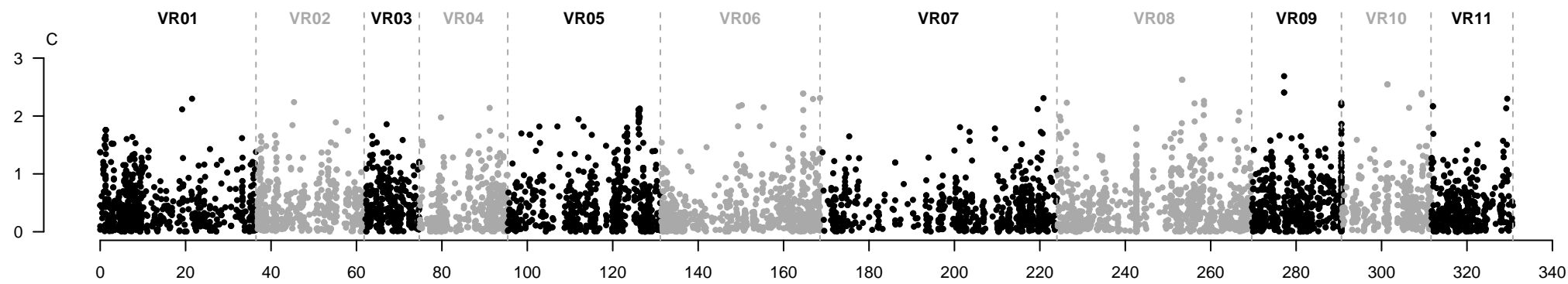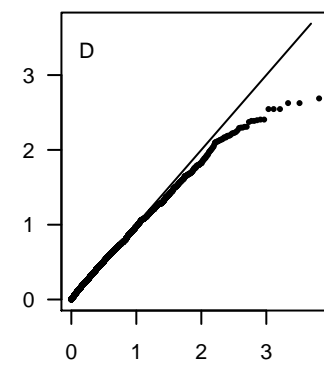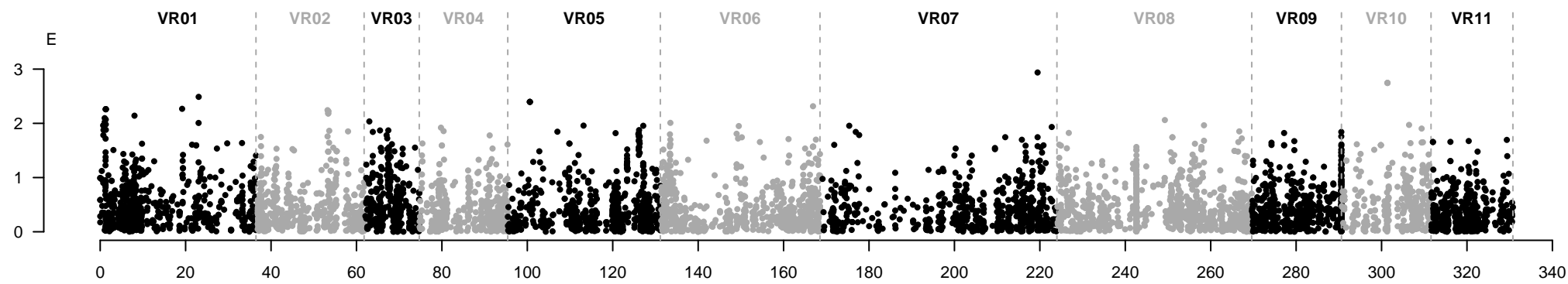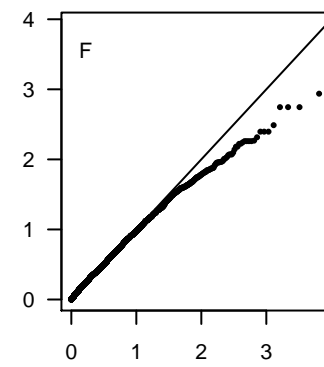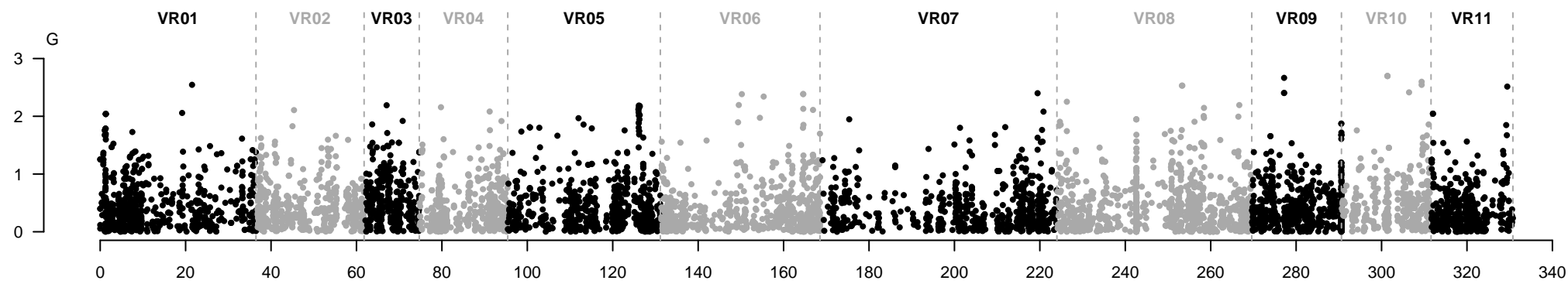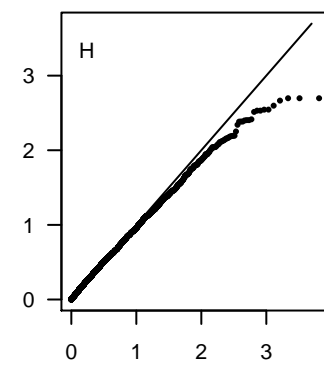

Supplement: Supplementary file 1 [file Data_Sheet_1.PDF]

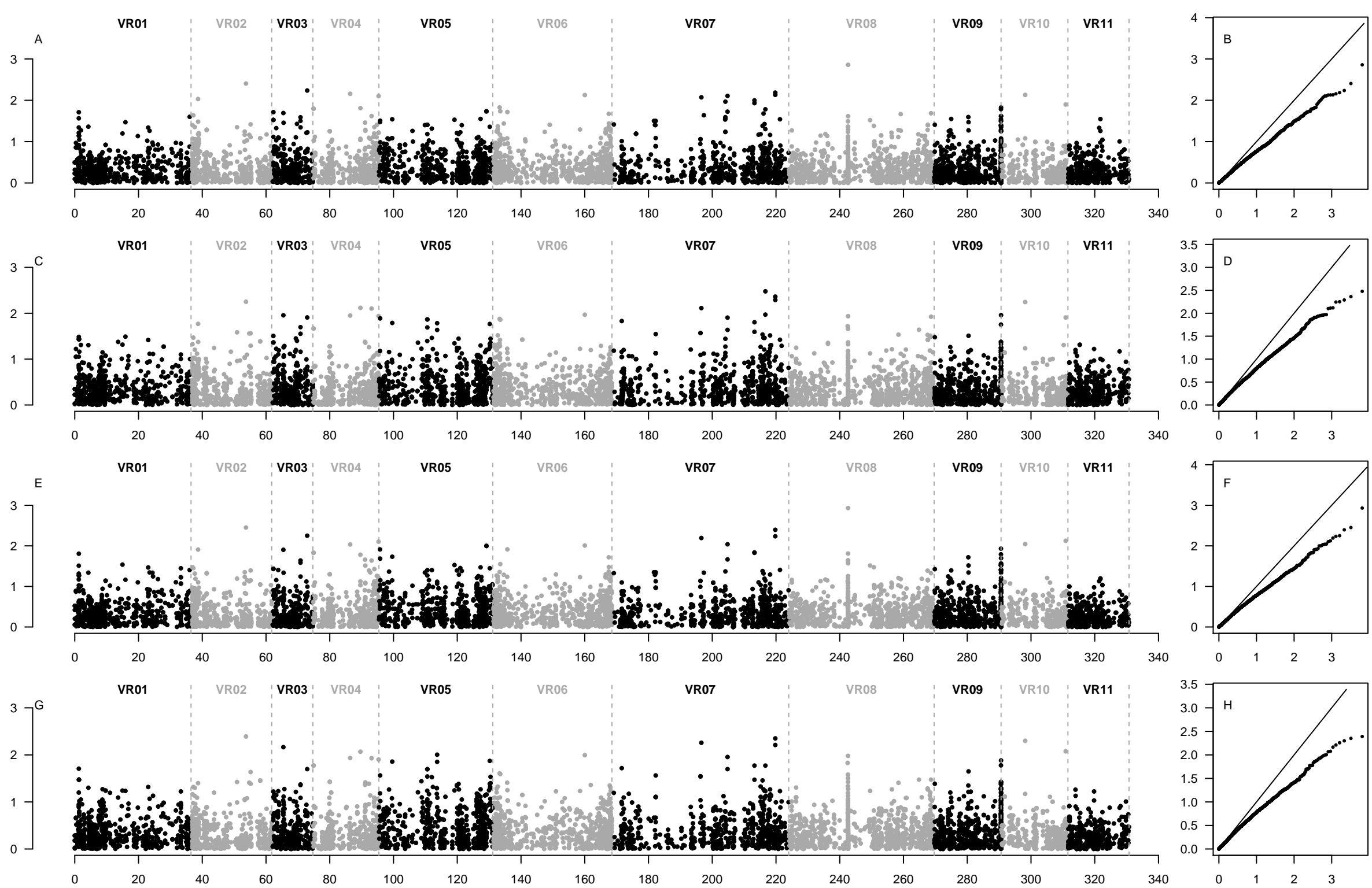

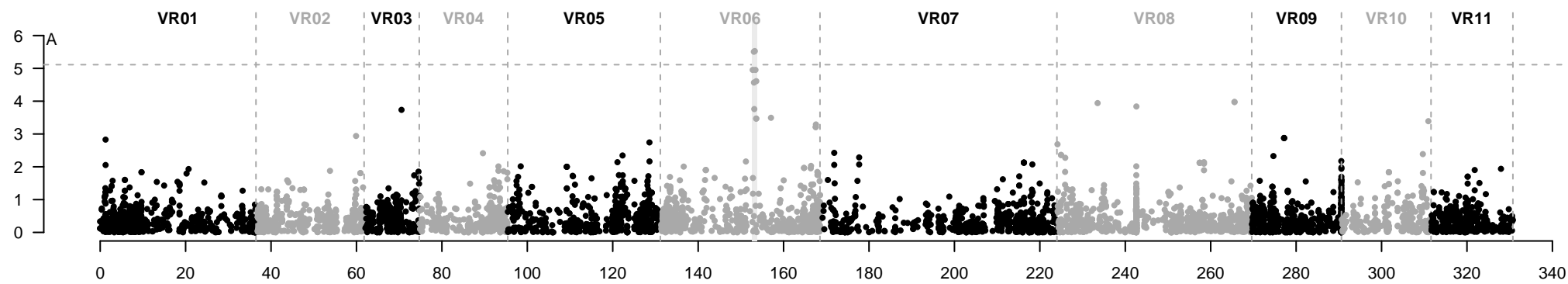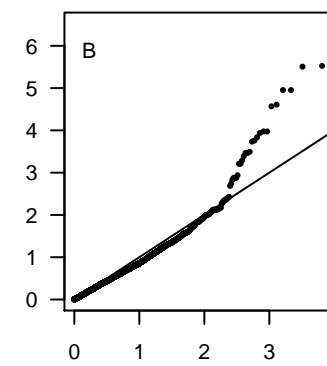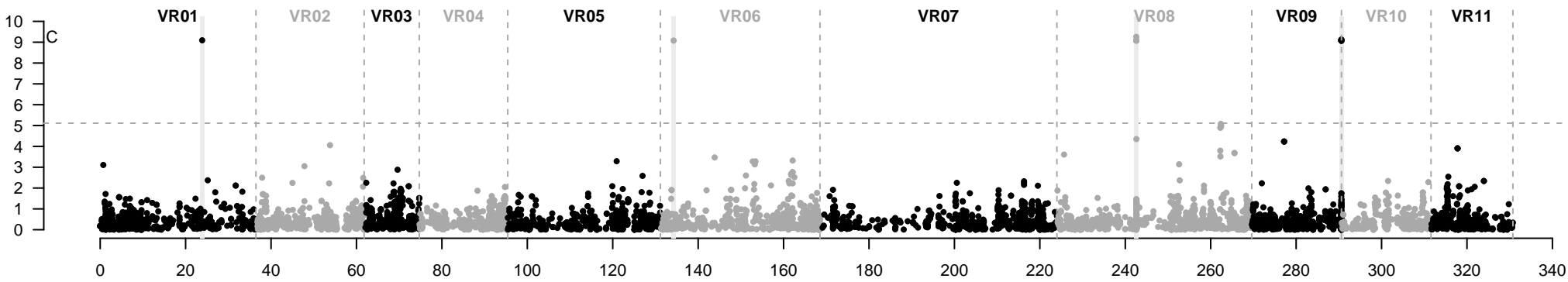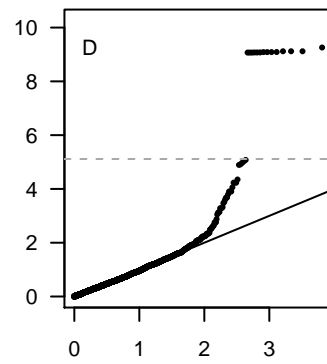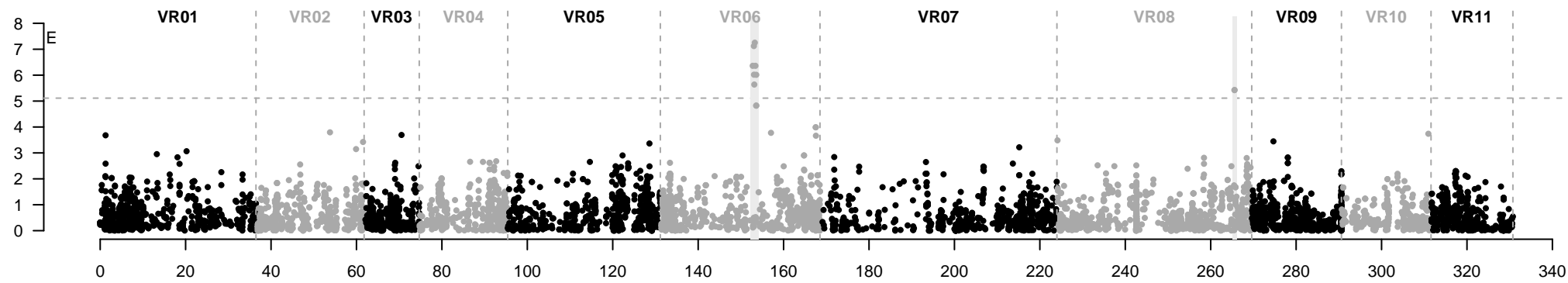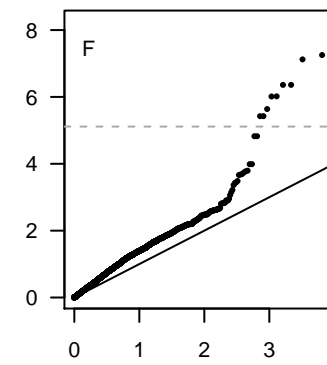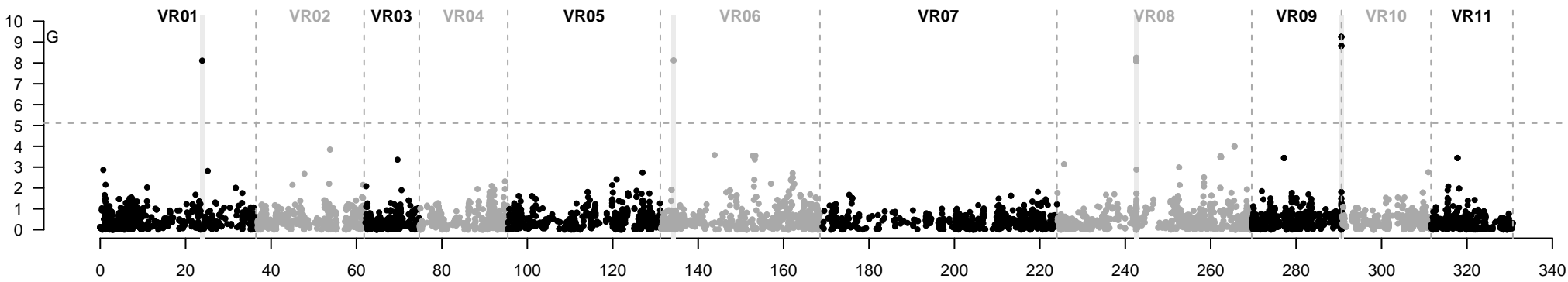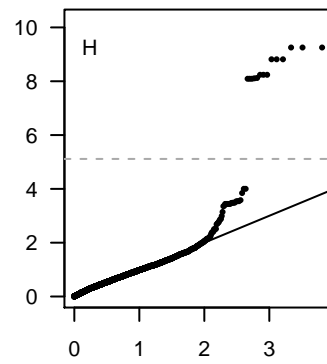

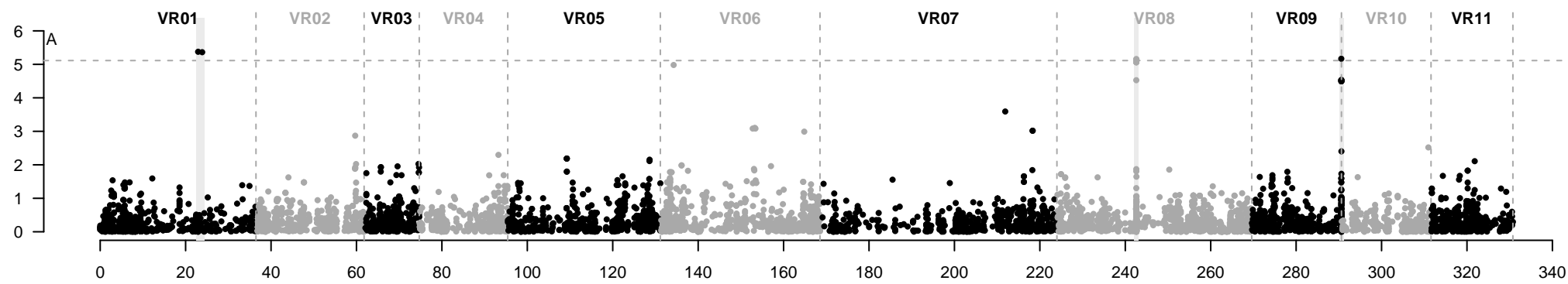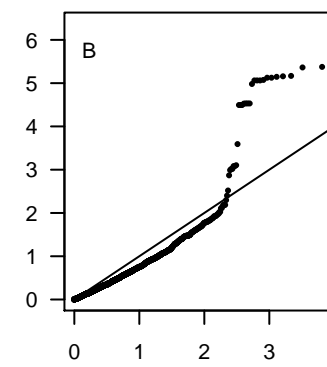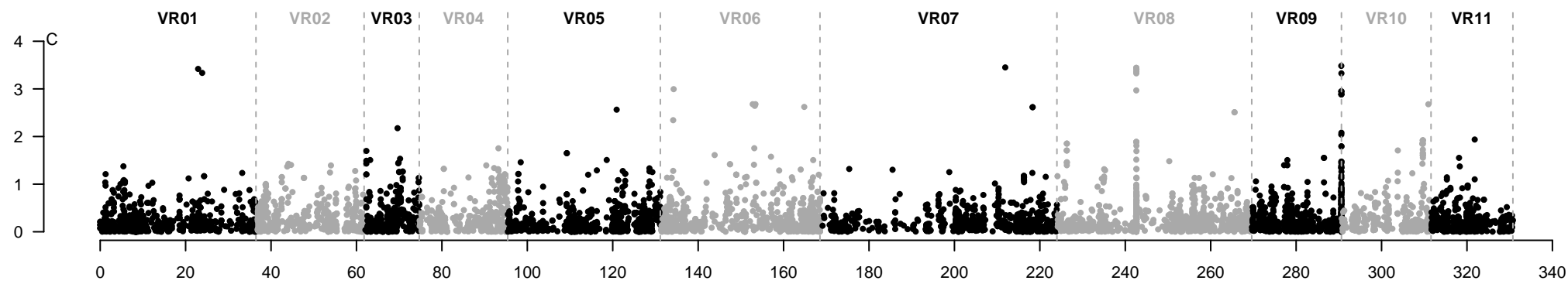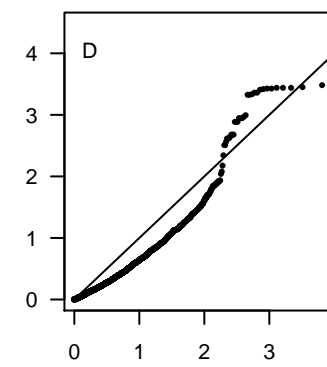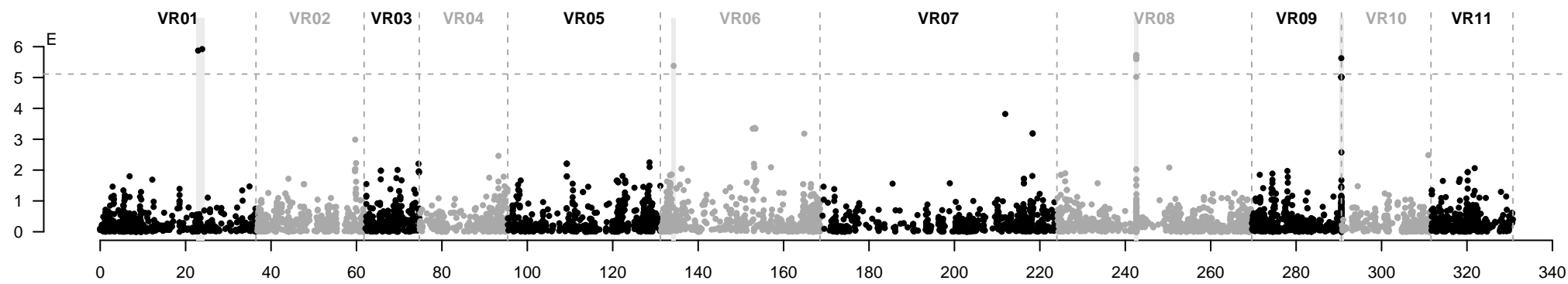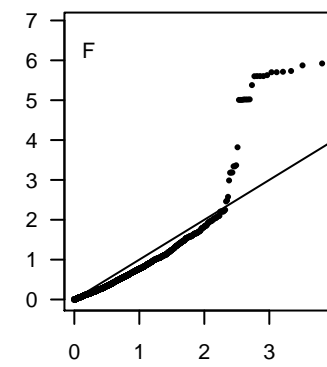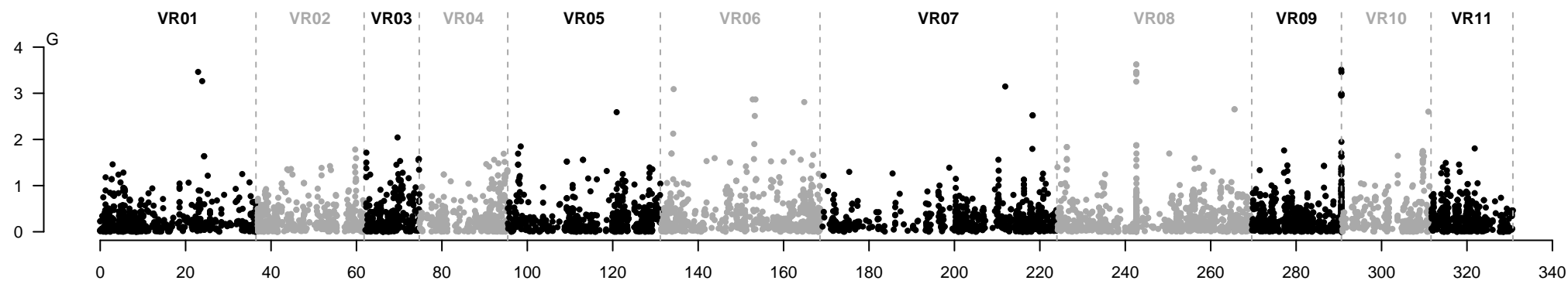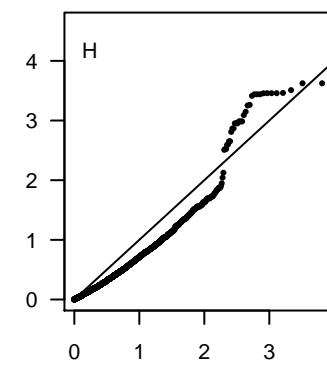

Supplement: Supplementary file 2 [file Data_Sheet_2.PDF]

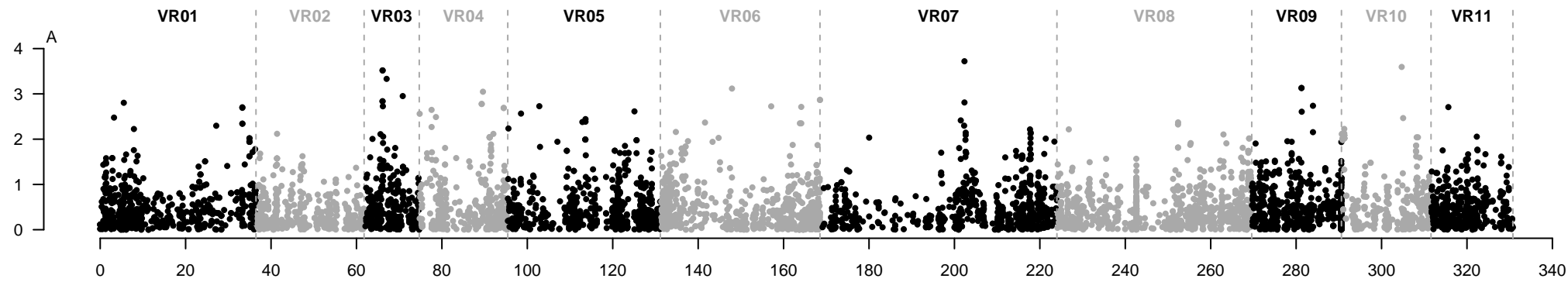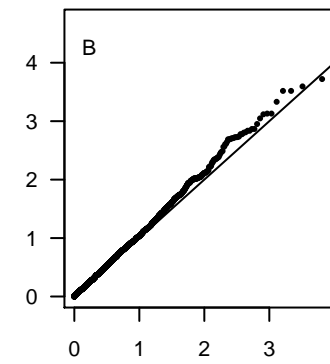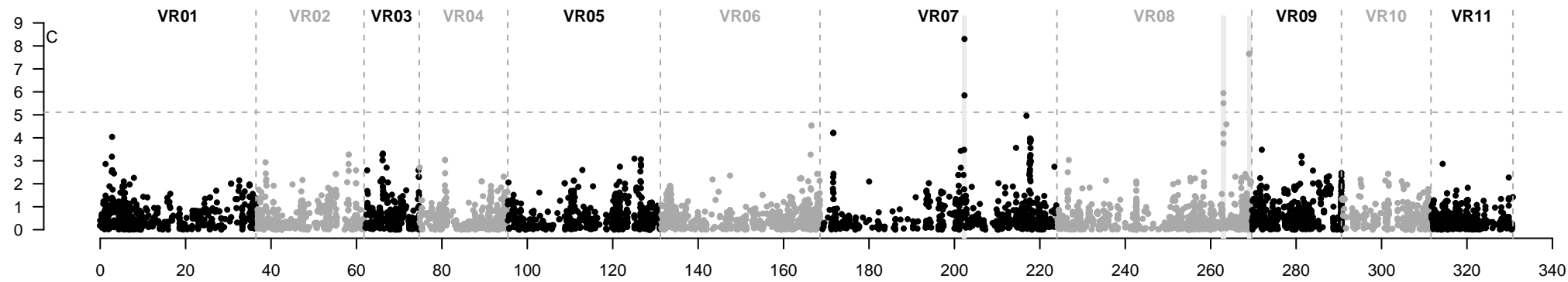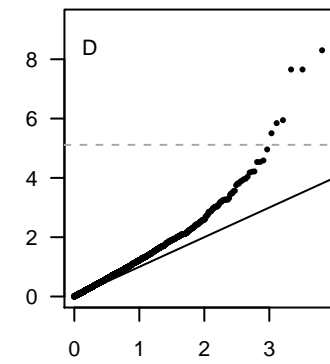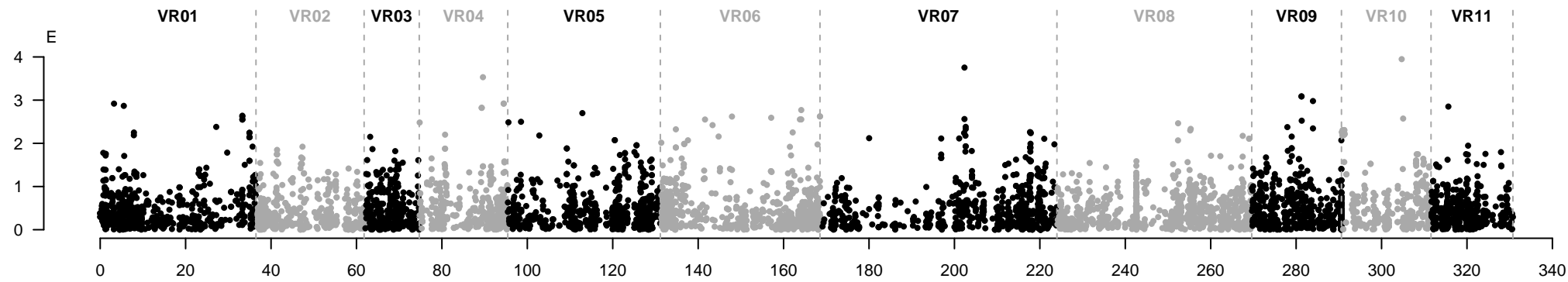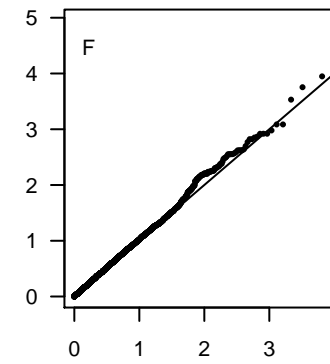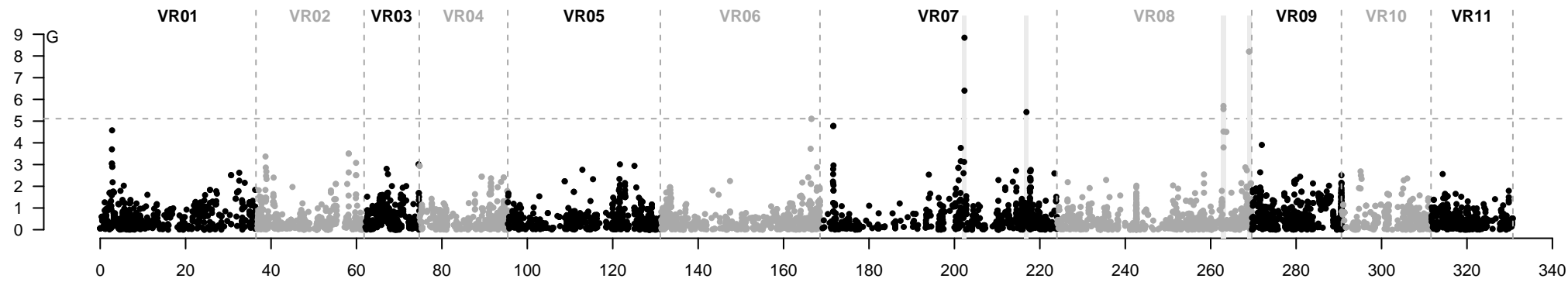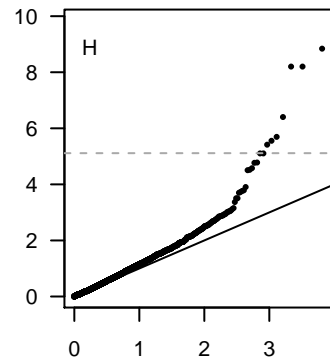

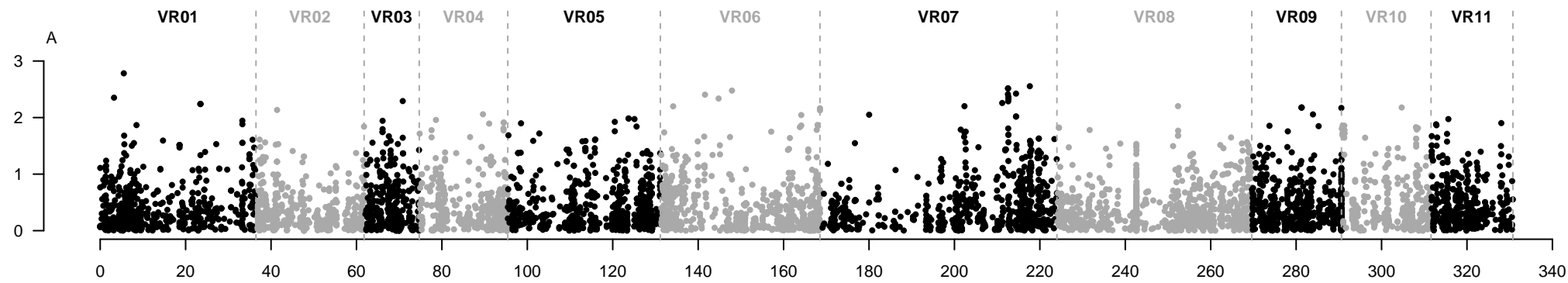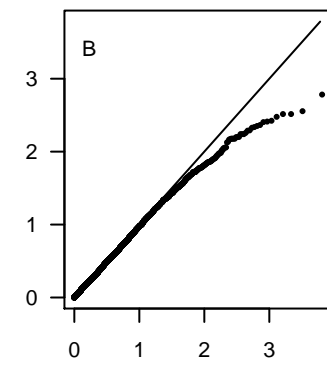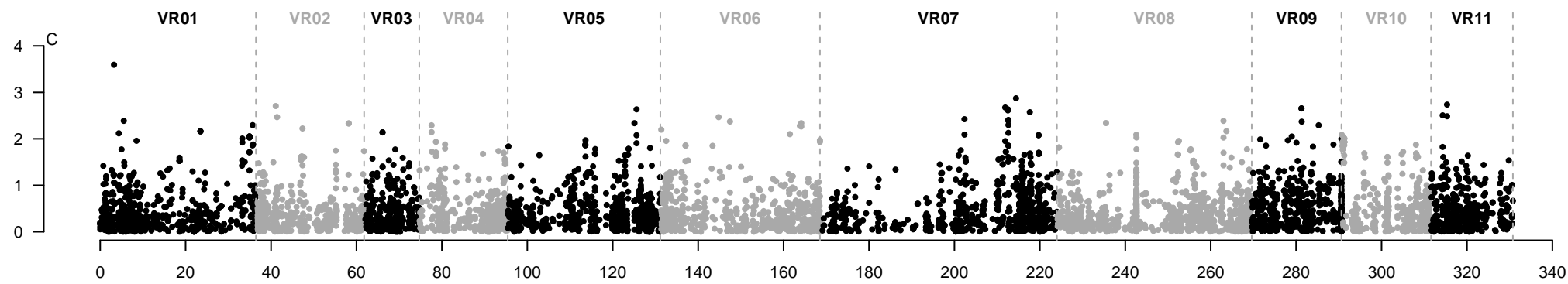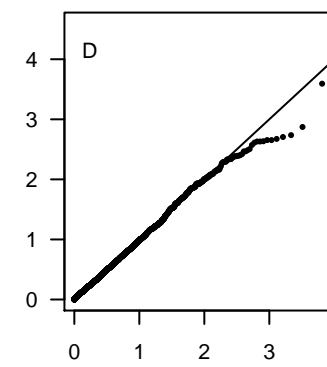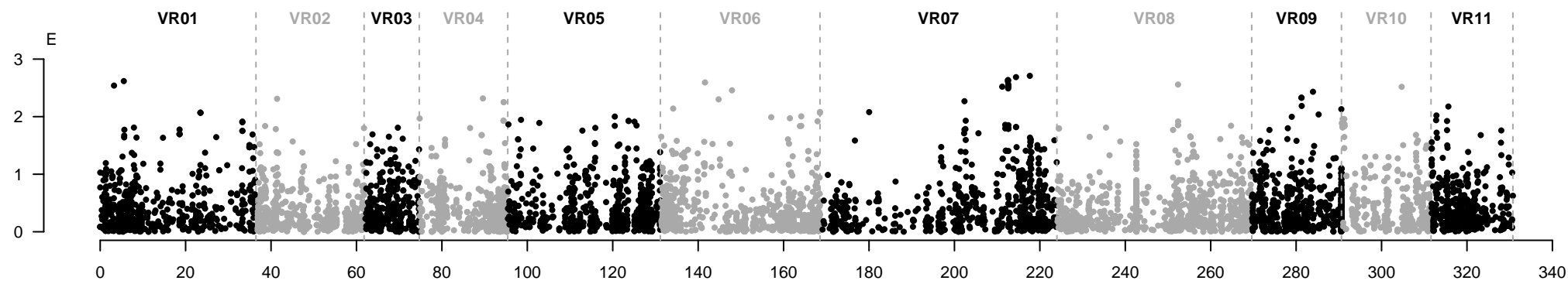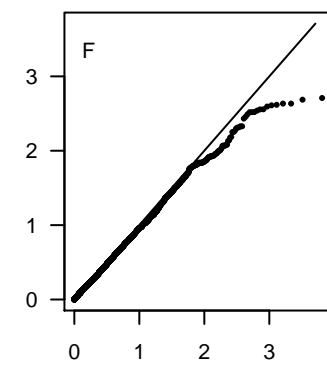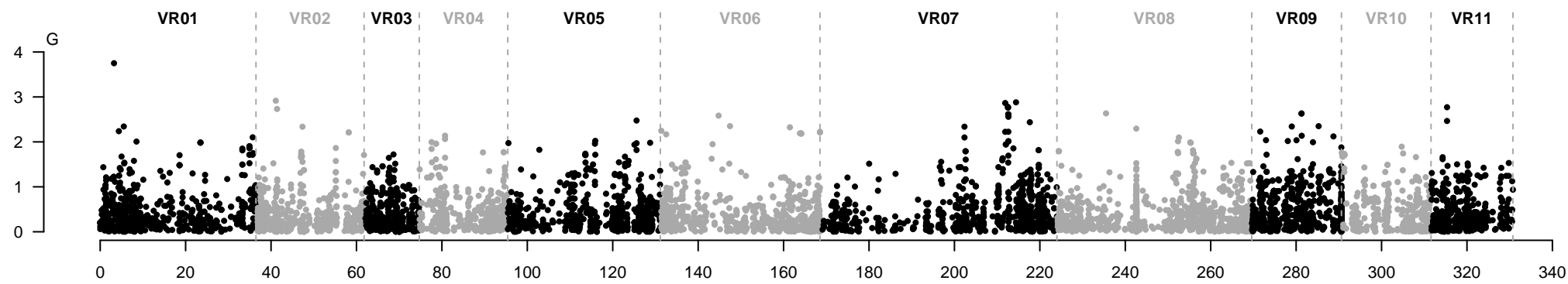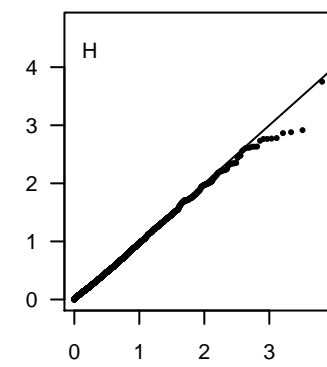

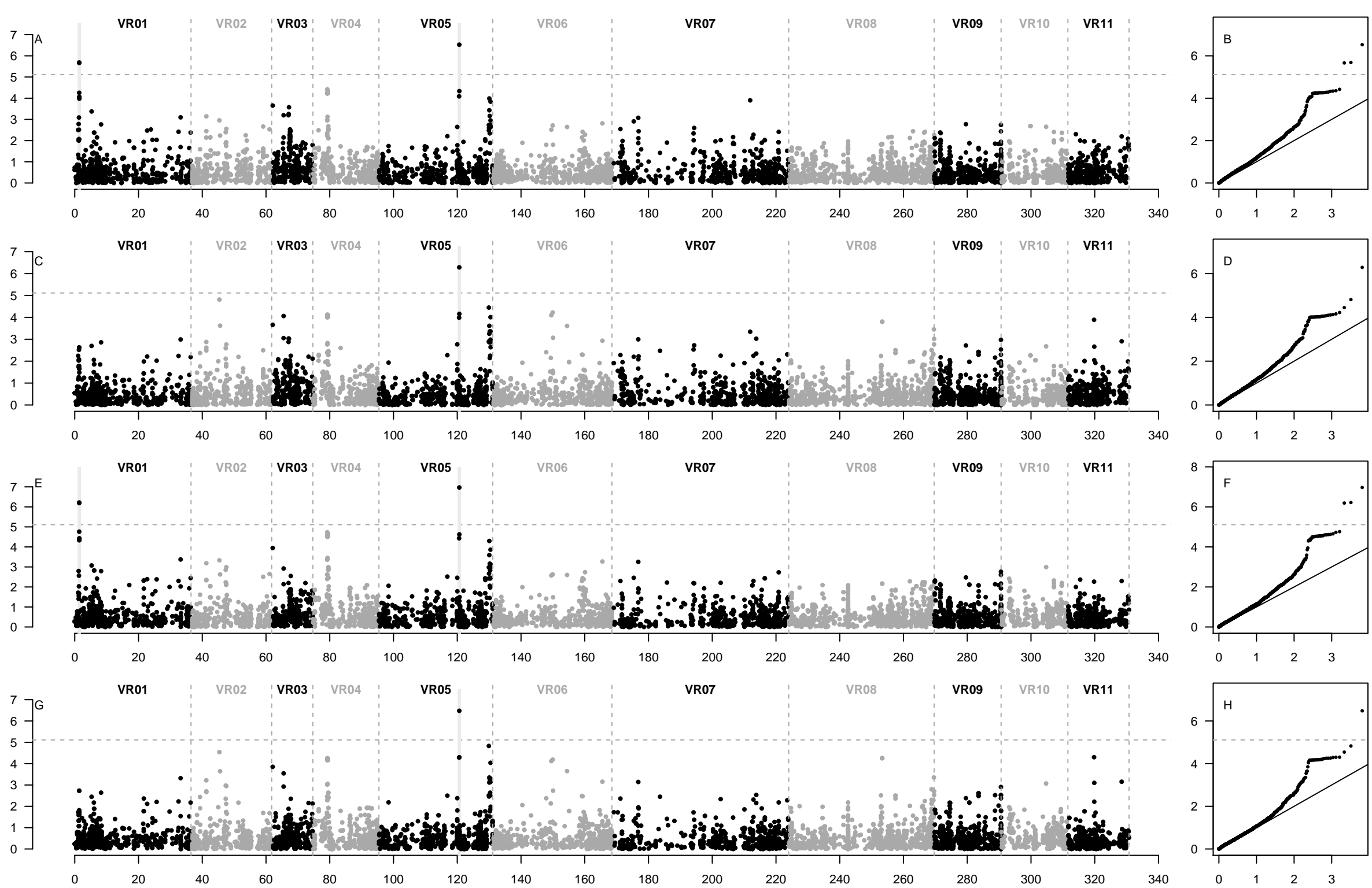

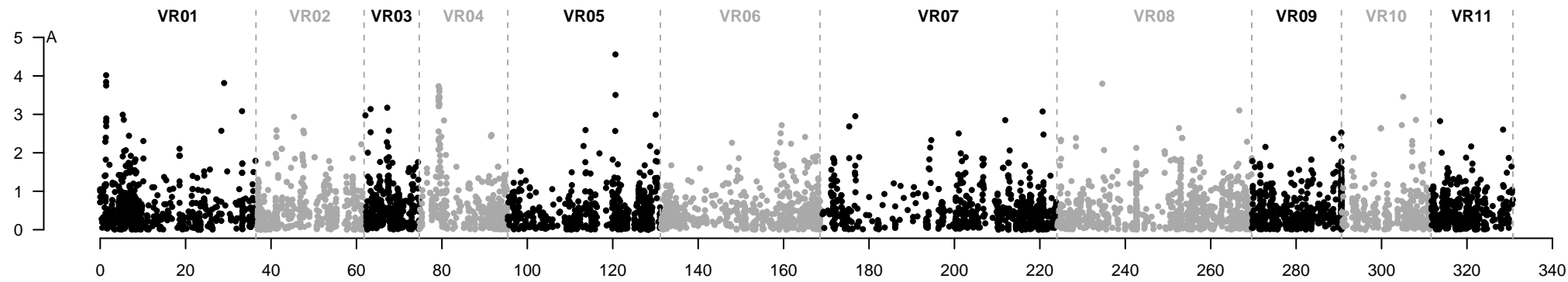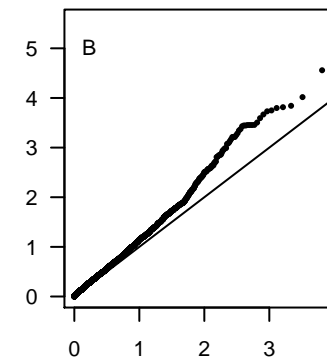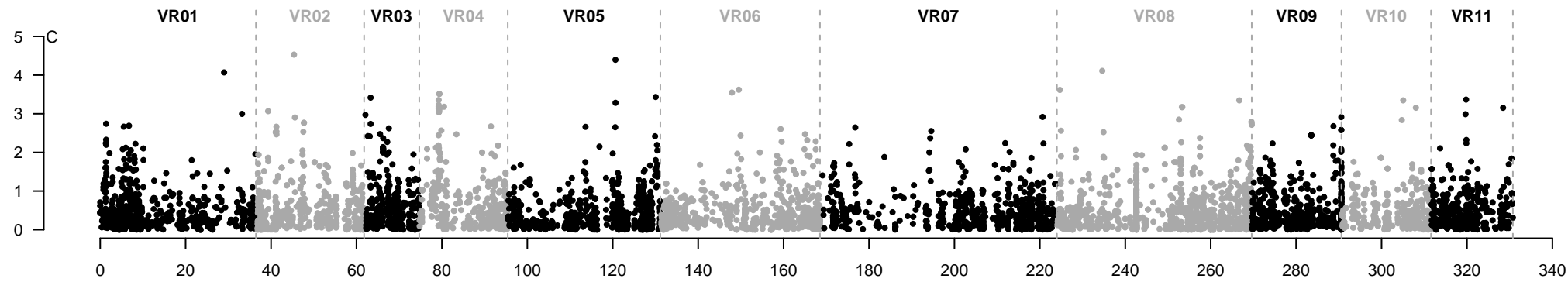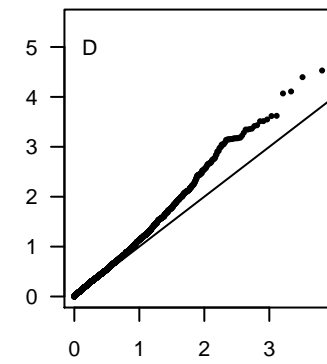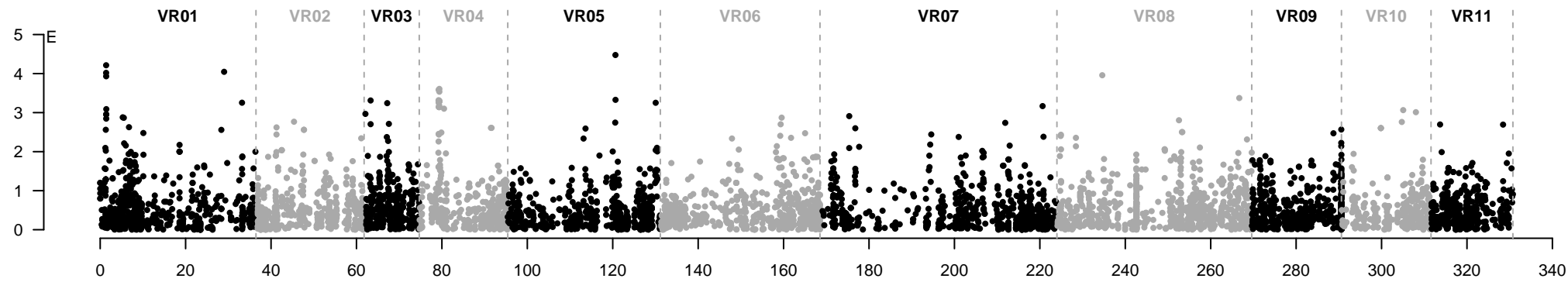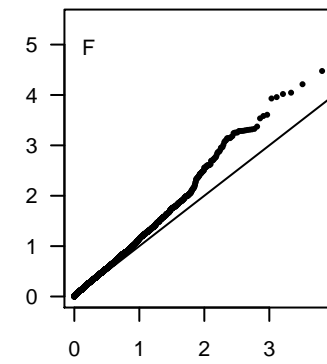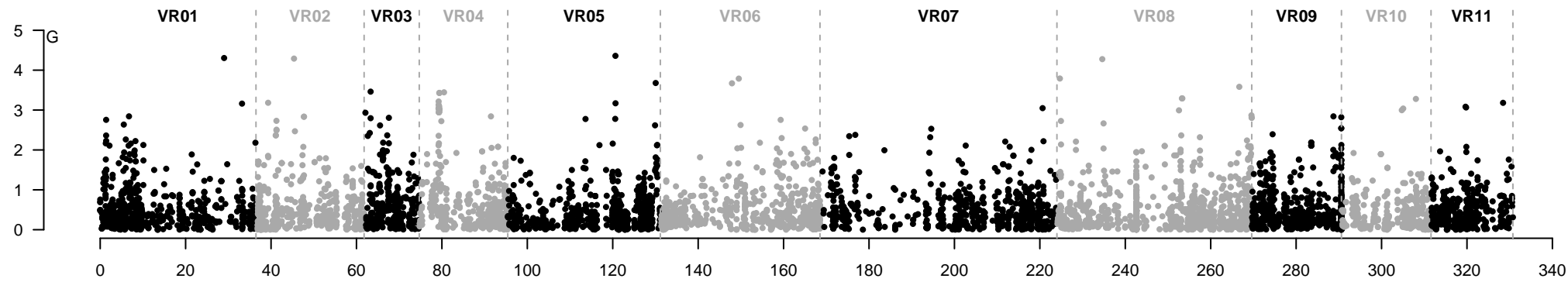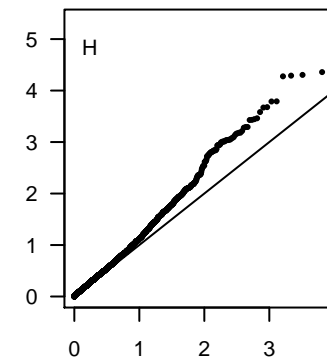

Supplement: Supplementary file 3 [file Data_Sheet_3.PDF]

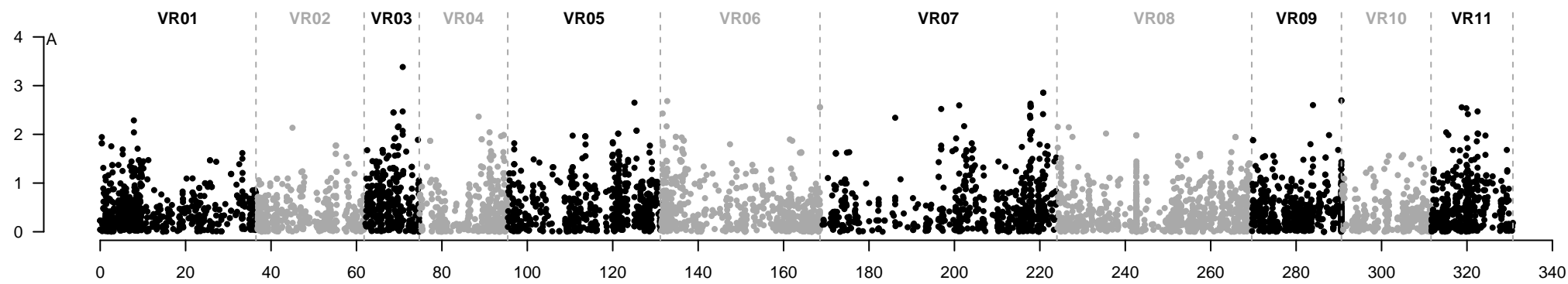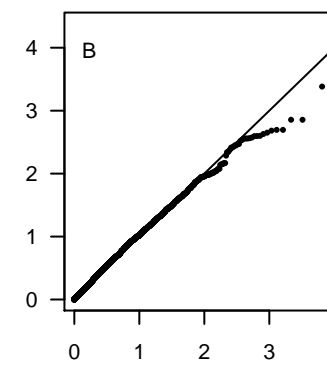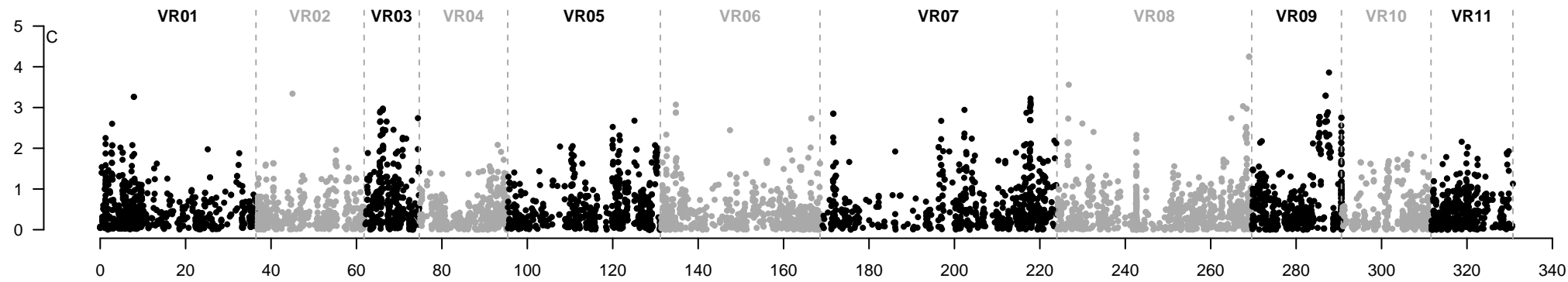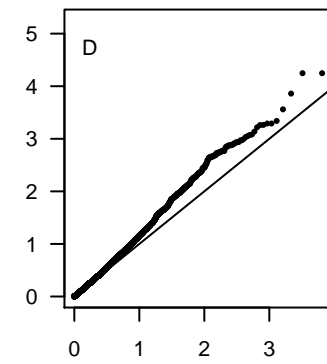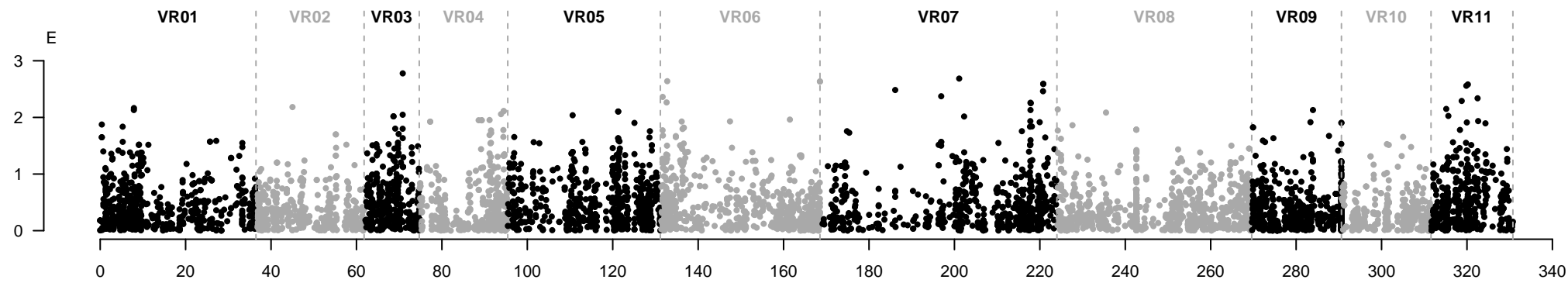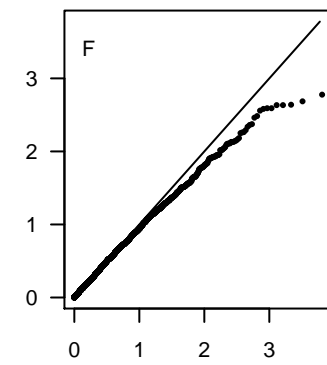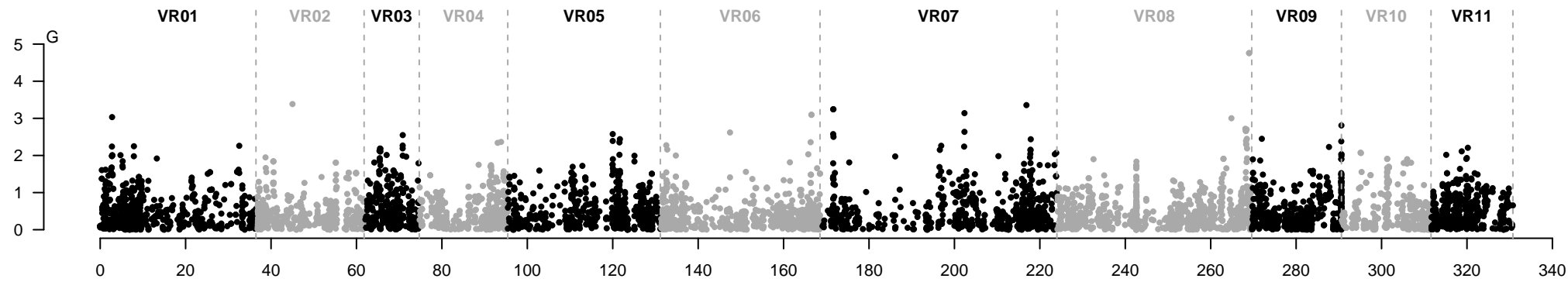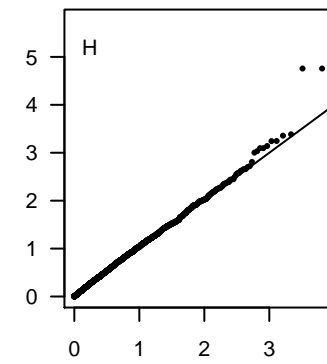

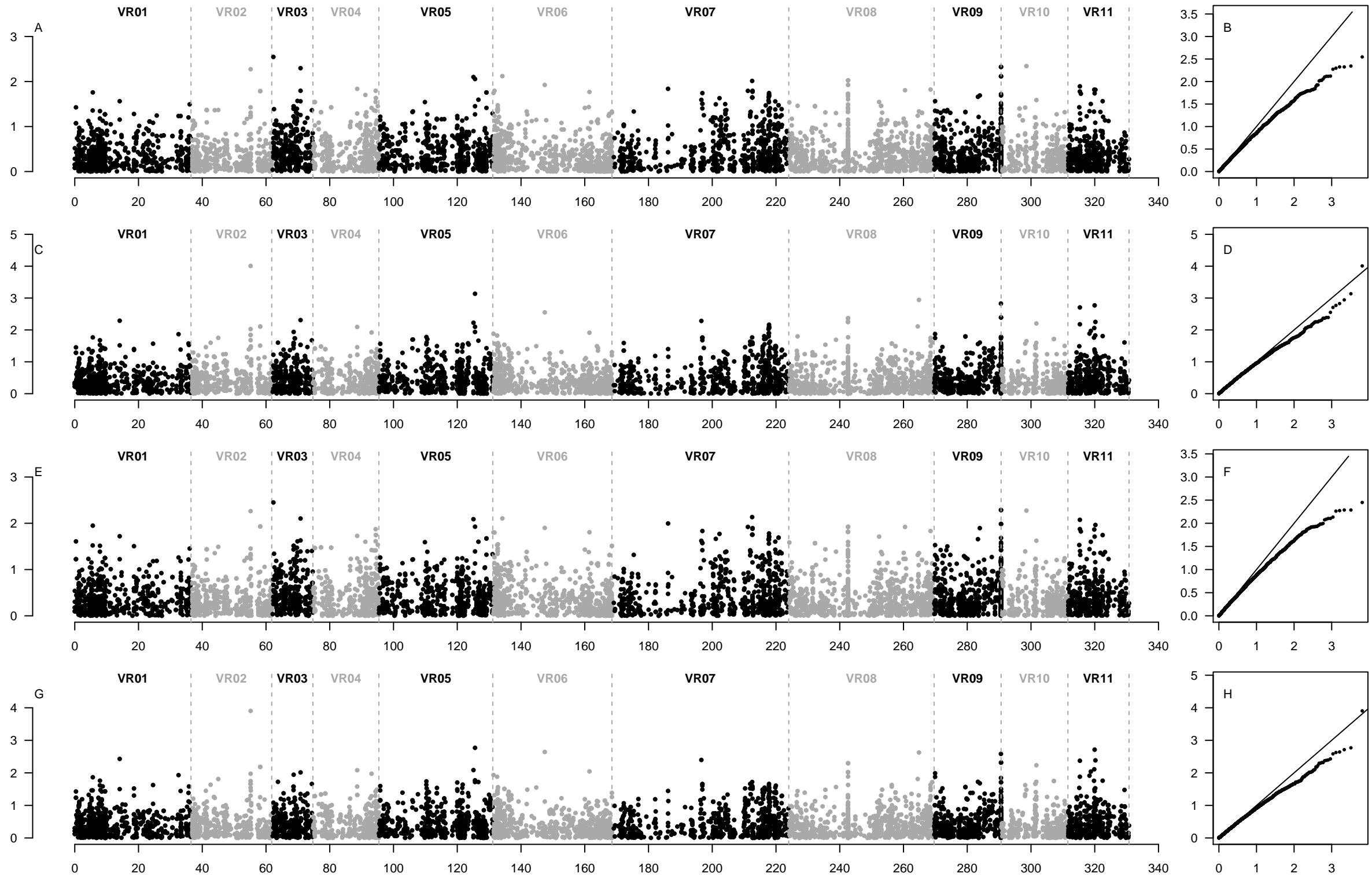

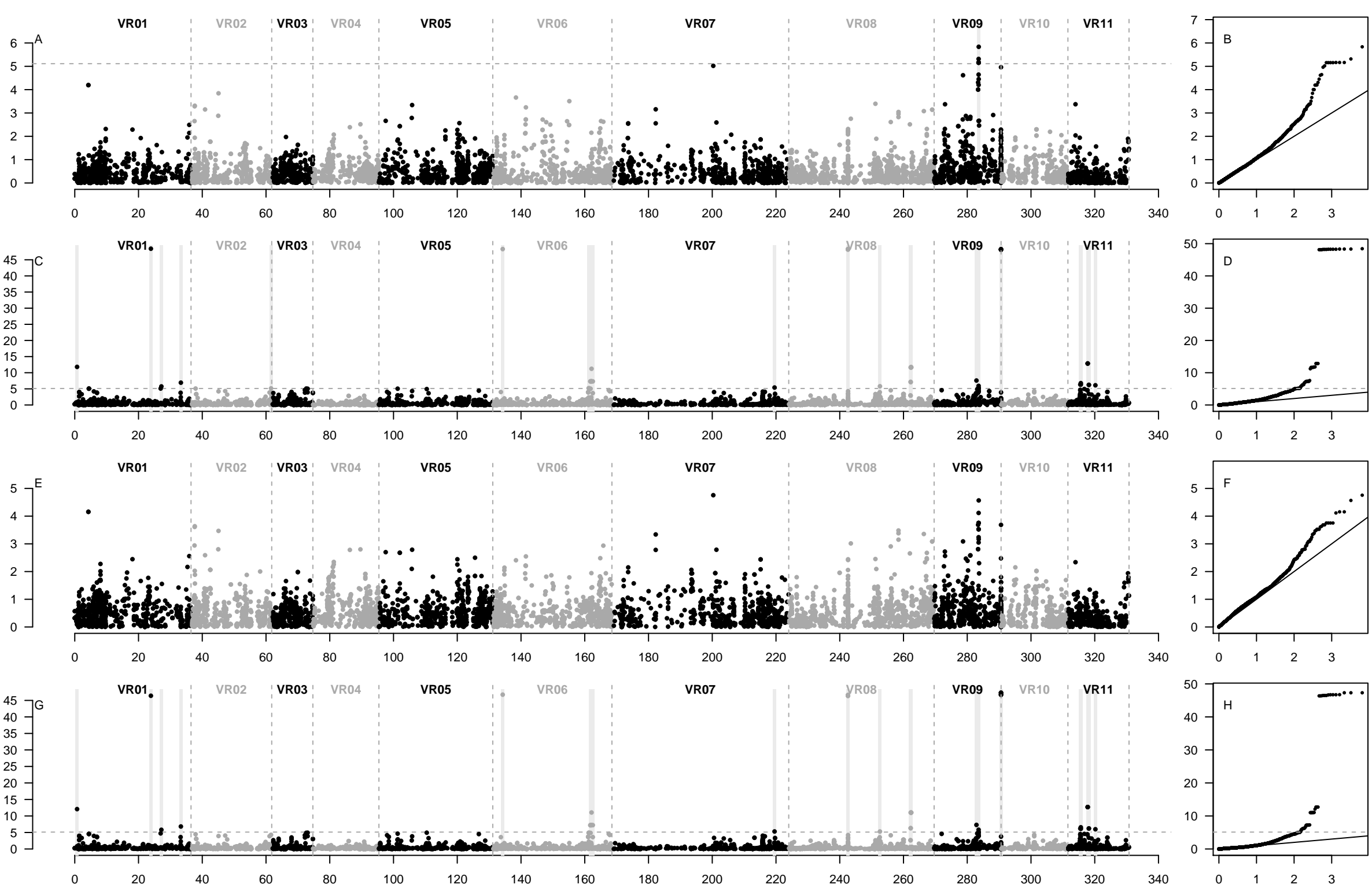

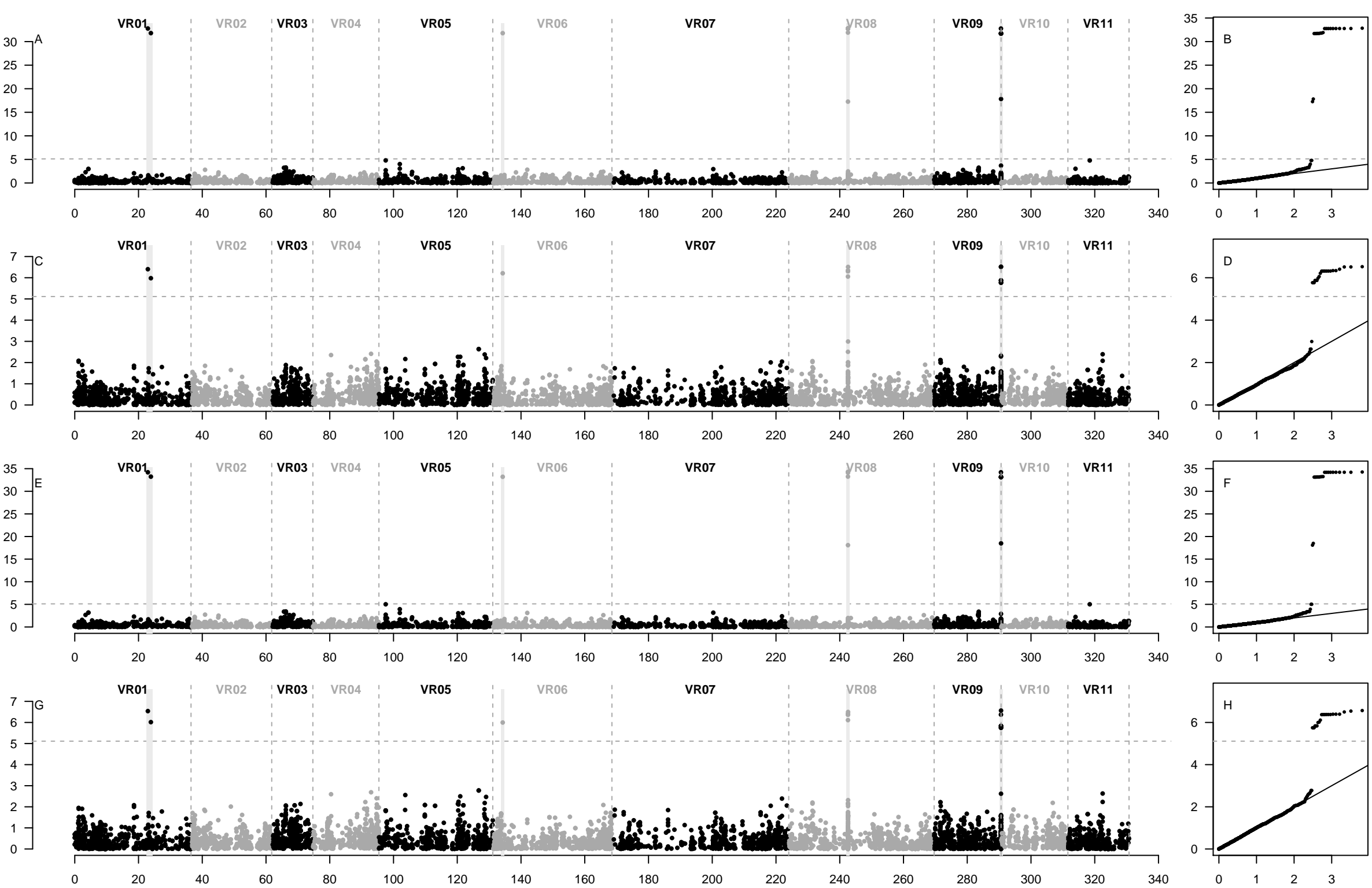

Supplement: Supplementary file 4 [file Data_Sheet_4.PDF]

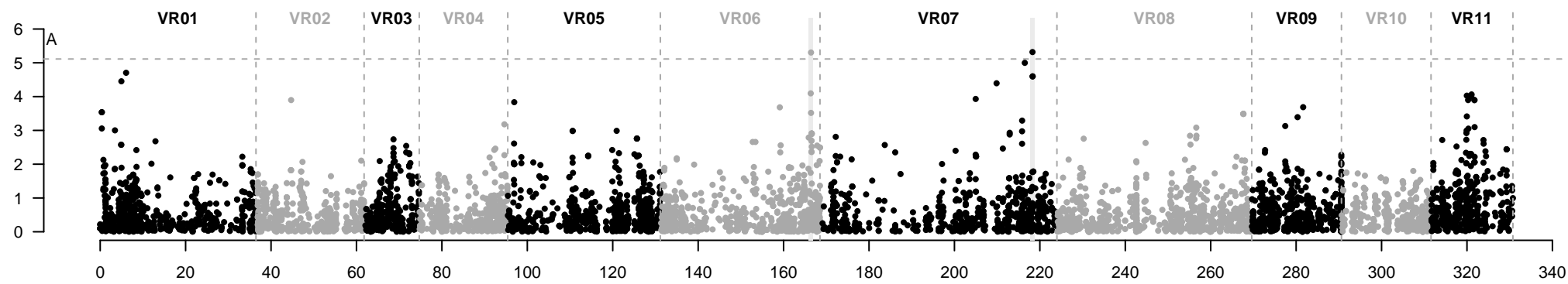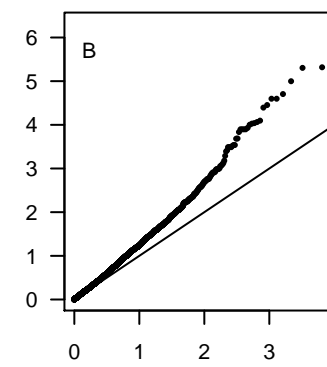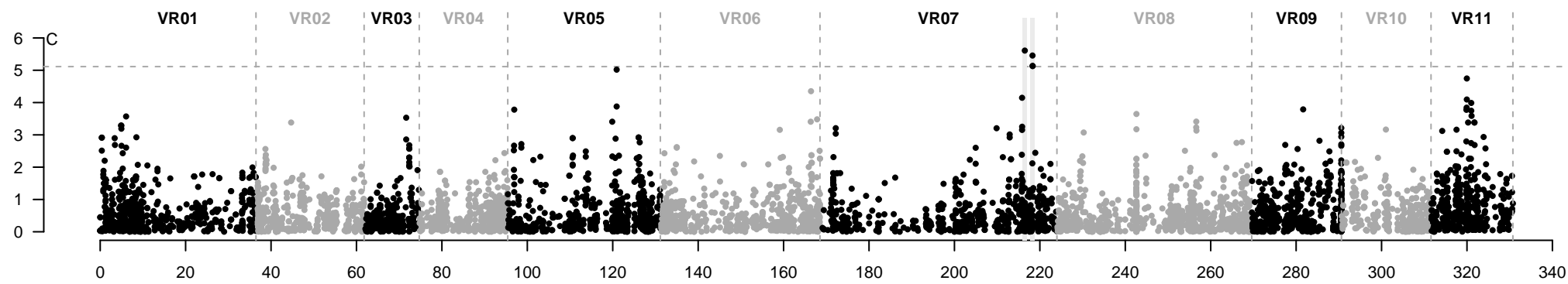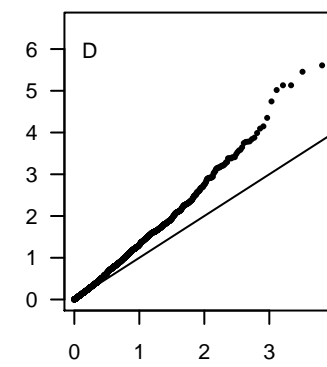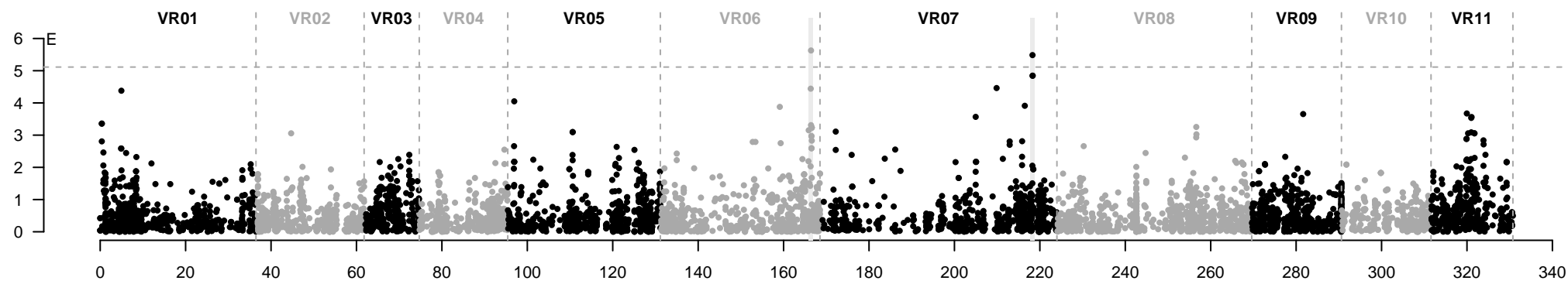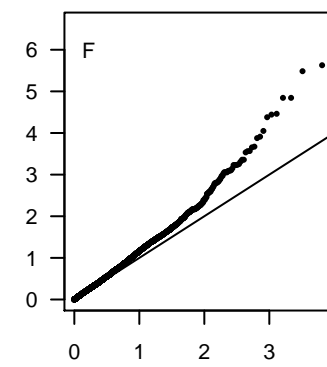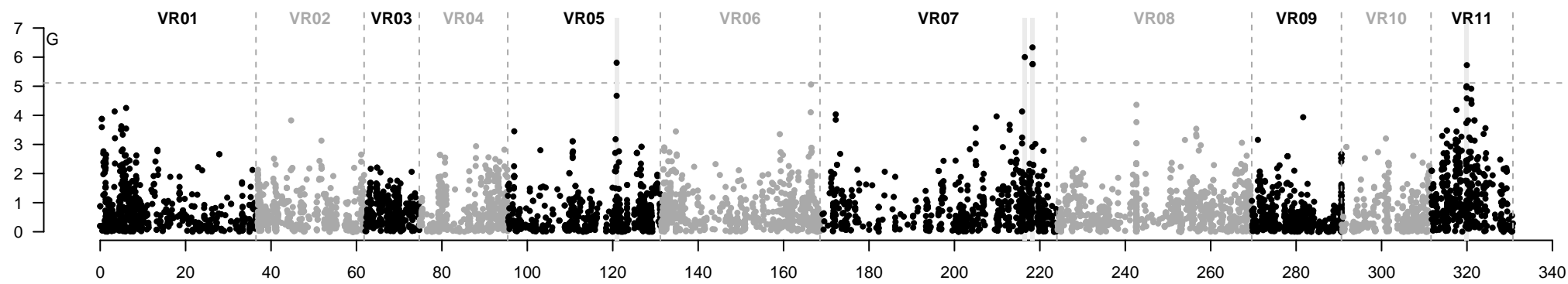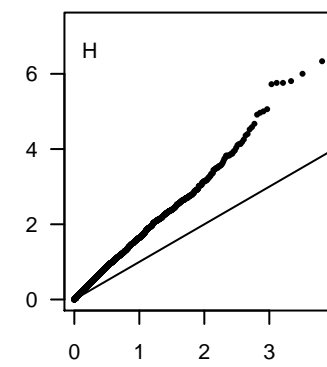

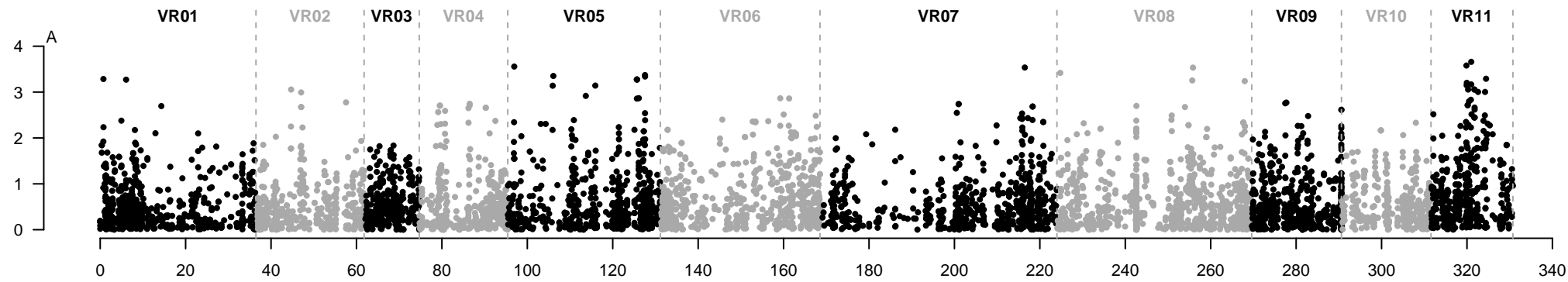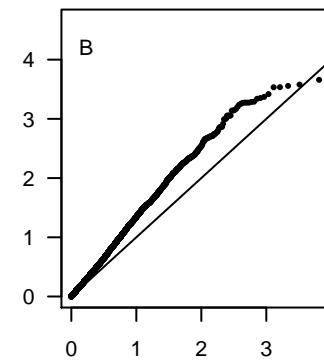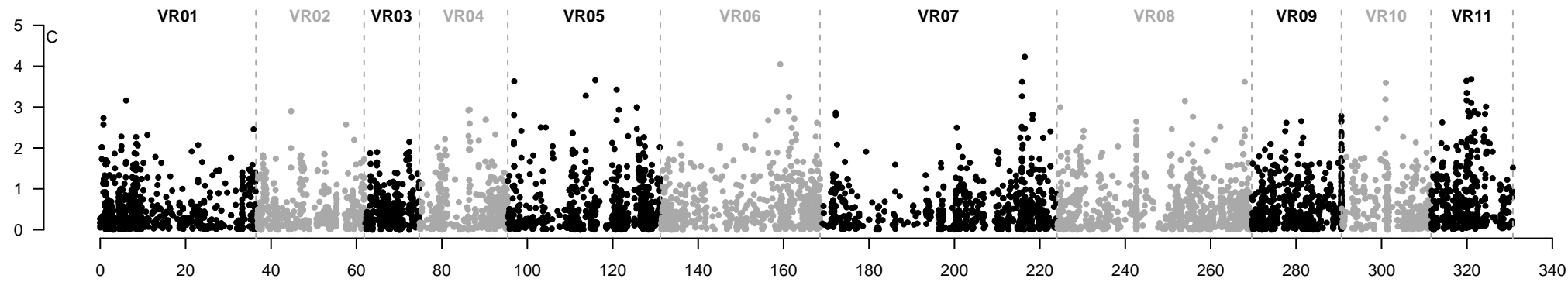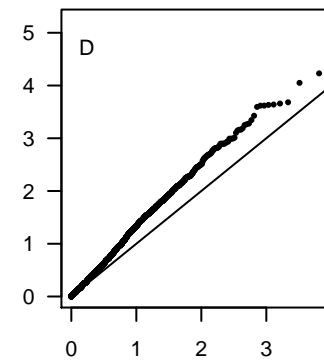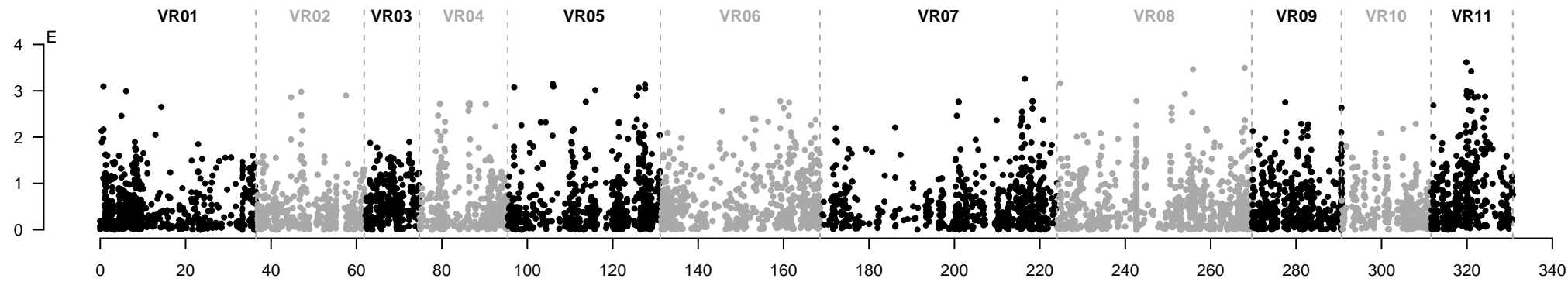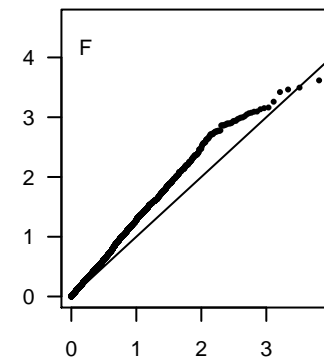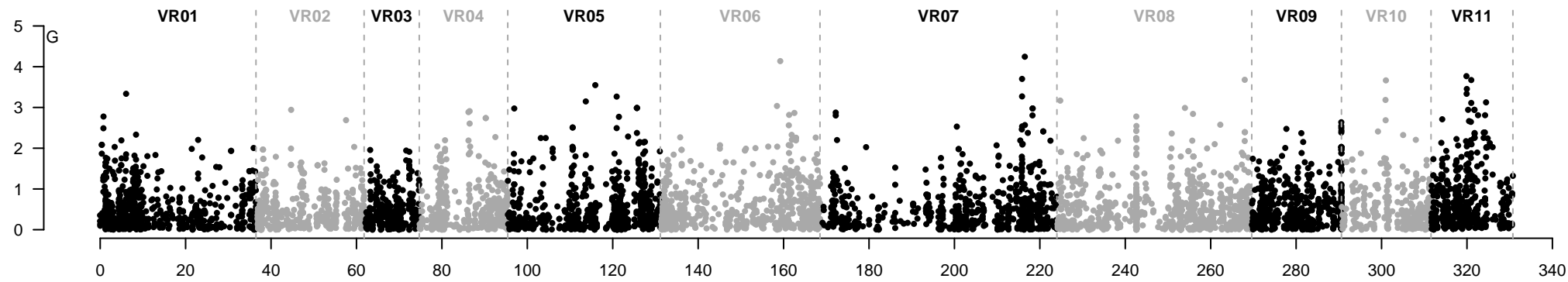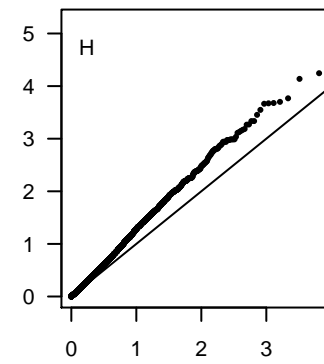

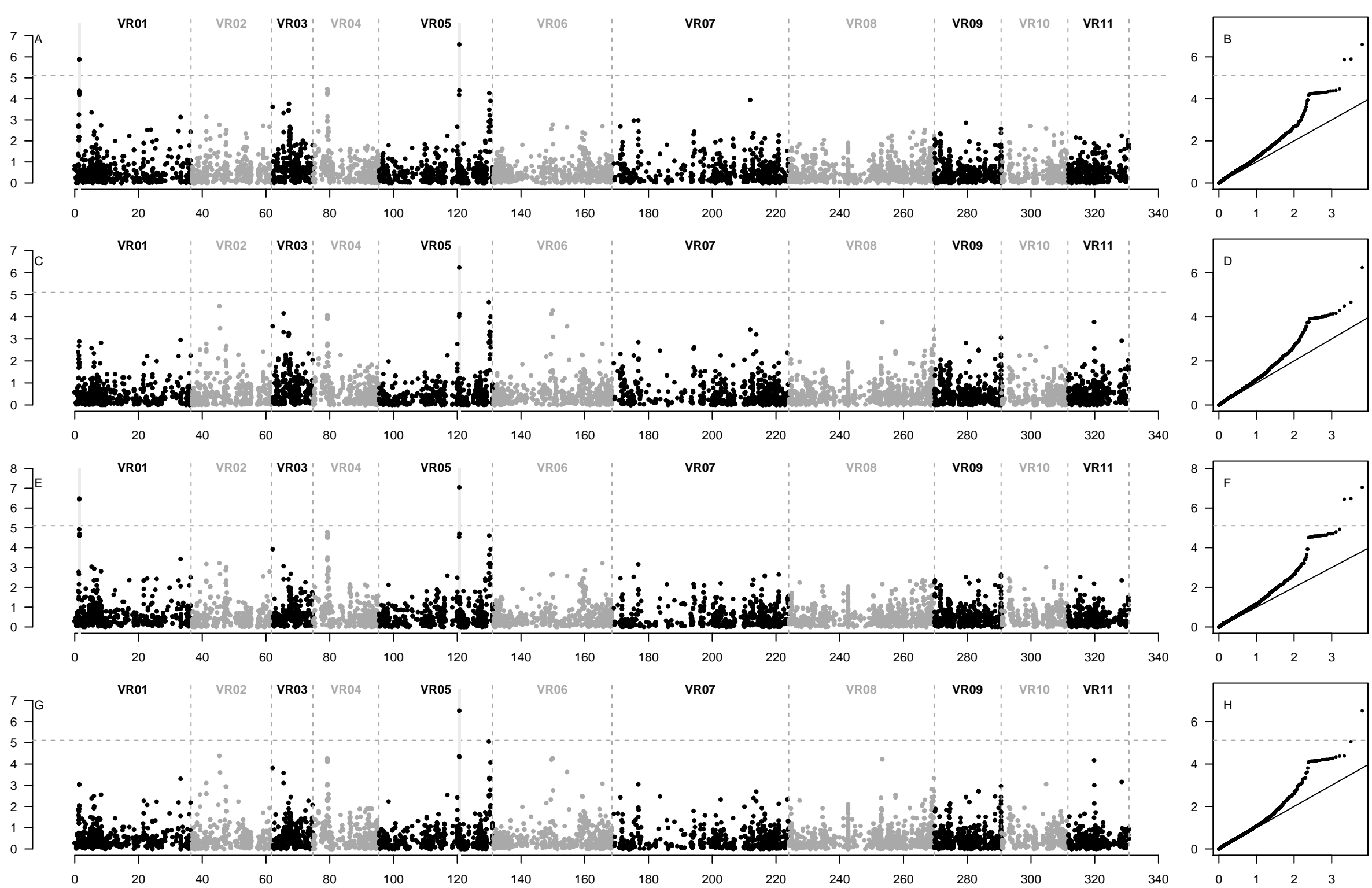

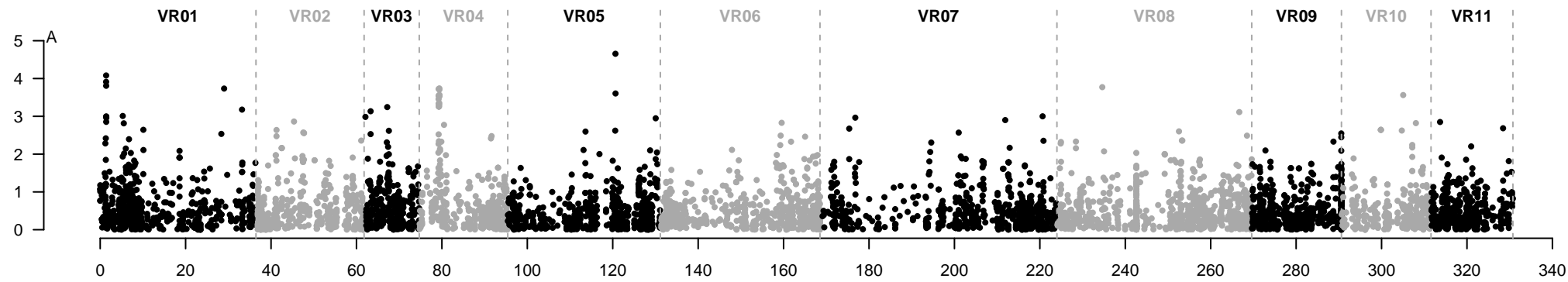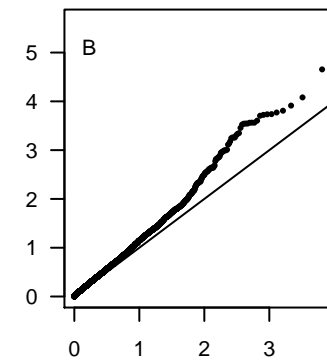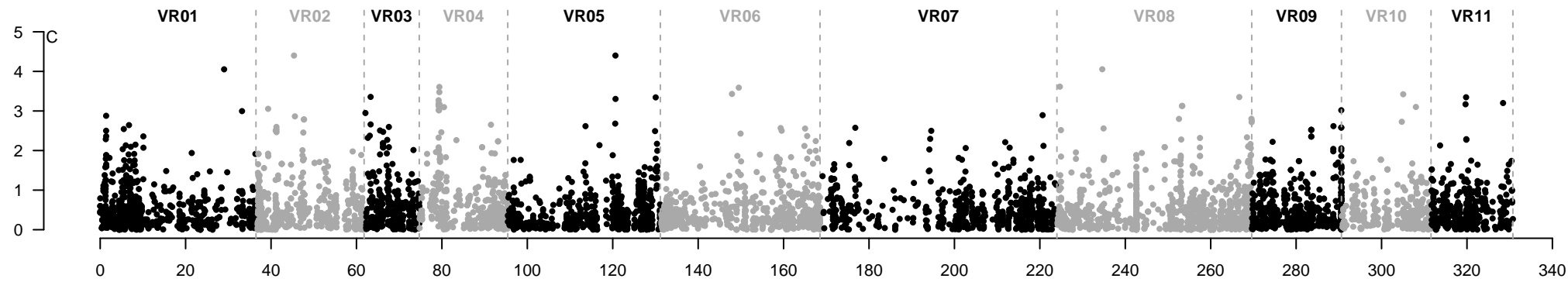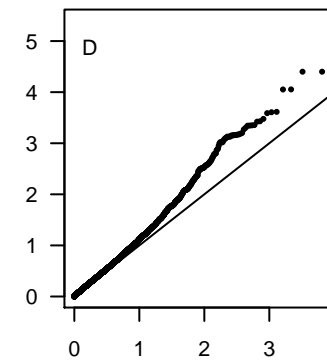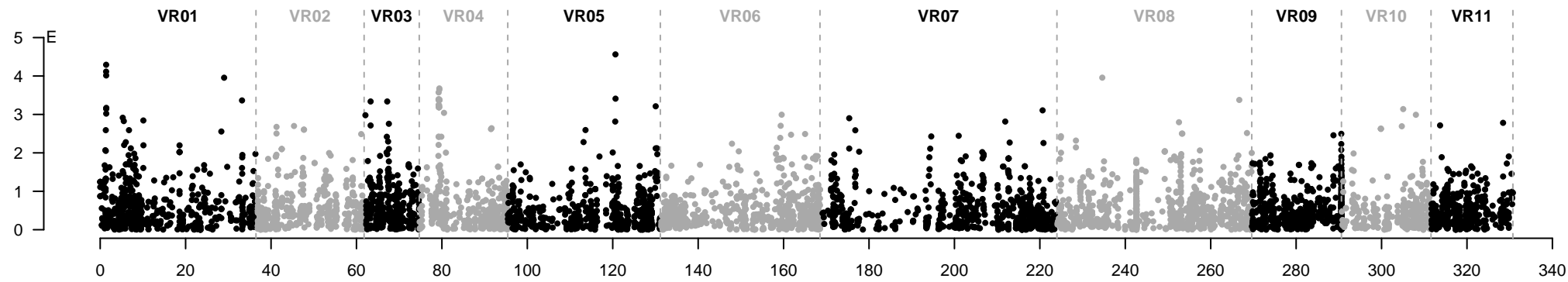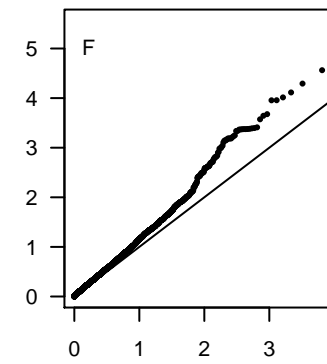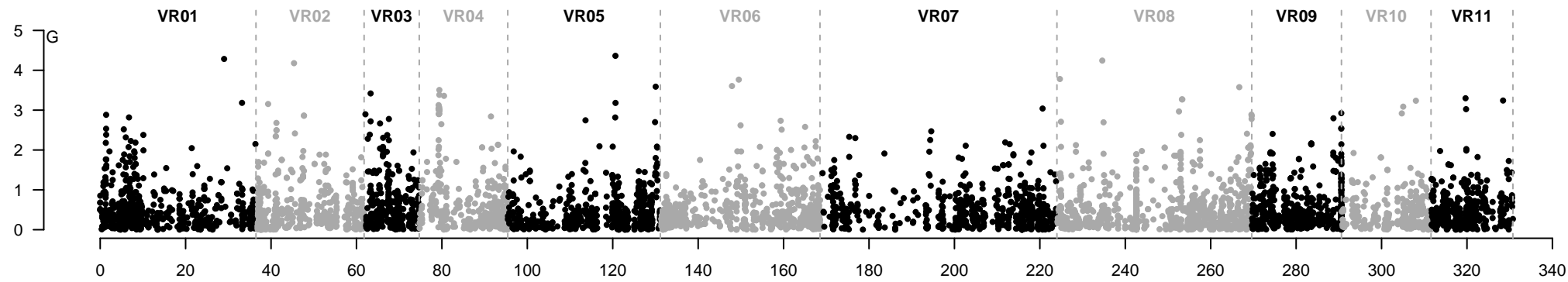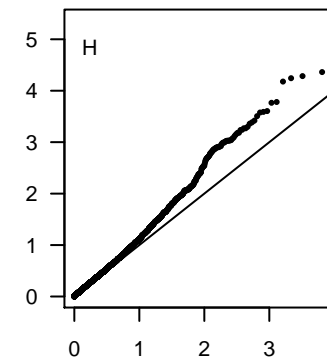

Supplement: Supplementary file 5 [file Data_Sheet_5.PDF]

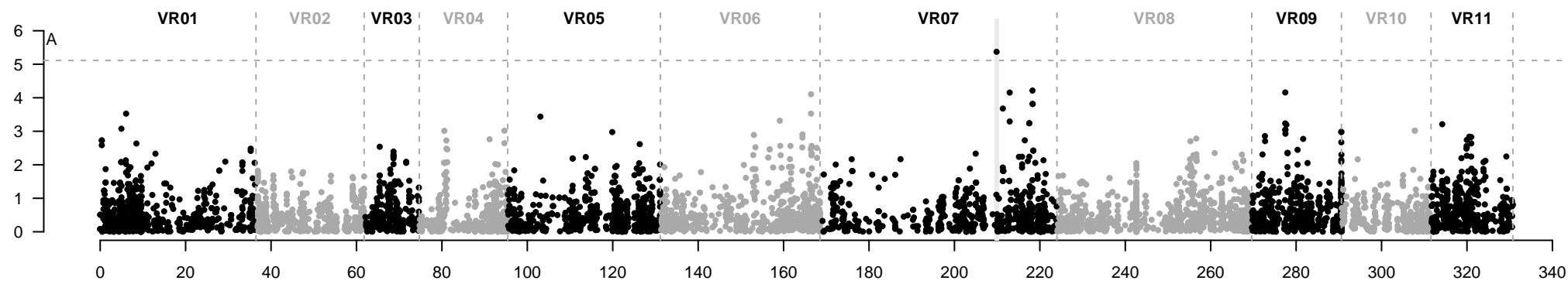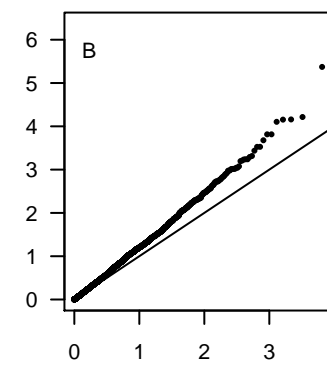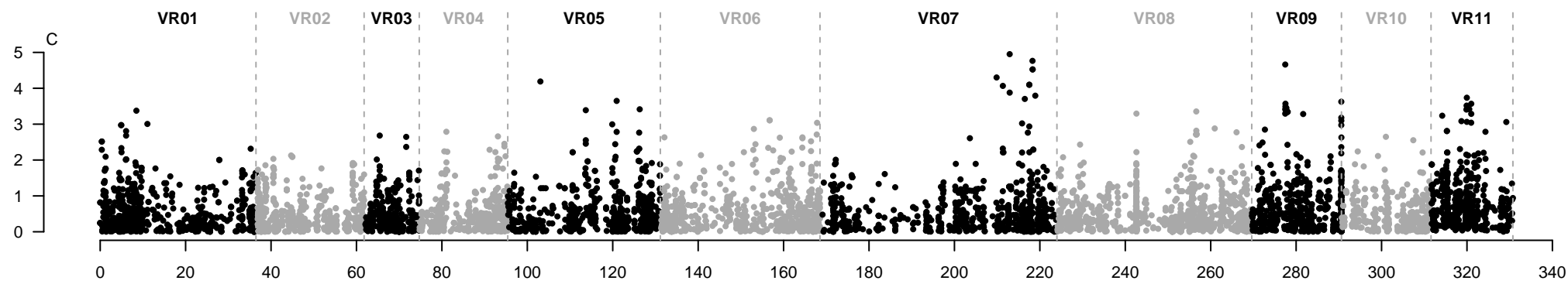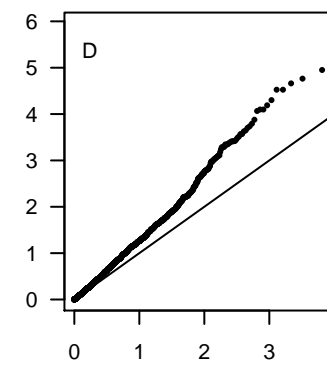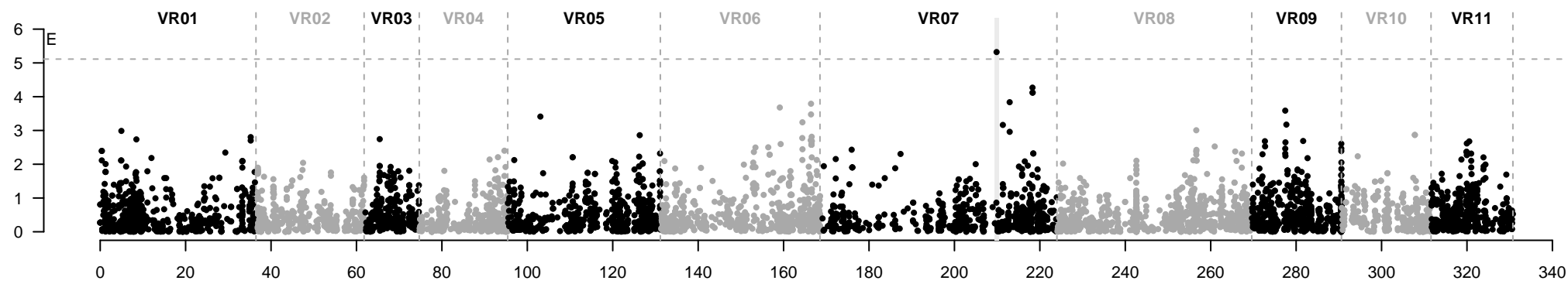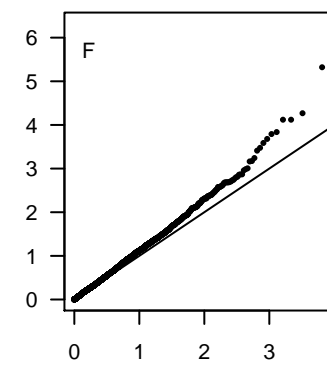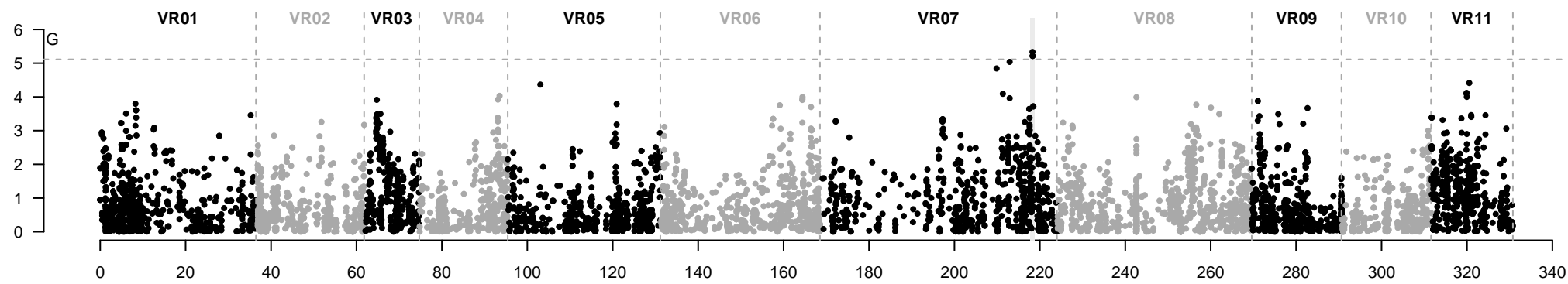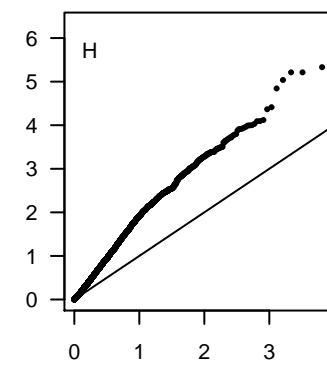

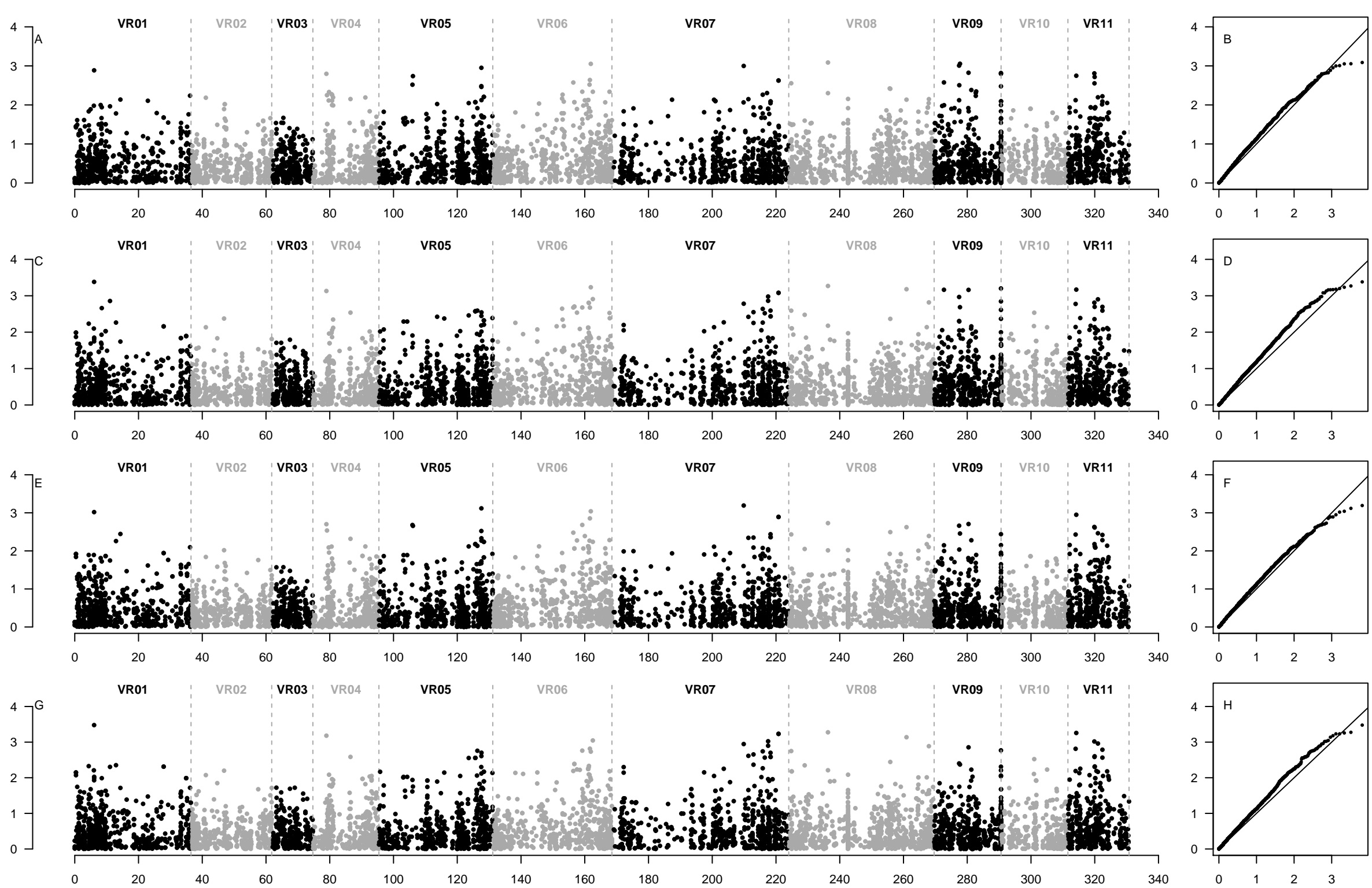

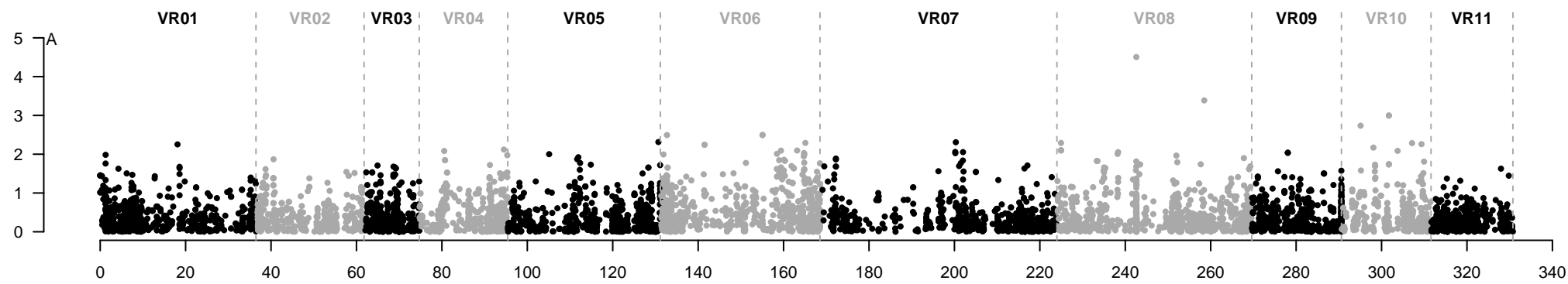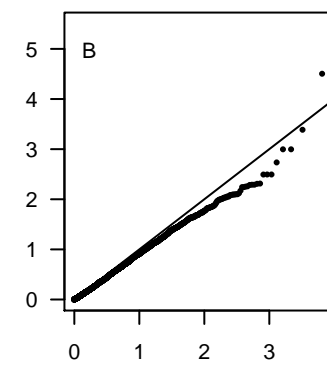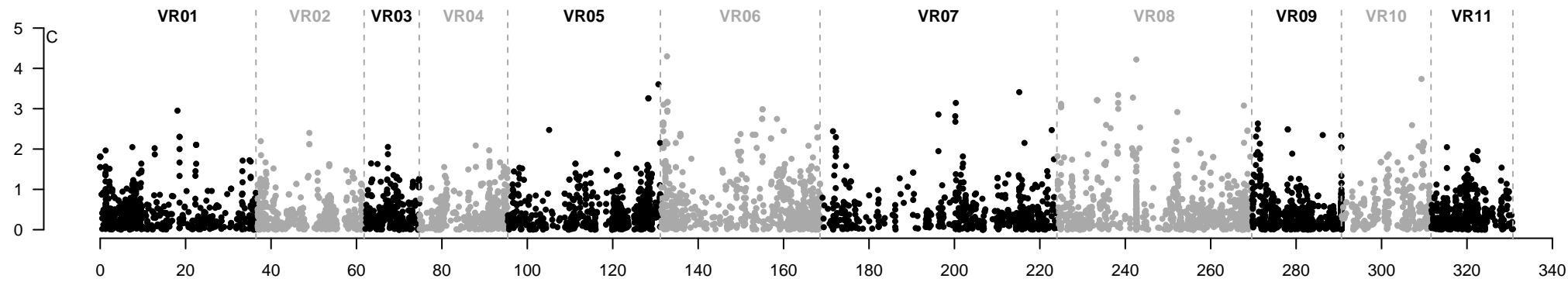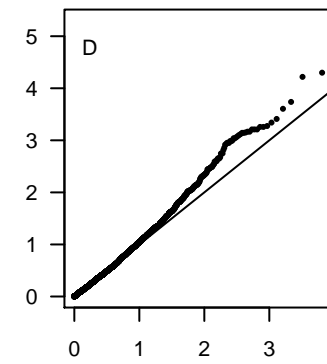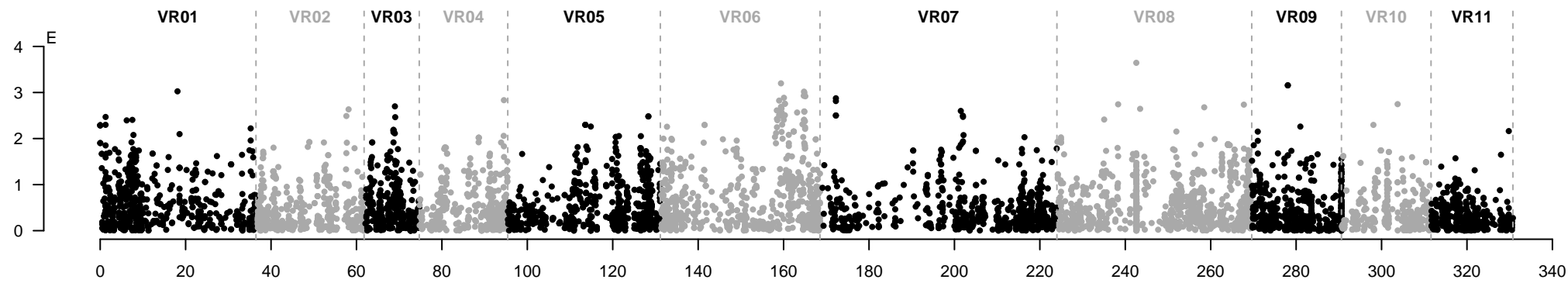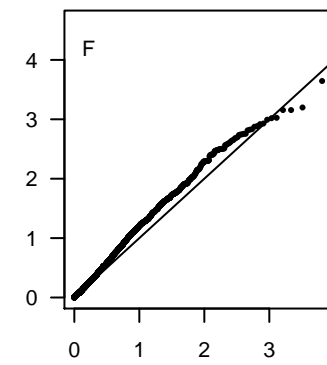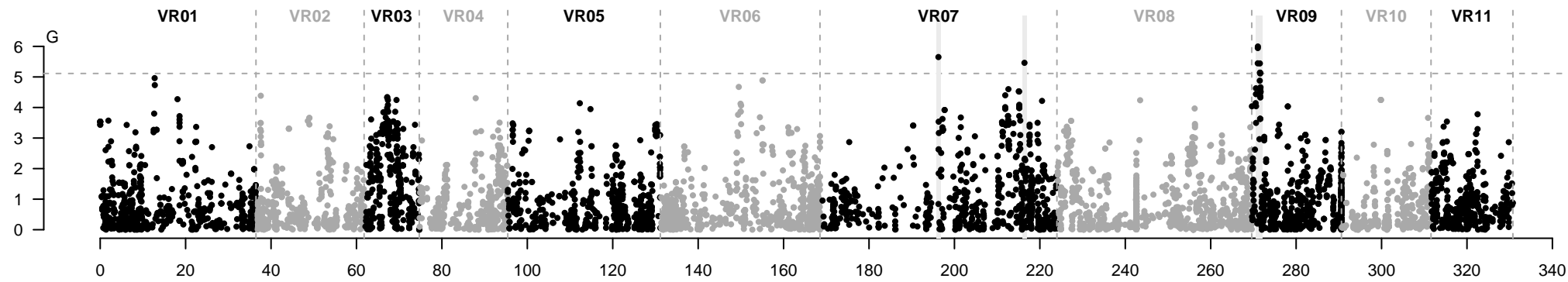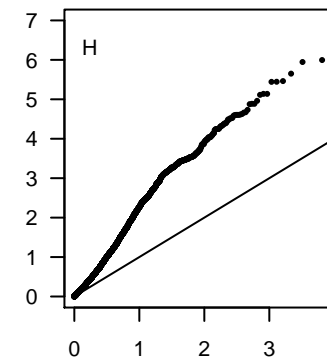

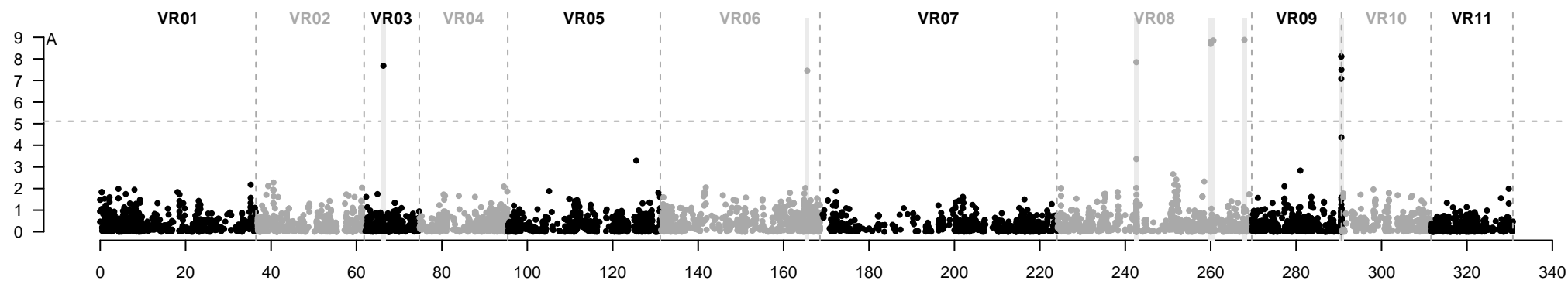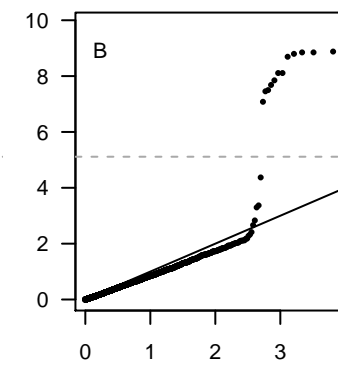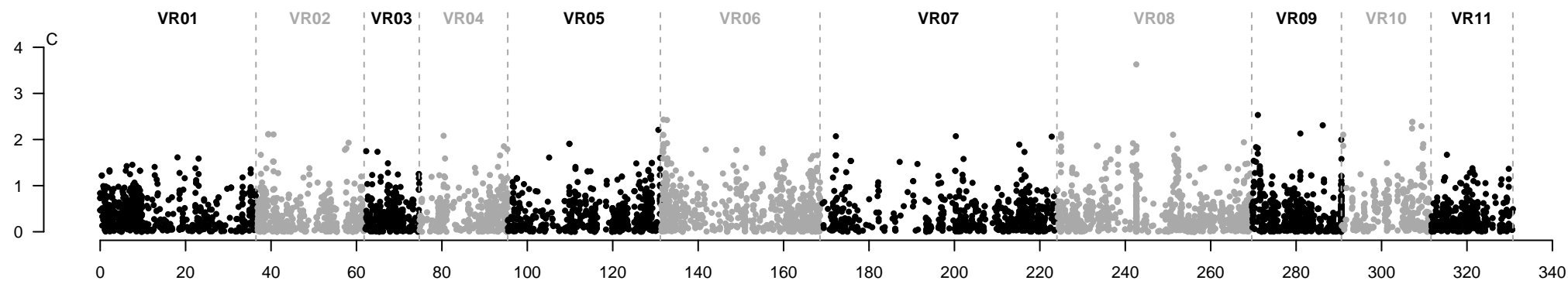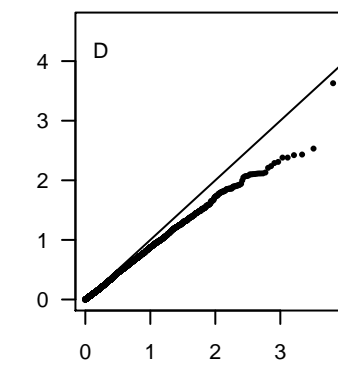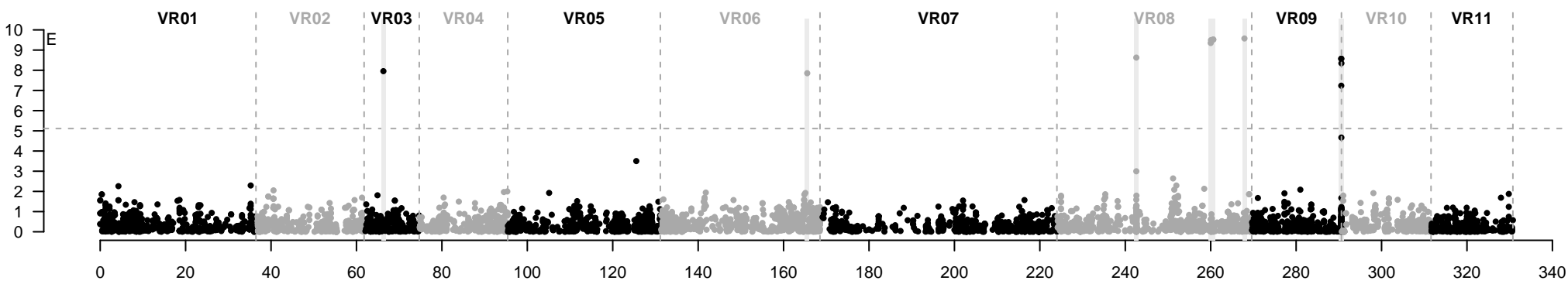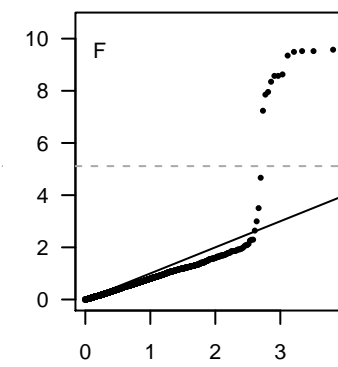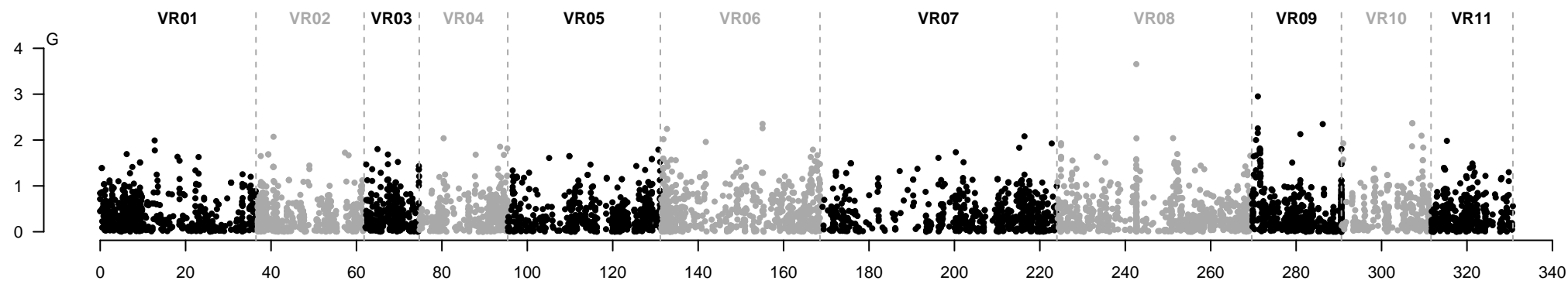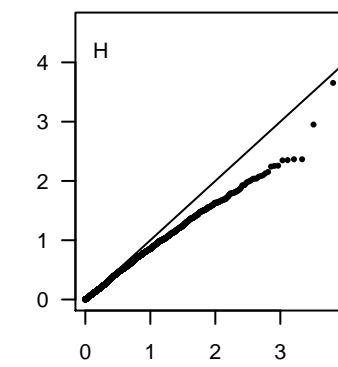

Supplement: Supplementary file 6 [file Data_Sheet_6.PDF]

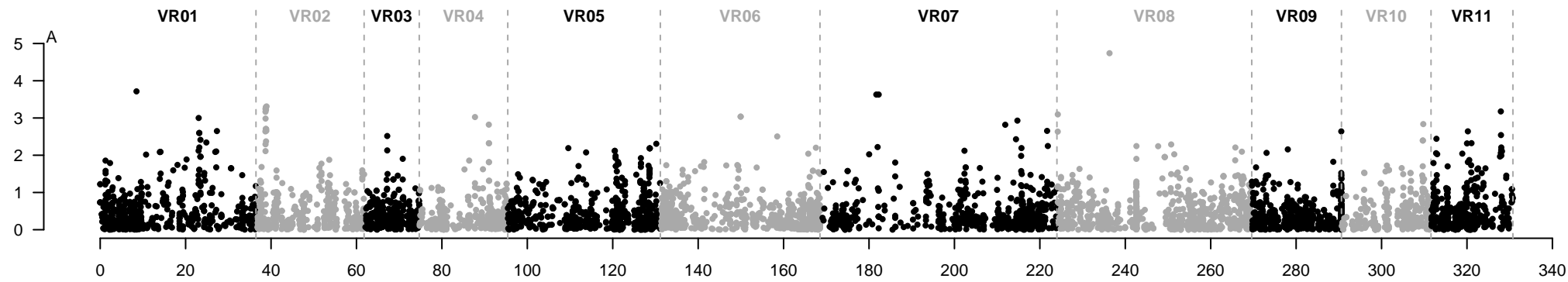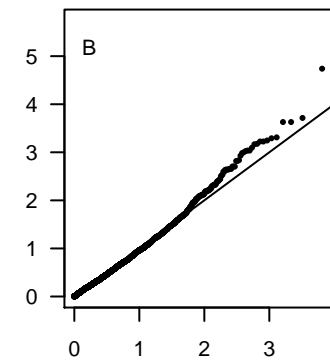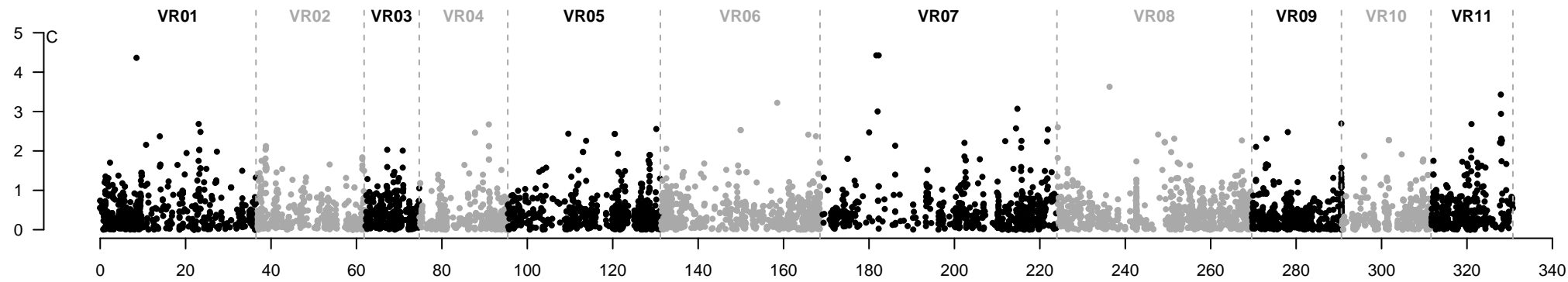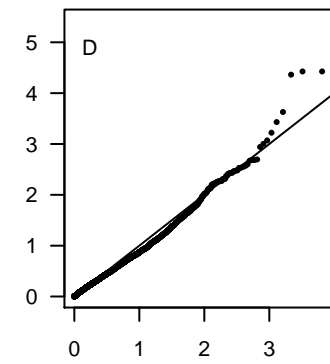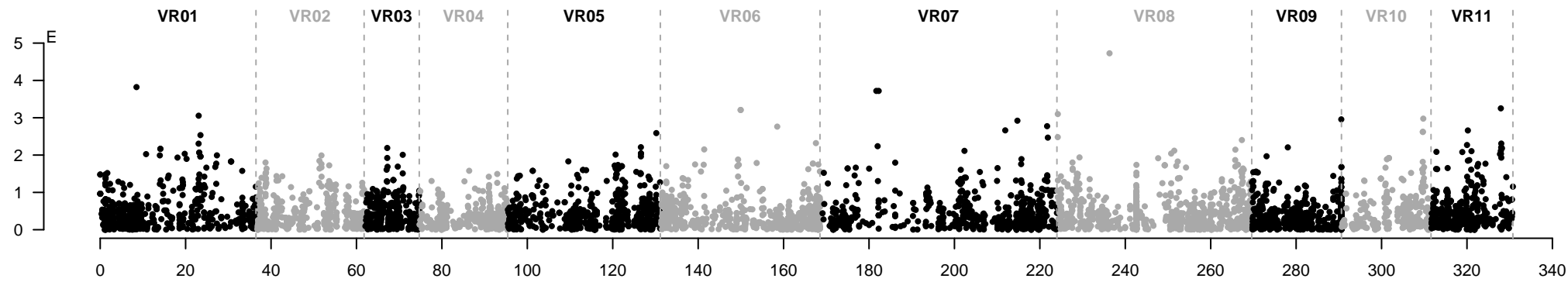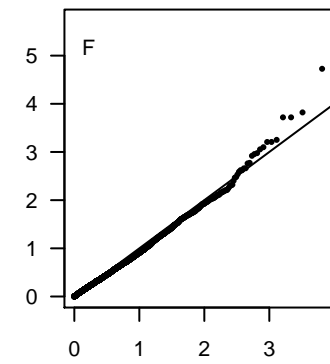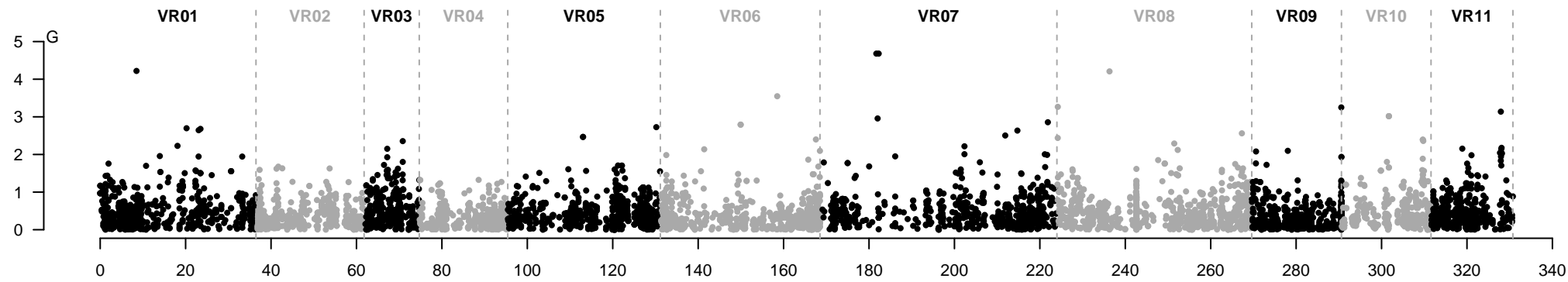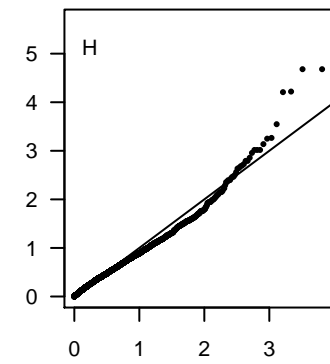

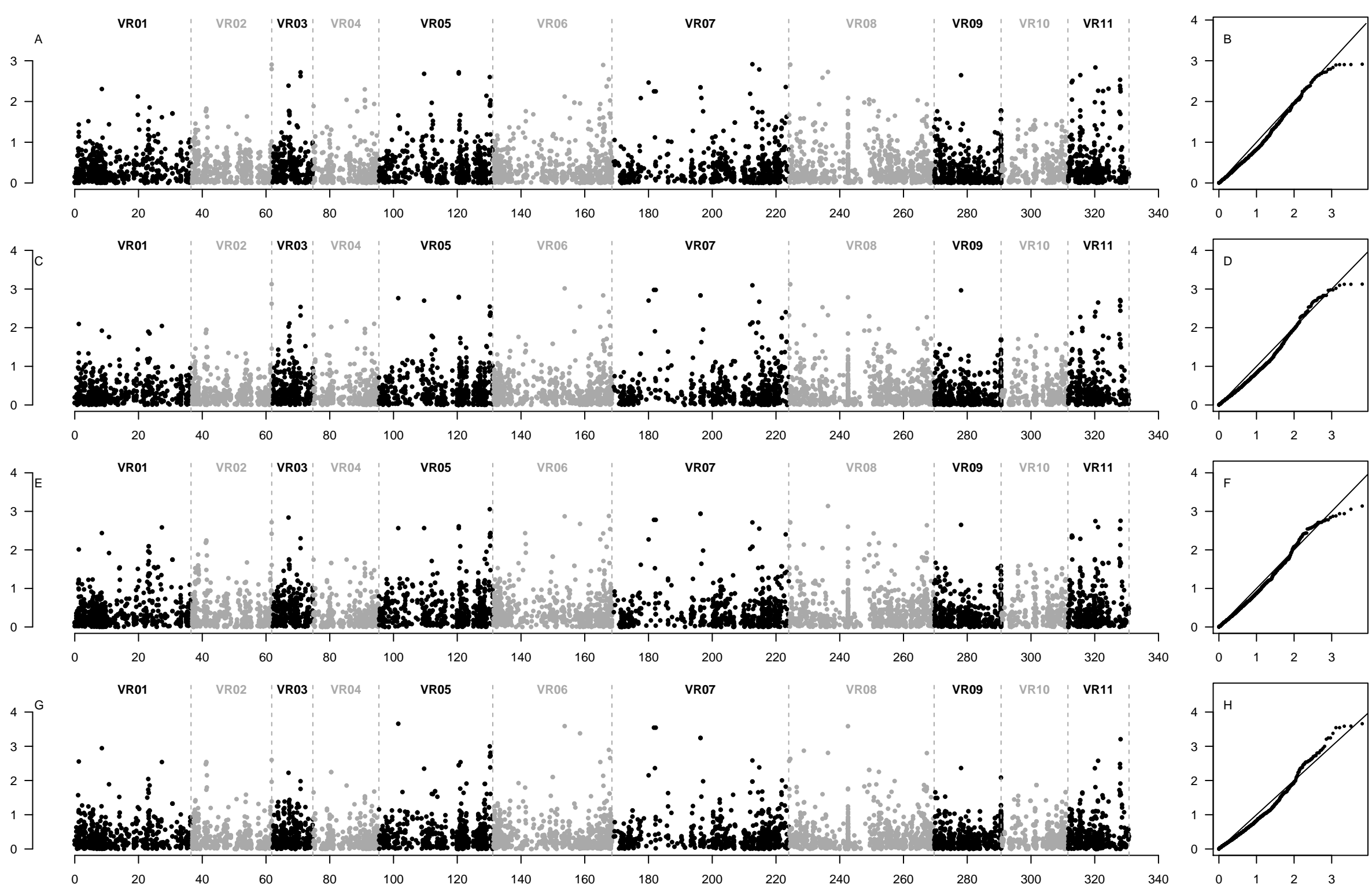

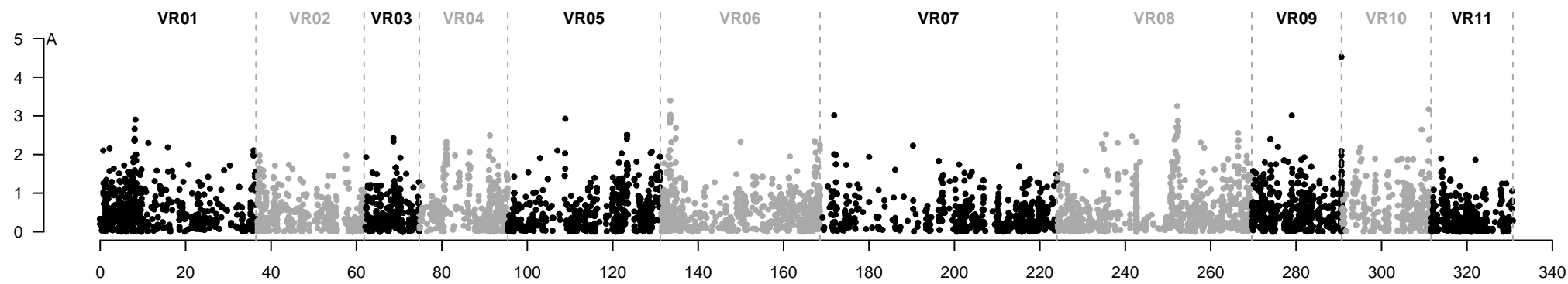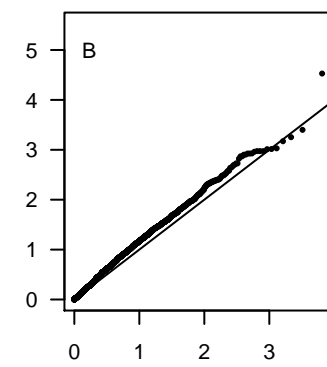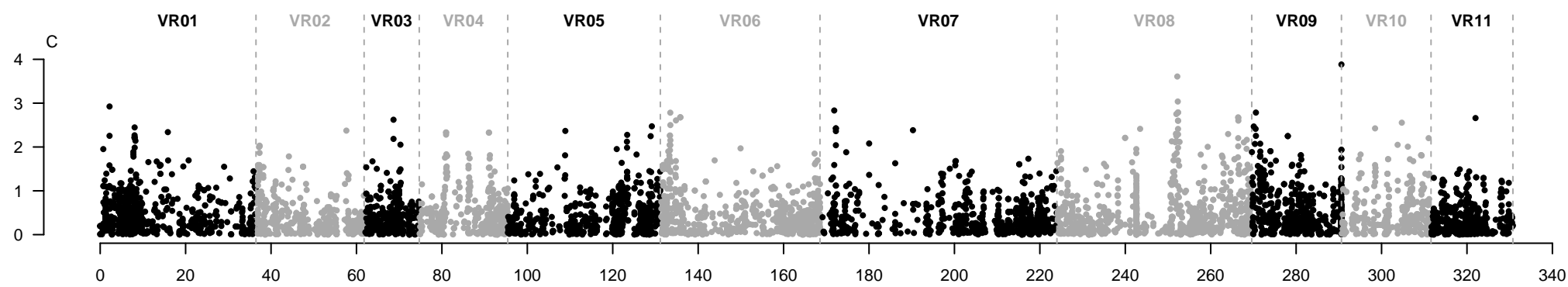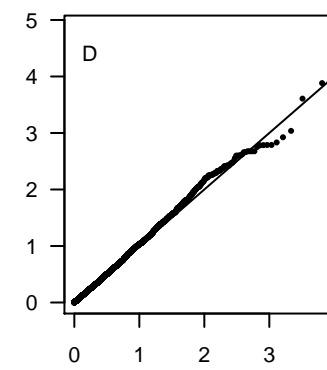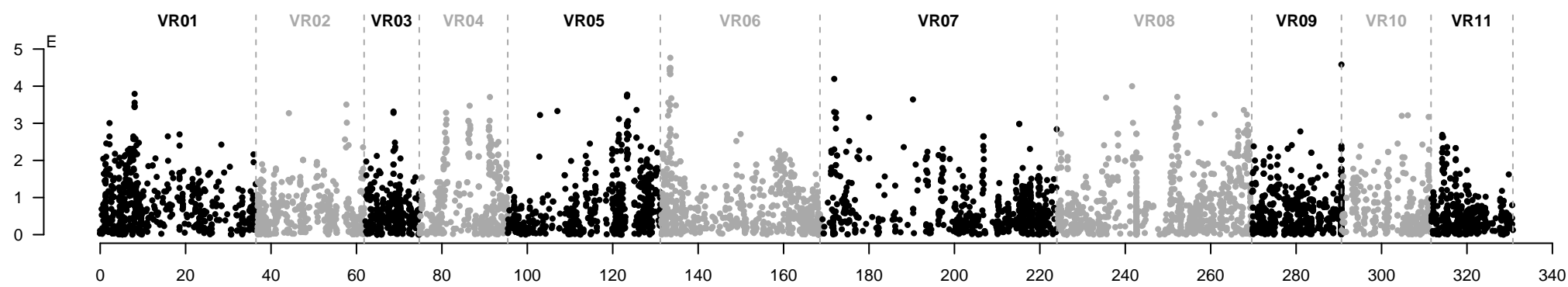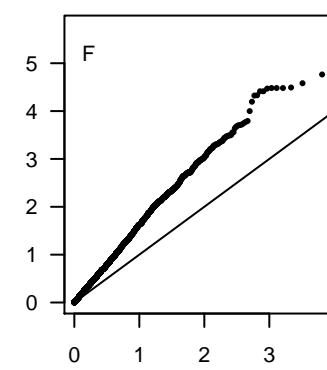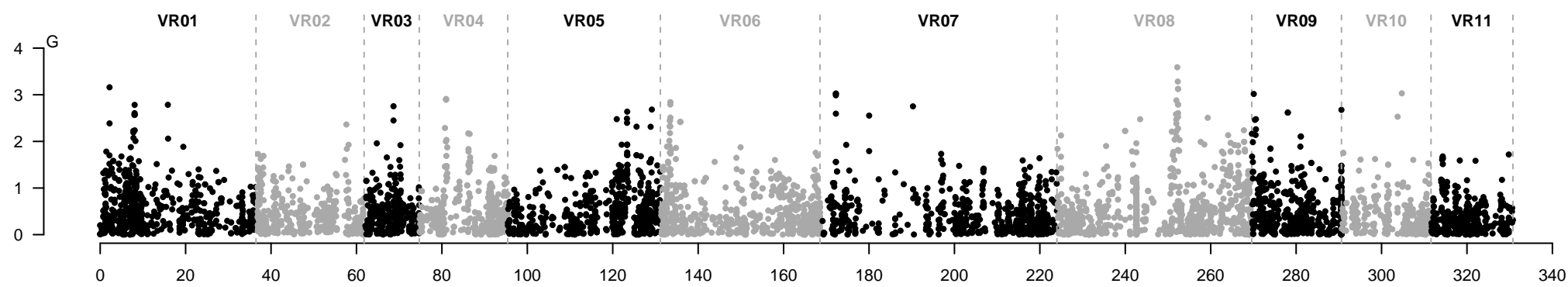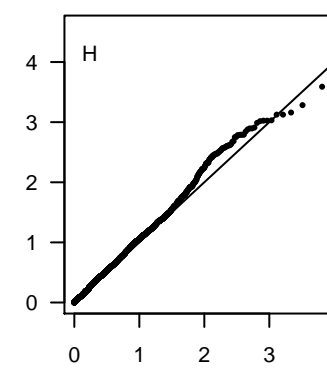

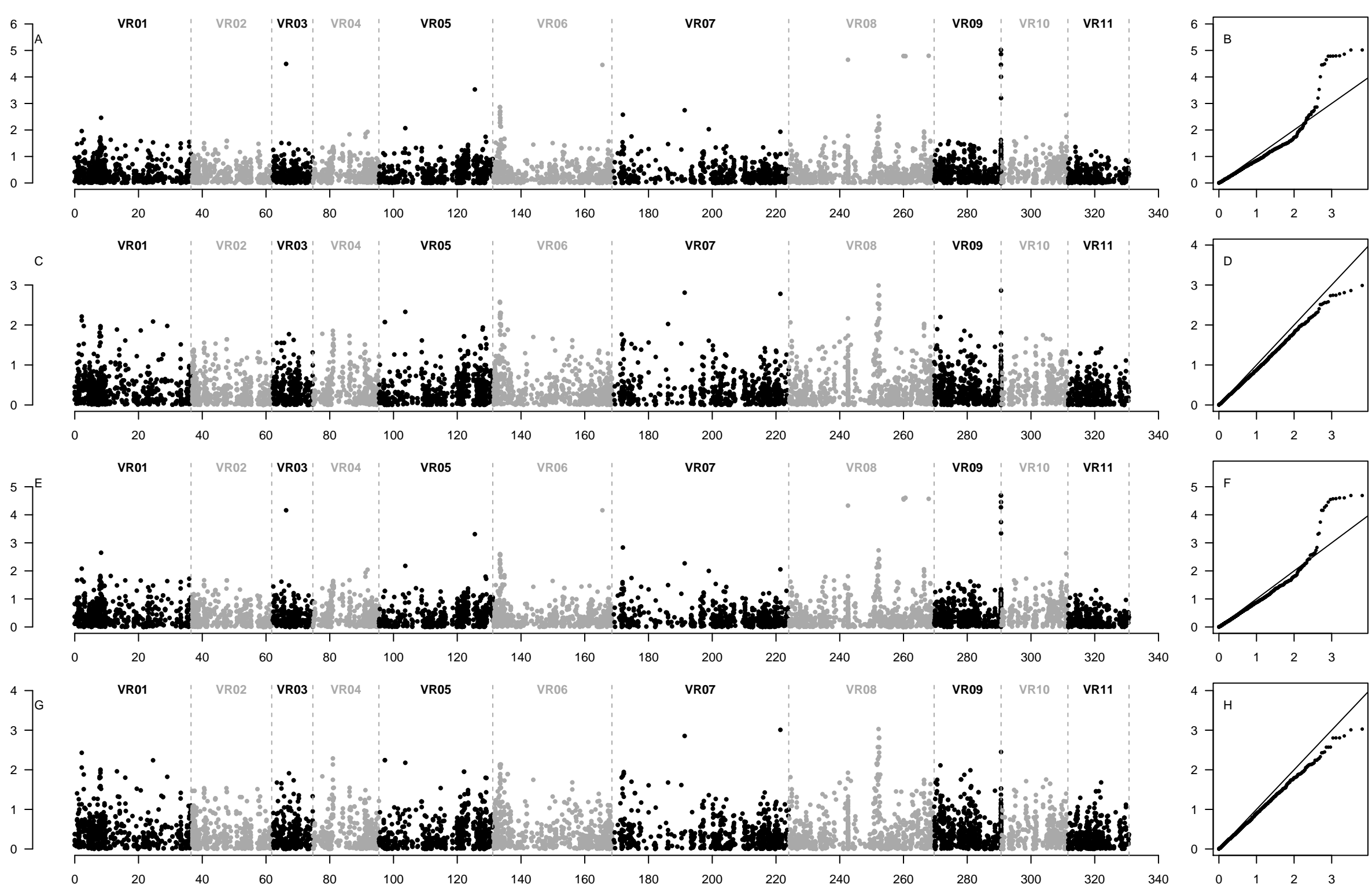

Supplement: Supplementary file 7 [file Data_Sheet_7.PDF]
