# Supplementary material for: Genome-Wide SNP Identification and Association Mapping for Seed Mineral Concentration in Mung Bean (Vigna radiata L.)
Source: Front Genet. 2020 Jun 24;11:656. doi: 10.3389/fgene.2020.00656 (PMC7327122; doi:10.3389/fgene.2020.00656)
Supplement: Supplementary file 8 [file Presentation_1.pdf]

Supplemental Figures (1-7). Genome-wide association analyses for 95 accessions of mung bean (*Vigna radiata*) for the following seed mineral concentrations: 1) calcium (Ca), 2) iron (Fe), 3) potassium (K), 4) manganese (Mn), 5) phosphorus (P), 6) sulfur (S) and 7) zinc (Zn) as measured in two different years (2015 and 2016) based on 6,486 SNP markers discovered through Genotyping by Sequencing (GBS). The gray dashed horizontal lines mark the P-value threshold after Bonferroni-correction for multiple tests. Black and gray colors highlight the 11 different mung bean (VR) chromosomes of the Phytozome genome sequence for *Vigna radiata* v1.0. Gray vertical boxes indicate the 1 Mb flanking region of each marker that was associated. Manhattan plots (A, C, E, G) and QQ-plots (B, D, F, H) of –log10(P-value) are displayed for the following models: (A, B) GLM or MLM with the country of origin and the first two PCoA axes scores (from Figure 3) as covariates; (C, D) GLM or MLM with the first two PCoA axes scores (from Figure 3) as covariates; (E, F) GLM or MLM with the country of origin as covariate; and (G, H) GLM or MLM without covariates.

Footnote for Suppl Figures: In each case first two pages of each file correspond to mineral concentration measurements made in 2015, while second two pages correspond to measurements in 2016. General linear models (GLMs) are depicted in pages 1 and 3, and mixed linear models (MLMs) with a centered IBS kinship matrix as a random effect are depicted in pages 2 and 4.
