# Supplementary material for: Genome-Wide SNP Identification and Association Mapping for Seed Mineral Concentration in Mung Bean (Vigna radiata L.)
Source: Front Genet. 2020 Jun 24;11:656. doi: 10.3389/fgene.2020.00656 (PMC7327122; doi:10.3389/fgene.2020.00656)
Supplement: Supplementary file 9 [file Table_1.docx]

**Table S1**. List of United States Department of Agriculture (USDA) mung bean (*Vigna radiata*) accessions used in this study. The genotype identification number according to USDA plant introduction (PI) and the country of origin are shown.

| **Code** | **PI** | **Species** | **Origin** | **Code** | **PI** | **Species** | **Origin** |
| --- | --- | --- | --- | --- | --- | --- | --- |
| 1 | PI425491 | *Vigna radiata* | India | 47 | PI183407 | *Vigna radiata* | India |
| 2 | PI425239 | *Vigna radiata* | Philippines | 48 | PI171435 | *Vigna radiata* | China |
| 3 | PI381144 | *Vigna radiata* | Thailand | 49 | PI425917 | *Vigna radiata* | Iran |
| 4 | PI183063 | *Vigna radiata* | India | 50 | PI473907 | *Vigna radiata* | Iran |
| 5 | PI364230 | *Vigna radiata* | India | 51 | PI377337 | *Vigna radiata* | India |
| 6 | PI432313 | *Vigna radiata* | Iran | 52 | PI383318 | *Vigna radiata* | Argentina |
| 7 | PI425330 | *Vigna radiata* | India | 53 | PI164726 | *Vigna radiata* | India |
| 8 | PI363445 | *Vigna radiata* | India | 54 | PI425578 | *Vigna radiata* | SouthKorea |
| 9 | PI473745 | *Vigna radiata* | Iran | 55 | PI174887 | *Vigna radiata* | India |
| 10 | PI473902 | *Vigna radiata* | Iran | 56 | PI371816 | *Vigna radiata* | Thailand |
| 11 | PI425570 | *Vigna radiata* | SouthKorea | 57 | PI164745 | *Vigna radiata* | India |
| 12 | PI473842 | *Vigna radiata* | Iran | 58 | PI473849 | *Vigna radiata* | Iran |
| 13 | PI377207 | *Vigna radiata* | India | 59 | PI377208 | *Vigna radiata* | India |
| 14 | PI205137 | *Vigna radiata* | India | 60 | PI502555 | *Vigna radiata* | Uzbekistan |
| 15 | PI313206 | *Vigna radiata* | Mexico | 61 | PI271407 | *Vigna radiata* | India |
| 16 | PI425565 | *Vigna radiata* | SouthKorea | 62 | PI473694 | *Vigna radiata* | Iran |
| 17 | PI425228 | *Vigna radiata* | Philippines | 63 | PI425378 | *Vigna radiata* | India |
| 18 | PI237669 | *Vigna radiata* | India | 64 | PI473861 | *Vigna radiata* | Iran |
| 19 | PI473859 | *Vigna radiata* | Iran | 65 | PI473610 | *Vigna radiata* | Iran |
| 20 | PI470228 | *Vigna radiata* | Taiwan | 66 | PI473676 | *Vigna radiata* | Iran |
| 21 | PI425557 | *Vigna radiata* | SouthKorea | 67 | PI473815 | *Vigna radiata* | Iran |
| 22 | PI473871 | *Vigna radiata* | Iran | 68 | PI269524 | *Vigna radiata* | Pakistan |
| 23 | PI426065 | *Vigna radiata* | India | 69 | PI378039 | *Vigna radiata* | India |
| 24 | PI425825 | *Vigna radiata* | Philippines | 70 | PI425484 | *Vigna radiata* | India |
| 25 | PI363469 | *Vigna radiata* | India | 71 | PI478619 | *Vigna radiata* | Iran |
| 26 | PI183937 | *Vigna radiata* | India | 72 | PI368324 | *Vigna radiata* | India |
| 27 | PI473718 | *Vigna radiata* | Iran | 73 | PI473835 | *Vigna radiata* | Iran |
| 28 | PI145943 | *Vigna radiata* | US | 74 | PI425934 | *Vigna radiata* | Iran |
| 29 | PI473727 | *Vigna radiata* | Iran | 75 | PI425592 | *Vigna radiata* | Philippines |
| 30 | PI291365 | *Vigna radiata* | China | 76 | PI425583 | *Vigna radiata* | SouthKorea |
| 31 | PI473671 | *Vigna radiata* | Iran | 77 | PI363444 | *Vigna radiata* | India |
| 32 | PI473741 | *Vigna radiata* | Iran | 78 | PI163110 | *Vigna radiata* | India |
| 33 | PI213012 | *Vigna radiata* | India | 79 | PI425943 | *Vigna radiata* | Iran |
| 34 | PI363389 | *Vigna radiata* | India | 80 | PI473918 | *Vigna radiata* | Iran |
| 35 | PI363783 | *Vigna radiata* | India | 81 | PI376925 | *Vigna radiata* | Philippines |
| 36 | PI425257 | *Vigna radiata* | India | 82 | PI473923 | *Vigna radiata* | Iran |
| 37 | PI425425 | *Vigna radiata* | India | 83 | PI473930 | *Vigna radiata* | Iran |
| 38 | PI377300 | *Vigna radiata* | India | 84 | PI425267 | *Vigna radiata* | Philippines |
| 39 | PI473790 | *Vigna radiata* | Iran | 85 | PI426151 | *Vigna radiata* | Afghanistan |
| 40 | PI426147 | *Vigna radiata* | India | 86 | PI432331 | *Vigna radiata* | Iran |
| 41 | PI426136 | *Vigna radiata* | India | 87 | PI425813 | *Vigna radiata* | SouthKorea |
| 42 | PI164301 | *Vigna radiata* | India | 88 | PI425797 | *Vigna radiata* | SouthKorea |
| 43 | PI363354 | *Vigna radiata* | India | 89 | PI473775 | *Vigna radiata* | Iran |
| 44 | PI473854 | *Vigna radiata* | Iran | 90 | PI473636 | *Vigna radiata* | Iran |
| 45 | PI179954 | *Vigna radiata* | India | 91 | PI425283 | *Vigna radiata* | Turkey |
| 46 | PI425821 | *Vigna radiata* | India | 92 | PI363753 | *Vigna radiata* | India |
