# Supplementary material for: Genome-Wide SNP Identification and Association Mapping for Seed Mineral Concentration in Mung Bean (Vigna radiata L.)
Source: Front Genet. 2020 Jun 24;11:656. doi: 10.3389/fgene.2020.00656 (PMC7327122; doi:10.3389/fgene.2020.00656)
Supplement: Supplementary file 10 [file Table_2.docx]

**Table S2**. List of candidate genes found within regions containing 43 SNP markers associated with seed mineral concentrations for seven elements in mung bean grown over two different years. Traits are listed within the categories macronutrients, macronutrient and other important elements. Marker names are given as chromosome and position on the reference genome. Significant loci were found by genome wide association tests using the 6,486 SNP markers found through genotyping by sequencing (GBS) analysis of 95 mung bean accessions. The F statistic, the P-value, the -log10 (P-value) and the variation explained (R^2^) by each marker, are shown. Gene names and their corresponded gene function according to annotation of *Vigna radiata* genome v1.0.

| **Number/**  **Region** | **Trait** | **Chr** | **Marker Name** | **F-value** | **P-value** | **LOD**  **value** | **R2** | **Gene Name** | **Gene Function** |
| --- | --- | --- | --- | --- | --- | --- | --- | --- | --- |
| **Macronutrients** | |  |  |  |  |  |  |  |  |
| 1 | P_2016 | Vr01 | SVR01_1387204 | 18.15 | 3.60E-07 | 6.44 | 0.29 | *Vradi01g00820* | 40S ribosomal protein S12 n |
|  | K_2016 | Vr01 | SVR01_1387204 | 17.30 | 6.43E-07 | 6.19 | 0.28 |  |  |
| 2 | P_2016 | Vr01 | SVR01_1392172 | 13.22 | 1.18E-05 | 4.93 | 0.23 | *Vradi01g00830* | protein farnesyltransferase subunit beta [Glycine max]; (regulation of cell proliferation) |
|  | K_2016 | Vr01 | SVR01_1392172 | 12.71 | 1.73E-05 | 4.76 | 0.22 |  |  |
| 3 | P_2016 | Vr01 | SVR01_1401760 | 18.28 | 3.30E-07 | 6.48 | 0.29 | *Vradi01g00840* | formate dehydrogenase (oxidation-reduction process) |
|  | K_2016 | Vr01 | SVR01_1401760 | 17.39 | 6.05E-07 | 6.22 | 0.28 |  |  |
| 4 | P_2016 | Vr05 | SVR05_25211011 | 19.97 | 1.07E-07 | 6.97 | 0.31 | *Vradi05g16350* | uncharacterized protein LOC100811711 [Glycine max] |
|  | K_2016 | Vr05 | SVR05_25211011 | 20.24 | 8.98E-08 | 7.05 | 0.31 |  |  |
| 5 | P_2015 | Vr07 | SVR07_49754942 | 14.57 | 3.50E-06 | 5.46 | 0.23 | *Vradi07g26320* | BTB/POZ domain-containing protein [Glycine max] |
|  | S_2015 | Vr07 | SVR07_49754942 | 11.10 | 6.09E-05 | 4.22 | 0.18 |  |  |
| 6 | K_2015 | Vr07 | SVR07_33824920 | 24.01 | 4.98E-09 | 8.30 | 0.35 | *Vradi07g14180* | Glutamyl-tRNA reductase family protein; (oxidation-reduction process |
|  | K_2015 | Vr07 | SVR07_33825176 | 15.78 | 1.42E-06 | 5.85 | 0.26 |  |  |
| 7 | K_2015 | Vr08 | SVR08_45006252 | 21.73 | 2.22E-08 | 7.65 | 0.33 | *Vradi08g22740* | Protein phosphatase 2C family protein; IPR001932 (Protein phosphatase 2C (PP2C)-like domain), |
|  | K_2015 | Vr08 | SVR08_45006217 | 21.73 | 2.22E-08 | 7.65 | 0.33 |  |  |
| 8 | K_2015 | Vr08 | SVR08_38988189 | 16.09 | 1.13E-06 | 5.95 | 0.26 | *Vradi08g17100* | ATP synthase D chain, mitochondrial; (ATP synthesis coupled proton transport) |
| 9 | P_2015 | Vr07 | SVR07_47951933 | 15.04 | 2.46E-06 | 5.61 | 0.24 | *none* | NA |
| 10 | P_2015 | Vr07 | SVR07_49780103 | 13.58 | 7.38E-06 | 5.13 | 0.22 | *Vradi07g26340* | DEAD-box ATP-dependent RNA helicase (ATP-dependent helicase activity) |
| 11 | P_2015 | Vr07 | SVR07_49780128 | 13.58 | 7.38E-06 | 5.13 | 0.22 |  |  |
| **Micronutrients** | |  |  |  |  |  |  |  |  |
| 12 | Fe_2016 | Vr06 | SVR06_21630747 | 13.36 | 1.11E-05 | 4.95 | 0.13 | *Vradi06g09900* | zinc finger CCCH domain-containing protein 38-like isoform X4 [Glycine max] |
| 13 | Fe_2016 | Vr06 | SVR06_21903341 | 15.13 | 3.10E-06 | 5.51 | 0.14 | *Vradi06g10020* | metal-nicotianamine transporter YSL3-like isoform X1 [Glycine max] |
| 14 | Fe_2016 | Vr06 | SVR06_22114556 | 15.19 | 2.98E-06 | 5.53 | 0.14 | *Vradi06g10060* | bZIP transcription factor family protein; (sequence-specific DNA binding) |
| 15 | Fe_2016 | Vr06 | SVR06_22271143 | 13.36 | 1.11E-05 | 4.95 | 0.13 | *Vradi06g10120* | Sulfite exporter TauE/SafE family protein (integral component of membrane) |
| 16 | Fe_2016 | Vr06 | SVR06_22461411 | 12.29 | 2.46E-05 | 4.61 | 0.12 | *Vradi06g10210* | ATP-dependent helicases;nucleic acid binding (DNA-directed DNA polymerases) |
| 17 | Zn_2015 | Vr01 | SVR01_8497478 | 11.30 | 4.33E-05 | 4.36 | 0.20 | *Vradi01g05570* | GAGA-binding protein isoform X3 [Glycine max] GAGA-binding transcriptional activator |
| 18 | Zn_2016 | Vr06 | SVR06_2352372 | 11.89 | 3.22E-05 | 4.49 | 0.22 | *none* | NA |
| 19 | Zn_2015 | Vr07 | SVR07_13191880 | 11.49 | 3.74E-05 | 4.43 | 0.20 | *Vradi07g05940* | protein YLS7-like [Glycine max]; (PC-Esterase) |
| 20 | Zn_2015 | Vr07 | SVR07_13740249 | 11.49 | 3.74E-05 | 4.43 | 0.20 | *Vradi07g06200* | Iron-sulfur cluster assembly protein  SufB n |
| 21 | Zn_2015 | Vr08 | SVR08_12313446 | 9.21 | 2.35E-04 | 3.63 | 0.17 | *none* | NA |
| 22 | Zn_2016 | Vr06 | SVR06_2334764 | 12.72 | 1.72E-05 | 4.77 | 0.24 | *Vradi06g02380* | Pleckstrin homology (PH) domain-containing protein |
| 23 | Zn_2016 | Vr09 | SVR09_21001271 | 20.02 | 2.62E-05 | 4.58 | 0.19 | *none* | NA |
| **Other Important Elements** | | | |  |  |  |  |  |  |
| 24 | S_2015 | Vr07 | SVR07_44411453 | 10.92 | 7.00E-05 | 4.15 | 0.18 | *Vradi07g21720* | protein FLX-like 1-like isoform X1 [Glycine max] |
| 25 | S_2015 | Vr09 | SVR09_7847978 | 10.93 | 6.93E-05 | 4.16 | 0.18 | *Vradi09g05410* | transforming growth factor-beta receptor-associated protein 1 homolog isoform X1 [Glycine max];(Vacuolar sorting protein 39/Transforming growth factor beta receptor-associated domain 2) |
| 26 | S_2015 | Vr07 | SVR07_41344562 | 14.69 | 4.24E-06 | 5.37 | 0.22 | *Vradi07g19370* | cytochrome P450, family 718 (iron ion binding, heme binding, redox activity) |
| 27 | S_2016 | Vr08 | SVR08_36596365 | 20.03 | 1.41E-09 | 8.85 | 0.01 | *Vradi08g16110* | U3 small nucleolar RNA-associated-like protein, putative; (WD40/YVTN repeat-like-containing domaim, protein binding) |
|  | S_2016 | Vr08 | SVR08_36596397 | 20.03 | 1.41E-09 | 8.85 | 0.01 |  |  |
| 28 | S_2016 | Vr08 | SVR08_36072603 | 19.89 | 1.59E-09 | 8.80 | 0.01 | *Vradi08g15850* | exocyst subunit exo70 family protein A1; (Cullin repeat-like-containing domain); |
| 29 | S_2016 | Vr08 | SVR08_36006946 | 19.59 | 2.03E-09 | 8.69 | 0.00 | *Vradi08g15760* | uncharacterized protein [Glycine max] |
| 30 | S_2016 | Vr08 | SVR08_43903167 | 20.12 | 1.32E-09 | 8.88 | 0.02 | *none* | NA |
| 31 | Mn_2016 | Vr01 | SVR01_22929122 | 17.55 | 3.96E-07 | 6.40 | 0.38 | *Vradi01g11650* | glycogen/starch/alpha-glucan phosphorylase family protein; (Glycosyl transferase, family 35, (glycogen phosphorylase activity) |
| 32 | Mn_2016 | Vr08 | SVR08_18615379 | 17.89 | 3.10E-07 | 6.51 | 0.39 | none | NA |
| 33 | Mn_2016 | Vr08 | SVR08_18582837 | 17.33 | 4.61E-07 | 6.34 | 0.38 | none | NA |
| 34 | Mn_2016 | Vr09 | SVR09_20969884 | 17.89 | 3.10E-07 | 6.51 | 0.39 | none | NA |
| 35 | Mn_2016 | Vr09 | SVR09_20995726 | 17.93 | 3.02E-07 | 6.52 | 0.39 | none | NA |
